# Phylogenomic and Comparative Genomic Analyses Reveal Deep Evolutionary Structure and Cryptic Diversity in the Genus *Trichoderma*

**DOI:** 10.3390/ijms27188204

**Published:** 2026-09-15

**Authors:** Felipe Cabarcas, Juliana Lopez-Jimenez, Maria Patricia Ricardo, Isabel Luna, Juan F. Alzate

**Affiliations:** 1 Centro Nacional de Secuenciación Genómica—CNSG, Sede de Investigación Universitaria—SIU, Universidad de Antioquia UdeA, Calle 67 No. 53-108, Medellín 050010, Colombia; felipe.cabarcas@udea.edu.co (F.C.); extension.cnsg@udea.edu.co (J.L.-J.); 2 Facultad de Ingeniería, Universidad de Antioquia UdeA, Calle 70 No. 52-21, Medellín 050010, Colombia; 3 Bioorigen, Grupo Greenland, Apartado 057840, Colombia; maria.ricardo@bioorigen.co (M.P.R.); isabel.luna@bioorigen.co (I.L.); 4 Departamento de Microbiología y Parasitología, Facultad de Medicina, Universidad de Antioquia UdeA, Calle 70 No. 52-21, Medellín 050010, Colombia

**Keywords:** *Trichoderma*, comparative genomics, phylogenomic analysis, evolution, taxogenomics

## Abstract

The genus *Trichoderma* comprises ecologically and biotechnologically important fungi that have been widely investigated and used in agriculture, industrial biotechnology, and biological control. However, publicly available genomes reveal substantial taxonomic inconsistencies across the genus, which can complicate strain identification, reproducibility, and the comparison and selection of strains for applied research and biotechnology. Here, we present a comprehensive phylogenomic and comparative genomic analysis integrating one of the largest collections of *Trichoderma* genomes analyzed to date. Phylogenomic reconstruction based on 920 conserved single-copy orthologs recovered four major evolutionary clades with strong statistical support and revealed widespread taxonomic inconsistencies affecting multiple species complexes, including *T. harzianum*, *T. asperellum*, *T. viride*, and *T. longibrachiatum*. Comparative analyses demonstrated marked clade-associated differences in genome size, GC content, repetitive DNA content, gene content, and whole-genome conservation patterns. Genome size was positively associated with repetitive-element accumulation and gene number, whereas GC content showed a negative association with genome size. We additionally characterized a novel Colombian isolate that clustered within a highly supported and divergent lineage together with three inconsistently annotated public genomes. This lineage, provisionally designated *Trichoderma* sp. “CB2”, formed a sister group to the Longibrachiatum complex and exhibited strong internal genomic cohesion and clear divergence from neighboring lineages. Orthology-based analyses identified lineage-associated proteins with predicted functions related to transcriptional regulation, plant biomass degradation, secondary metabolism, and detoxification. Overall, this study provides a genome-scale framework for resolving *Trichoderma* diversity and highlights the extent of taxonomic inconsistencies in public genomic resources. Improved phylogenomic characterization of strains can facilitate more reliable strain identification, reproducibility, comparative genomic studies, and the selection and evaluation of *Trichoderma* strains for agricultural and biotechnological applications.

## 1. Introduction

Species of the genus *Trichoderma* are among the most economically and biotechnologically important filamentous fungi, with applications spanning agriculture, biocontrol, enzyme production, and environmental biotechnology [1]. Despite this importance, the taxonomy of *Trichoderma* has historically been affected by extensive species misidentification and unstable classification frameworks [2]. The high morphological similarity among species, together with the historical reliance on low-resolution phenotypic traits and single-marker approaches, led to decades of taxonomic ambiguity, particularly within species-rich lineages such as the Harzianum, Viride, and Longibrachiatum species complexes [3]. Consequently, as observed in other fungi, numerous strains deposited in culture collections and genomic repositories have been incorrectly annotated, propagating inconsistencies across ecological, physiological, and industrial studies. These taxonomic inaccuracies have complicated comparative analyses, obscured species boundaries, and in several cases, delayed the recognition of new species [4].

One of the most emblematic examples of this problem is *Trichoderma reesei*, currently regarded as a premier industrial workhorse due to its extraordinary cellulolytic capabilities. The type strain of *T. reesei*, QM6a, was isolated during World War II after United States military personnel stationed in the Solomon Islands observed the rapid degradation of cotton-based materials, such as tents and uniforms, under tropical conditions [5]. Investigation of this biodegradation process led to the isolation of a highly cellulolytic green filamentous fungus, which was subsequently transferred to the U.S. Army Quartermaster Research and Development Center in Natick, Massachusetts, where the strain received the designation “QM6a” [6,7]. The fungus rapidly attracted attention because of its remarkable cellulase production capacity, eventually becoming a protagonist of modern industrial cellulase biotechnology. Indeed, most industrial cellulase-producing strains currently utilized in biorefineries and enzyme production platforms are hyper-secreting mutant derivatives obtained through successive rounds of random mutagenesis of this original isolate [8,9].

However, despite its industrial relevance, the taxonomic identity of QM6a remained uncertain for decades. Initially, the isolate was classified as *Trichoderma viride* based on superficial morphological similarities to existing taxa. Only in 1977 did Emory G. Simmons recognize that QM6a displayed consistent morphological and physiological differences from authentic *T. viride* strains, formally describing it as a new species, *Trichoderma reesei*, in honor of Elwyn T. Reese, one of the pioneering researchers involved in its physiological characterization. Subsequent phylogenetic and molecular fingerprinting analysis demonstrated that *T. reesei* corresponds to the anamorphic (asexual) stage of the pantropical ascomycete *Hypocrea jecorina* [10]. For many years, both names coexisted under the traditional dual nomenclature system used in fungal taxonomy, where sexual and asexual morphs were assigned separate scientific names. The subsequent adoption of the “one fungus, one name” convention by the International Botanical Congress eliminated this dual system, establishing *Trichoderma reesei* as the single, accepted species name [8].

The history of *T. reesei* illustrates how inaccurate or incomplete taxonomy can persist even in some of the most intensively studied fungal systems. Similar obstacles continue to affect multiple *Trichoderma* lineages, where cryptic diversity, strain cross-contamination, mixed assemblies, and inconsistent automated annotations remain common in publicly available datasets. The rapid expansion of whole-genome sequencing (WGS) now provides an unprecedented opportunity to resolve these long-standing inconsistencies through phylogenomics and comparative genomics [11]. Genome-scale analyses based on hundreds of conserved orthologous loci offer substantially greater discriminatory power than traditional single-locus barcode markers, enabling robust species delimitation and a more accurate reconstruction of evolutionary relationships [12]. At the same time, the reliability of these macro-approaches depends critically on rigorous genome quality control, including the explicit detection of contaminant contigs, mixed-strain assemblies, and incorrectly assigned taxonomy in public repositories [13].

Recent large-scale comparative studies have further emphasized the remarkable ecological and evolutionary diversity within the genus *Trichoderma*. For example, a recent phenogenomic analysis integrating genomic data from 37 *Trichoderma* strains with more than 140 phenotypic traits demonstrated that the genus is both genetically cohesive and physiologically highly diverse, exhibiting extensive variation in metabolic versatility, stress tolerance, dispersal strategies, and ecological adaptation. The study additionally highlighted evidence of convergent evolution and ecological specialization across distant lineages, reinforcing the importance of robust phylogenomic frameworks for accurately interpreting the evolutionary and functional diversification of *Trichoderma* species in agricultural and environmental contexts [14].

Due to the central role of *Trichoderma* fungi in modern sustainable agriculture and their widespread implementation as biocontrol agents, biofertilizers, and industrial enzyme factories, establishing a reliable, standardized, and universally accessible species-level taxonomic framework has become paramount for both academic researchers and industrial applications [15]. Because public repositories, such as the NCBI databases, constitute the primary global infrastructure for genetic and genomic information used in strain identification, comparative genomics, and taxonomic reference, the structural and nomenclature accuracy of these archived records directly impacts downstream biological interpretation, metabolic profiling, and commercial product development and regulation [16]. Historically, *Trichoderma* classification has relied on an integrative polyphasic system combining micro-morphology with definitive multilocus diagnostic markers such as *tef1* and *rpb2* [2,17]. However, the exponential influx of high-throughput sequencing data has outpaced manual curation, occasionally leading to misannotated or unverified assemblies in public repositories that can propagate inaccuracies in downstream analyses. In this context, establishing a robust, curated phylogenomic framework represents a natural and necessary extension of traditional barcoding protocols. By anchoring genomic data to validated type-strain data and classical evolutionary lineages, such a framework serves as a common, high-resolution reference point for the broader mycological and industrial communities, improving the detection of historical misidentifications, facilitating precise strain classification, and providing a standardized foundation for the discovery of truly novel *Trichoderma* [18].

In this study, we pursued two principal objectives. First, we aimed to establish a reliable precision-taxogenomics framework for *Trichoderma* by applying a contamination-filtered phylogenomic pipeline to hundreds of public assemblies deposited in NCBI repositories. Utilizing this robust baseline, we systematically audited the prevalence of historical misannotations in public databases to determine whether ambiguously cataloged strains, alongside a novel Colombian isolate recovered from a rural agricultural environment, represent recognized species or correspond to entirely undocumented evolutionary lineages. Second, building upon this curated evolutionary foundation, we investigated macro-genomic structural variation across the genus, quantifying how genome size, GC content, repetitive DNA landscape, and predicted gene space co-vary and differentiate among the four major *Trichoderma* clades.

## 2. Results

### 2.1. Genome Contamination Screening and Quality Control

Genome quality control and contaminant screening were performed using CAT to identify non-*Trichoderma* contigs within the genome assemblies. Among the 191 quality- and redundancy-filtered genomes selected from the NCBI Datasets repository, 83 showed no detectable contaminant contigs. Of the remaining assemblies, 20 contained contigs assigned to bacterial taxa, whereas 103 contained contigs classified as non-*Trichoderma* fungal sequences. In most cases, contamination levels were low and limited to a small number of contigs. Following contaminant removal, only minor changes in assembly metrics were observed, with the median genome size decreasing by 1.79% (Supplementary Figure S1, Supplementary Table S1), indicating that the filtered assemblies retained their overall genomic integrity while improving taxonomic consistency for downstream phylogenomic analyses. The most frequently detected contaminant taxa corresponded to filamentous fungi commonly associated with soil and plant environments, including *Fusarium*, *Metarhizium*, *Penicillium*, *Talaromyces*, *Ilyonectria*, *Pochonia*, and *Claviceps*. Additional contaminants included bacterial genera such as *Niallia*, *Achromobacter*, and Stenotrophomonas, as well as occasional assignments to yeast (*Maudiozyma*) and plant (*Oryza*) sequences. The recurrent detection of environmental fungi suggests that low-level co-isolation or assembly carryover may be common in publicly available *Trichoderma* genome datasets.

In this study, we included a novel Colombian isolate that was sequenced, assembled, and filtered using the same methodology applied to the reference genomes. The isolate was designated CB2, and CAT contamination analysis revealed that the assembly contained only eight contaminant contigs, predominantly assigned to fungal taxa, which were removed prior to downstream analyses.

The filtered *Trichoderma* genome assemblies exhibited high overall completeness and contiguity. BUSCO completeness showed a median value of 99.4%, ranging from 83% to 99.7%, while the median proportion of single-copy BUSCO markers was 90.8% (74.5–93.4%). Multicopy BUSCOs presented a median value of 8.3%, whereas fragmented and missing BUSCOs remained low, with median values of 0.1% and 0.5%, respectively. All analyses were performed using the same set of 3817 BUSCO markers.

Assembly contiguity metrics indicated generally high-quality genomes, with a median N50 value of 1,197,945 bp, ranging from 40,231 bp to 8,263,504 bp. Repeat content, estimated as the percentage of masked bases using RepeatModeler and RepeatMasker, showed moderate variation across genomes, ranging from 3.92% to 17.0%, with a median of 6.43%. Structural annotation statistics revealed a median of 12,129.5 transcripts per genome. The median number of exons per transcript was 2.99, indicating relatively compact gene architectures across the analyzed assemblies. Median gene length was 1408 bp, while median intron and exon lengths showed median values of 89.5 bp and 275 bp, respectively (Supplementary Table S2).

### 2.2. Phylogenomic Reconstruction Resolves Deep Lineages and Taxonomic Incongruencies in Trichoderma

Phylogenomic analyses were conducted using the 191 quality- and redundancy-filtered NCBI genomes, the newly sequenced and quality-filtered Colombian isolate *Trichoderma* sp. 2 “CB2”, and 16 additional non-redundant *Trichoderma* genomes retrieved from the JGI database following the publication of Steindorff et al. (2026) [14]. The latter genomes were included to increase taxonomic representation and incorporate lineages that were not represented in the NCBI dataset at the time of our final query. After integration of these datasets, the final phylogenomic dataset comprised 208 genomes.

**Figure 1:**
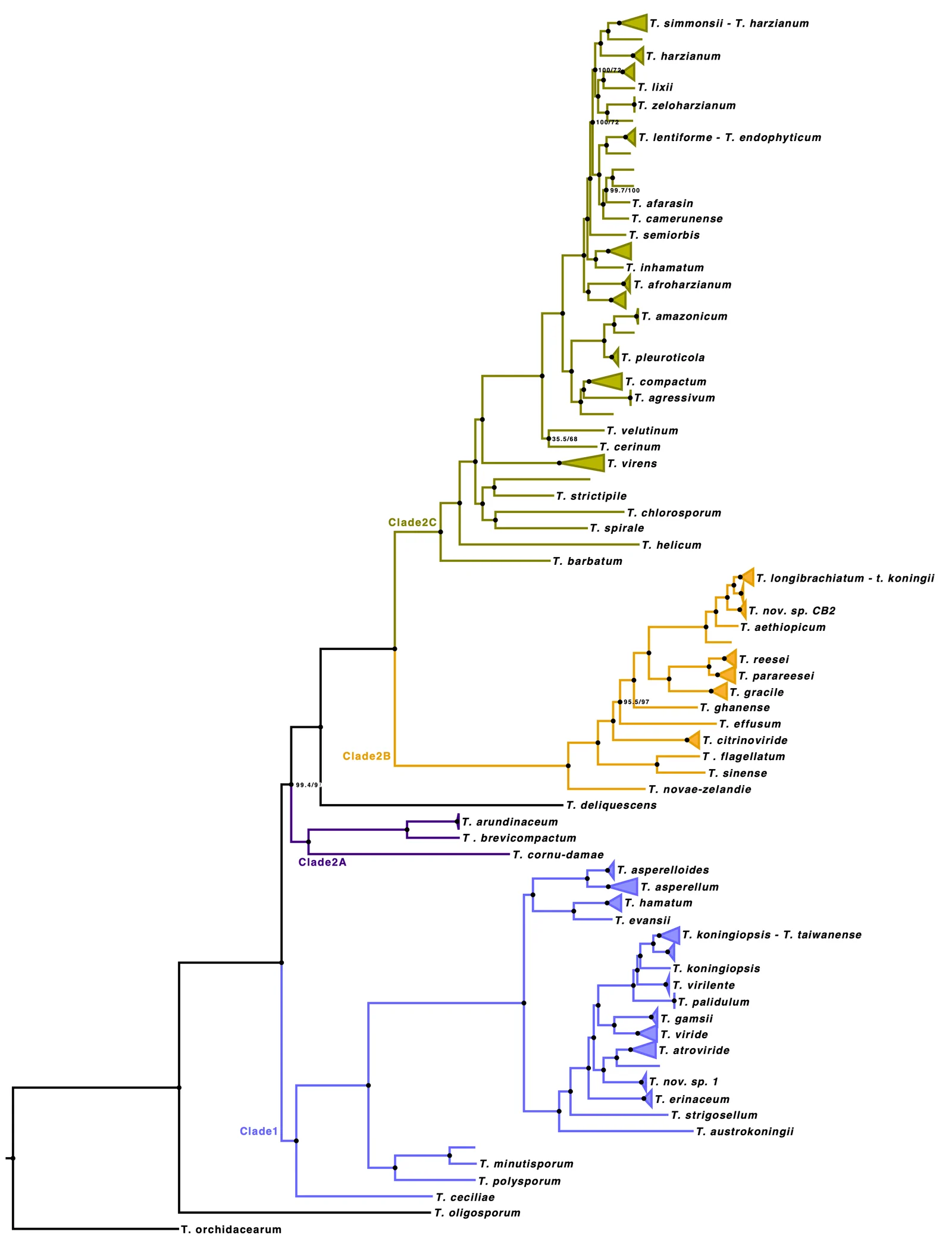
Maximum-likelihood phylogenomic tree of *Trichoderma* reconstructed using IQ-TREE from a concatenated alignment of 920 conserved CDS partitions. Branch support was assessed using 2000 SH-aLRT replicates and 5000 ultrafast bootstrap (UFBoot) replicates. The tree was rooted using *Trichoderma orchidacearum* strain COAD-3006 (GCA_023653515) as the outgroup. Major phylogenomic groups corresponding to Clade 1, Clade 2A, Clade 2B, and Clade 2C are highlighted with distinct colors. Black circles indicate nodes receiving maximal statistical support (100/100 SH-aLRT/UFBoot), whereas support values are explicitly shown for nodes with lower support. The phylogenetic tree was visualized and rendered using FigTree v1.4.4 (http://tree.bio.ed.ac.uk/software/figtree/ accessed on 7 November 2025).

The phylogenomic reconstruction, rooted with *Trichoderma orchidacearum*, resolved two major and supported evolutionary lineages within the genus. The first lineage, here designated Clade 1, included species such as *T. erinaceum*, *T. atroviride*, *T. viride*, *T. gamsii*, *T. palidulum*, *T. virilente*, *T. koningiopsis*, *T. evansii*, *T. hamatum*, *T. asperellum*, and *T. asperelloides*, together with several additional species represented by fewer isolates. The second clade, which is more diverse, comprises species including *T. arundinaceum*, *T. brevicompactum*, *T. citrinoviride*, *T. gracile*, *T. reesei*, *T. parareesei*, *T. longibrachiatum*, *T. virens*, *T. velitinum*, *T. aggressivum*, *T. amazonicum*, *T. afroharzianum*, *T. endophyticum*, *T. zeloharzianum*, *T. harzianum*, and *T. simmonsii*, along with several additional recognized species represented by fewer genomes. This broader lineage was further subdivided into three principal subclades, here designated Clades 2A, 2B, and 2C, which are described in detail below (Figure 1).

A complete expanded representation of the phylogenomic tree is available in Supplementary Figure S2. In general, most recognized species included in the analysis formed monophyletic groups with strong support (SH-aLRT = 100 and UFBoot = 100). However, several topological discordances suggest potential taxonomic inconsistencies within specific species complexes, or reflect limitations in our phylogenomic approach to resolve boundaries among closely related species. Additionally, our phylogenomic approach failed to resolve certain relationships within the clade that encompasses velutinum+cerinum and the clade zeloharzianum+harzianum+endophyticum with a few nodes with low support (UFB ≤ 95). Formal taxonomic revision or species delimitation would require additional evidence, potentially including broader population-level sampling, genomic divergence analyses, reproductive or ecological information, and dedicated species-delimitation approaches.

**Figure 2:**
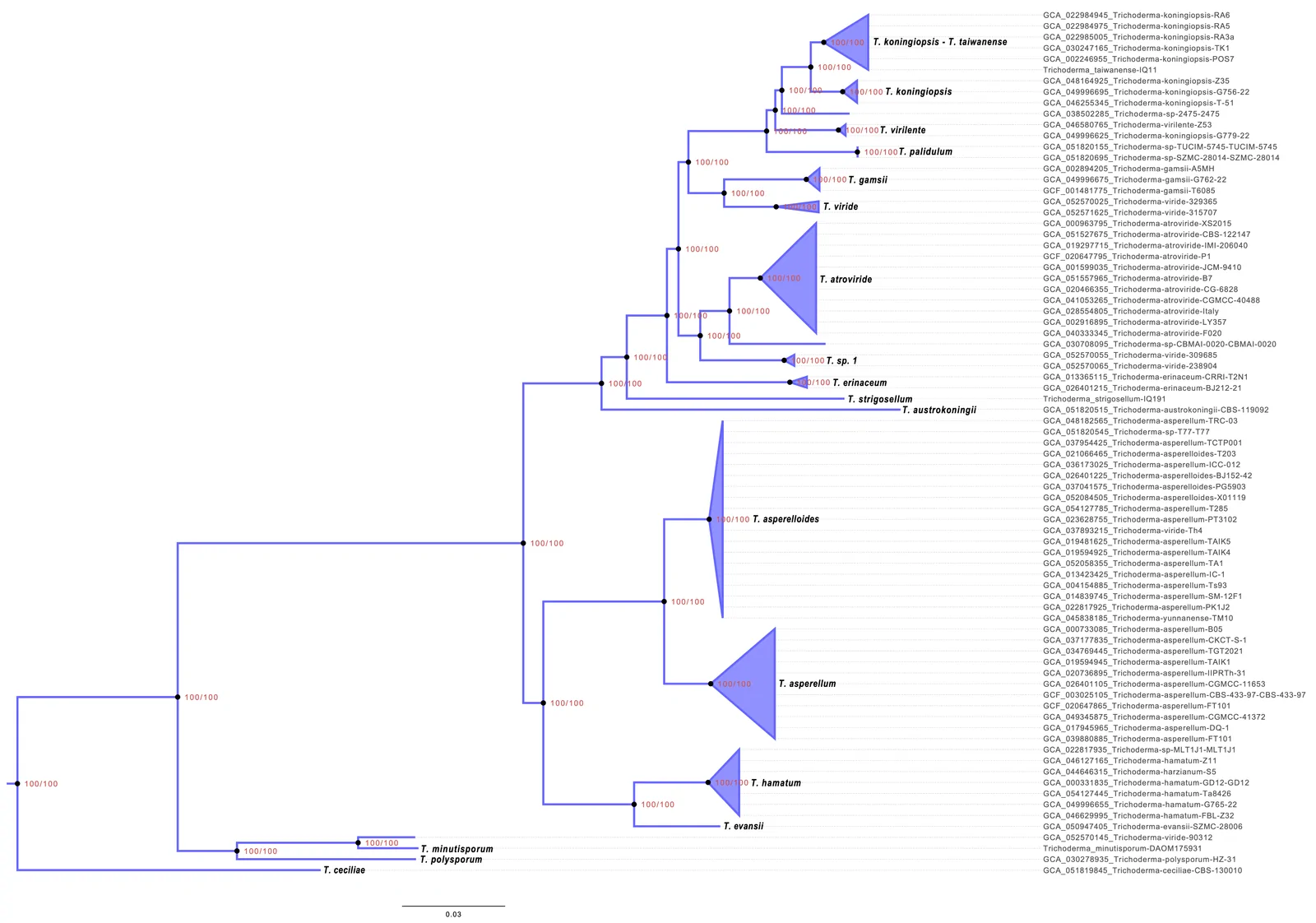
Maximum-likelihood phylogenomic tree corresponding to *Trichoderma* Clade 1, reconstructed using IQ-TREE from the concatenated alignment of 920 conserved CDS partitions. This tree represents a detailed subtree extracted from the global phylogenomic reconstruction shown in Figure 1 and was rooted using *Trichoderma ceciliae* strain CBS 130010 (GCA_051819845). Branch support values correspond to SH-aLRT/UFBoot support obtained from 2000 SH-aLRT replicates and 5000 ultrafast bootstrap replicates and are indicated at each node. Species-level lineages are represented as collapsed cartoon clades to facilitate visualization of the overall phylogenetic structure within Clade 1. The phylogenetic tree was visualized and rendered using FigTree v1.4.4 (http://tree.bio.ed.ac.uk/software/figtree/, accessed on 7 November 2025).

Phylogenomic reconstruction of *Trichoderma* Clade 1 recovered a highly resolved and supported topology, with most species-level lineages receiving maximal branch support (100/100 SH-aLRT/UFBoot). The clade was structured into two principal monophyletic lineages in addition to a basal assemblage comprising *Trichoderma minutisporum* and *Trichoderma polysporum*. This basal lineage also included accession GCA_052570145, currently annotated as *Trichoderma viride*, which was phylogenetically distant from the core *T. viride* lineage and therefore likely represents a taxonomically misidentified genome (Figure 2). An independent summary-coalescent analysis was performed using ASTER v1.25 (ASTRAL) based on the 920 single-copy ortholog gene trees used in the concatenated phylogenomic analysis. Local posterior probabilities (LPPs) were calculated for the resulting species tree and compared directly with the concatenated IQ-TREE topology and its SH-aLRT and ultrafast bootstrap (UFB) support values. The coalescent analysis recovered the same species-level topology and major basal divisions as the concatenated analysis (Supplementary Figure S3). In particular, Clade 1 (100% SH-aLRT/100% UFB/1.00 LPP) and Clades 2A, 2B, and 2C (each 100%/100%/1.00) were consistently recovered with strong support. Most species-level relationships also received maximal support in both analyses, including the *T. harzianum–T. simmonsii*, *T. longibrachiatum–T. koningii*, *T. koningiopsis*, *T. asperellum*, *T. asperelloides*, *T. atroviride*, *T. reesei*, *T. parareesei*, and *T. citrinoviride* lineages. The only notable reduction in support was observed for the *T. velutinum* + *T. cerinum* grouping (35.5% SH-aLRT/68% UFB/0.995 LPP). Clade 2 as a whole also showed lower coalescent support (99.4% SH-aLRT/99% UFB/0.54 LPP), whereas its three subdivisions were maximally supported. Overall, these results indicate that the principal species-level relationships and the basal division into Clades 1, 2A, 2B, and 2C are robust to both concatenation and summary-coalescent approaches.

The first major lineage encompassed the *Trichoderma hamatum* and Asperellum/Asperelloides complexes, including *Trichoderma evansii*, *Trichoderma hamatum*, *Trichoderma asperellum*, and *Trichoderma asperelloides*. The second principal lineage comprised species associated with the Viride, Atroviride, and Koningiopsis complexes, including *Trichoderma austrokoningii*, *Trichoderma strigosellum*, *Trichoderma erinaceum*, *Trichoderma atroviride*, *Trichoderma viride*, *Trichoderma gamsii*, *Trichoderma palidulum*, *Trichoderma virilente*, *Trichoderma koningiopsis*, and *Trichoderma taiwanense*. The topology recovered within Clade 1 showed broad correspondence with several previously recognized *Trichoderma* species complexes, although these historical groupings were not used as criteria for defining the phylogenomic clades and were not independently tested here as formal taxonomic units.

Notably, the phylogeny revealed substantial taxonomic incongruence within the *Trichoderma asperellum*–*Trichoderma asperelloides* complex. All genomes belonging to the core *T. asperellum* lineage were consistently annotated in NCBI as *T. asperellum* and clustered with the reference strain CBS 433.97 (GCF_003025105). In contrast, several genomes currently annotated as *T. asperellum*, together with the misplaced *Trichoderma viride* strain Th4 (GCA_037893215), were robustly nested within the monophyletic *T. asperelloides* lineage. This clade included the reference strain GCA_026401225 *Trichoderma asperelloides* BJ152-42 and was clearly separated from the core *T. asperellum* lineage. Additionally, accession GCA_045838185, currently annotated as *Trichoderma yunnanense*, was also placed within the *T. asperelloides* clade with minimal phylogenetic divergence from the remaining members of this group (Figure 2).

Our analysis also revealed two additional noteworthy phylogenetic patterns. First, two accessions currently annotated as *T. viride* (GCA_052570055 and GCA_052570065) formed an independent sister clade to *T. atroviride*, representing a supported monophyletic lineage (100/100 SH-aLRT/UFBoot) that is phylogenetically distinct from the core *T. viride* cluster. The marked divergence of this lineage suggests that these strains are likely misannotated in public repositories and may represent a distinct phylogenomic candidate lineage, here informally designated as *Trichoderma* sp. 1, pending future formal polyphasic characterization. Second, *T. taiwanense* was nested within the *T. koningiopsis* aggregate species complex, forming a sister lineage to specific strains annotated as *T. koningiopsis* in MycoBank. This topology highlights an intimate evolutionary affinity between these taxa, corroborating previous reports of high genomic similarity, and underscores the value of integrated morpho-molecular analyses to definitively resolve taxonomic boundaries within this aggregate complex.

**Figure 3:**
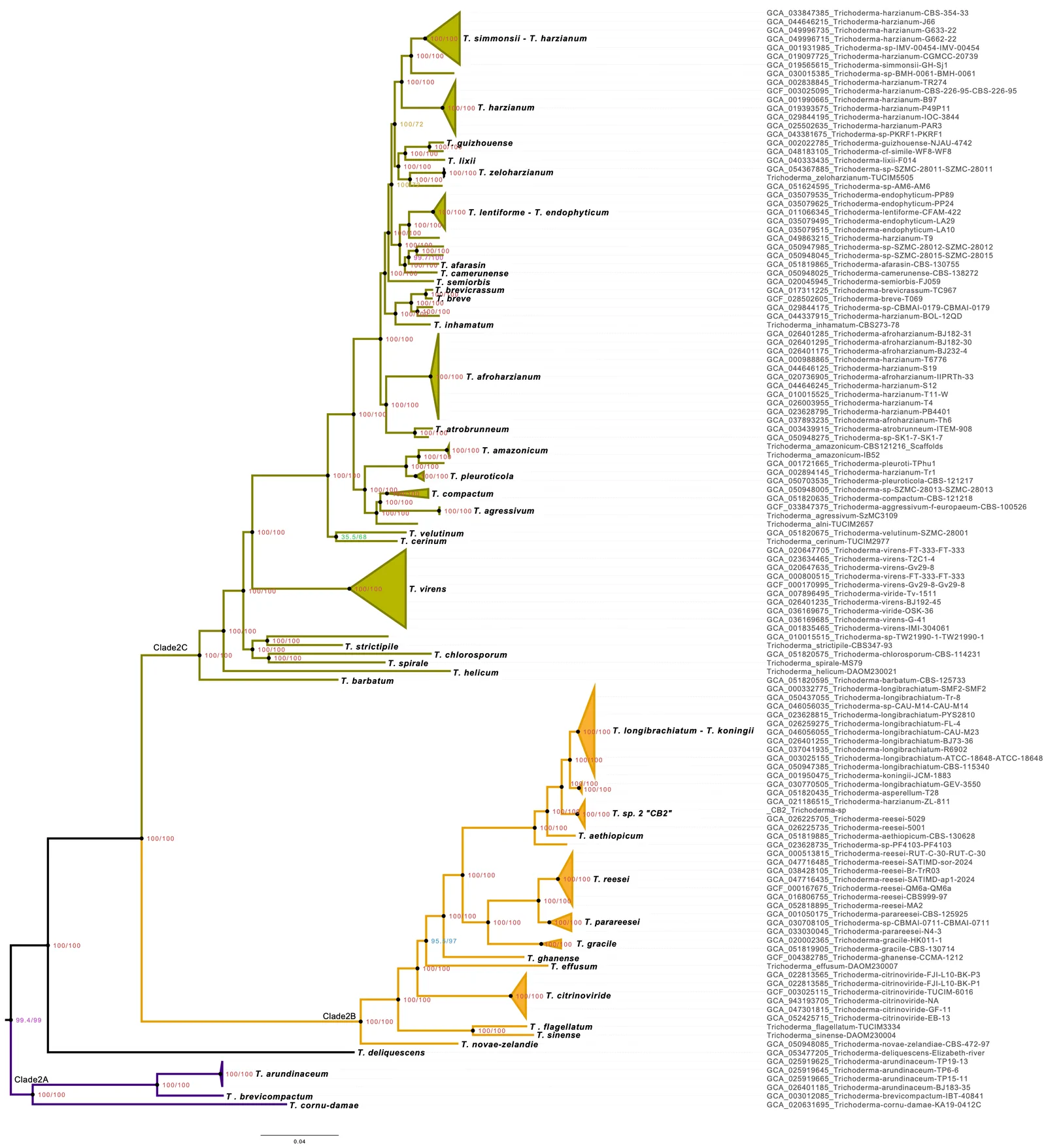
Maximum-likelihood phylogenomic tree corresponding to *Trichoderma* Clade 2, reconstructed using IQ-TREE from the concatenated alignment of 920 conserved CDS partitions. This subtree was extracted from the global phylogenomic reconstruction shown in Figure 1 and encompasses the major lineages designated as Clade 2A, Clade 2B, and Clade 2C. Branch support values correspond to SH-aLRT/UFBoot support obtained from 2000 SH-aLRT replicates and 5000 ultrafast bootstrap replicates and are indicated at each node. Species-level lineages are represented as collapsed cartoon clades to facilitate visualization of the broader phylogenetic relationships. The phylogenetic tree was visualized and rendered using FigTree v1.4.4 (http://tree.bio.ed.ac.uk/software/figtree/, accessed on 7 November 2025).

Phylogenomic analysis of *Trichoderma* Clade 2 revealed a complex evolutionary architecture encompassing several major lineages and species complexes, including the Harzianum, Virens, Longibrachiatum, Reesei–Parareesei, and Citrinoviride complexes. Overall, the global topology was reconstructed with robust statistical support, with most species-level lineages receiving maximal SH-aLRT and ultrafast bootstrap (UFBoot) values (100/100). Despite this broad stability, several localized regions of phylogenetic uncertainty were identified. In particular, *T. lentiforme* was reconstructed as nested within the *T. endophyticum* cluster, rendering the latter paraphyletic and challenging the reciprocal monophyly of these two sister taxa based on current genomic assemblies. Similarly, the *T. velutinum–T. cerinum* lineage displayed reduced nodal support (35.5/68) (Figure 3).

Clade 2 was anchored by a highly supported basal lineage (99.4/99) comprising *T. cornu-damae*, which formed a sister group to a subclade containing *T. arundinaceum* and *T. brevicompactum* (hereafter designated Clade 2A). A second major assemblage, designated Clade 2B, encompassed the Longibrachiatum, Reesei–Parareesei, and Citrinoviride complexes, alongside several allied lineages. Within this assemblage, the Reesei–Parareesei complex was resolved into two well-supported monophyletic groups corresponding to *T. reesei* and *T. parareesei*. Notably, our analysis clarified the taxonomic placement of *Trichoderma* sp. CBMAI-0711, nesting it cleanly within the *T. parareesei* lineage. Although the overall topology of the *T. reesei* clade remained stable, a single internal node exhibited lower support (25.4/56), indicating limited local phylogenetic resolution among these closely related strains. Finally, *T. gracile* was positioned as a distinct sister lineage to the broader Reesei–Parareesei complex.

A highly divergent and supported lineage was also identified, comprising the Colombian isolate CB2 clustered together with three inconsistently annotated public accessions: *T. harzianum* ZL-811 (GCA_021186515) and the *T. reesei* strains 5029 and 5001 (GCA_026225705 and GCA_026225735, respectively). This distinct lineage formed a sister group to the Longibrachiatum complex and was clearly separated from the core *T. reesei*, *T. parareesei*, *T. gracile*, and *T. citrinoviride* reference clades. The deep phylogenetic divergence observed, combined with the conflicting historical taxonomic annotations within this cohort, suggests that these strains may constitute a previously unrecognized candidate species lineage, provisionally designated here as *Trichoderma* sp. 2 “CB2”, an observation that warrants future formal polyphasic evaluation.

The Citrinoviride species complex was recovered as a highly coherent monophyletic group with maximal support across all internal nodes. All analyzed accessions clustered according to their expected species assignments, indicating a comparatively stable taxonomic structure relative to other *Trichoderma* complexes evaluated in this study.

In contrast, the *T. longibrachiatum* species lineage revealed several prominent cases of taxonomic incongruence. Accession GCA_051820435 (strain T28), currently annotated as *T. asperellum*—a member of a distinct section of the genus—was deeply nested within the *T. longibrachiatum* clade. Likewise, *T. koningii* JCM-1883 (GCA_001950475) clustered within the core *T. longibrachiatum* lineage and exhibited minimal phylogenetic divergence relative to the reference strain CBS 115340.

Finally, a large assemblage designated Clade 2C constituted a well-supported cohort encompassing the *Harzianum sensu stricto* (*s. s.*) and *T. virens* complexes, though several localized regions of phylogenetic instability and taxonomic incongruence were detected. Within the *Harzianum* complex, our phylogenomic reconstruction confirmed *T. afroharzianum* as a robustly supported monophyletic lineage. Multiple genomes currently cataloged under the broad umbrella name *T. harzianum*—including strains S12, S19, T4, T11, and PB4401—clustered unequivocally within the *T. afroharzianum* lineage.

In contrast, *T. harzianum* (*s. s.*) formed a distinct and supported lineage containing the critical neotype strain CBS 226.95, alongside reference strains TR274 and B97. This lineage was further subdivided into two moderately differentiated sublineages: one containing the primary neotype strain and another encompassing accessions CBS 354.33 and CGMCC 20739. Notably, the reference genome of *T. simmonsii* (GH-Sj1) was nested within this latter *T. harzianum* sublineage, resulting in a lack of reciprocal monophyly between these two recognized sister taxa at the whole-genome scale. This topology suggests highly permeable or unresolved species boundaries and highlights the clear need for a targeted polyphasic reassessment within the Harzianum–Simmonsii complex.

Additional extensive taxonomic inconsistencies were identified among other genomes sharing the *T. harzianum* label. Accession T9 (GCA_049863215) clustered adjacent to the *T. lentiforme–T. endophyticum* assemblage, whereas accession Tr1 (GCA_002894145) grouped within the *T. pleuroticola* lineage. Similarly, accession BOL-12QD (GCA_044337915) clustered with *Trichoderma* sp. CBMAI-0179 and the *T. breve/T. brevicrassum* lineage rather than within the core Harzianum complex.

The *T. virens* lineage was reconstructed with maximal support but also exhibited evidence of historical nomenclature cross-contamination. Two genomes currently annotated as *T. viride*—strain Tv-1511 (GCA_007896495) and strain OSK-36 (GCA_036169675)—were both robustly nested within the *T. virens* monophyletic lineage.

### 2.3. Geographic Distribution of Genome Accessions Across Major Trichoderma Clades

We evaluated the geographic distribution of the four major phylogenomic clades identified in this study (Clades 1, 2A, 2B, and 2C) using strain provenance information obtained from public repositories, primarily NCBI BioSample records, and complemented with data from the corresponding scientific literature. These data provide a database-record distribution of the sampled genomes rather than an estimate of the actual geographic ranges of the clades. The observed patterns should therefore be interpreted in the context of potential repository, taxonomic, geographic, and research-intensity biases associated with opportunistic genome sampling. Within these limitations, the available records show representatives of the genus across Africa, Asia, Europe, North America, South America, and Oceania, with no records identified from Antarctica.

At the clade level, Clades 1, 2B, and 2C exhibited broad geographic distributions spanning multiple continents. Clade 1 was the most widely distributed lineage, being detected in countries from all inhabited continents, including China, Turkey, Egypt, Italy, Poland, Costa Rica, the United States, Brazil, Australia, South Africa, and New Zealand, among others. Similarly, Clade 2C showed an extensive global distribution, with records from Europe, Asia, Africa, Oceania, and the Americas, including Denmark, Spain, Russia, India, Australia, Zimbabwe, Brazil, Argentina, Peru, and the United States. Clade 2B also displayed a broad intercontinental distribution, occurring in countries such as Colombia, Brazil, Mexico, China, Turkey, Ethiopia, Malaysia, Australia, Argentina, and the United Kingdom.

**Figure 4:**
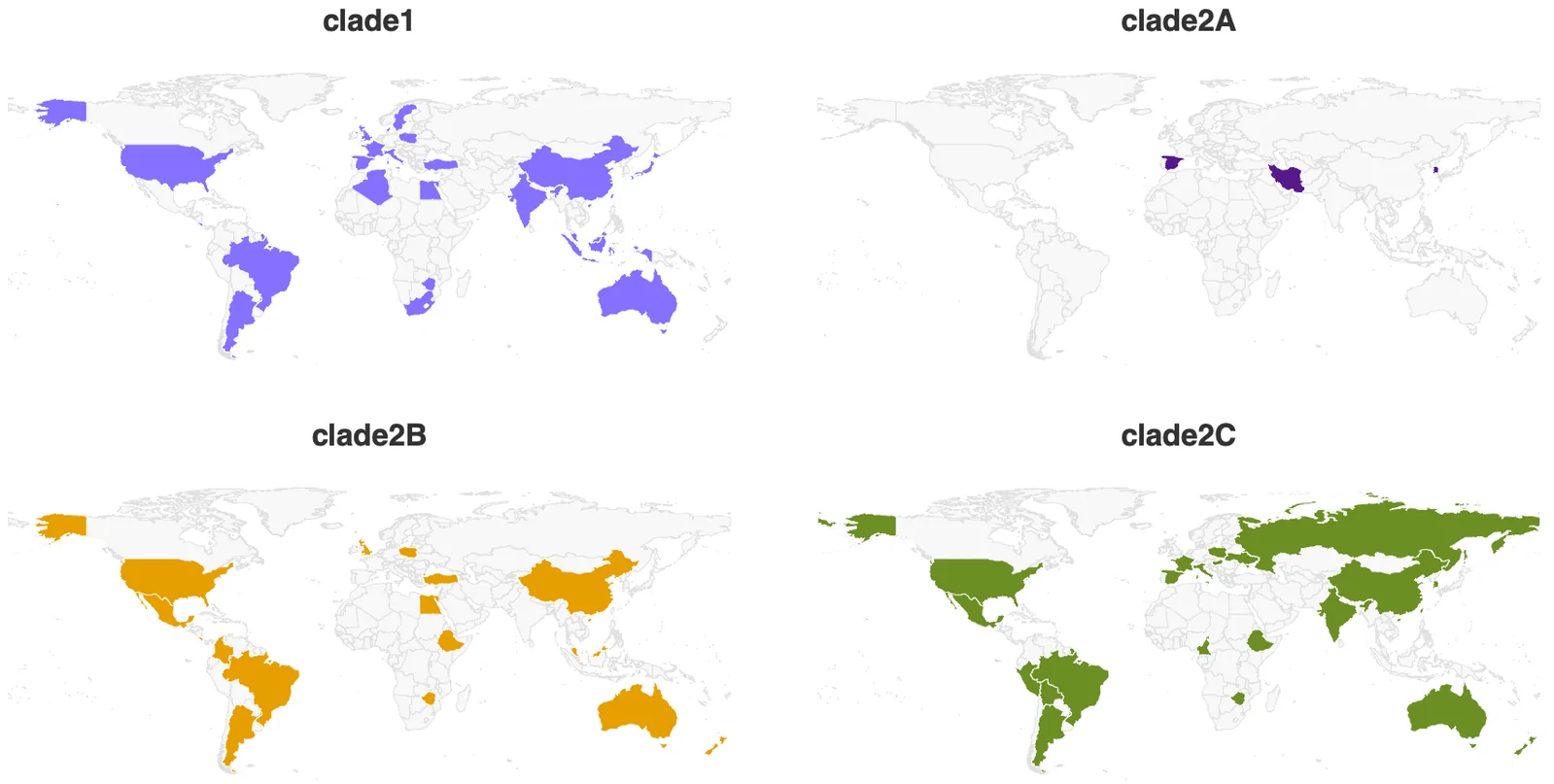
Global Geographic Origins of Genome Accessions and Distribution of the Four Major *Trichoderma* Clades. Geographic distribution maps are faceted independently for Clade 1 (lavender), Clade 2A (dark purple), Clade 2B (orange), and Clade 2C (olive drab). Filled colored regions indicate countries with at least one confirmed genome isolation event belonging to that specific clade. Light gray regions represent countries where the respective clade has not been recorded or where geographical metadata was unavailable. Strains lacking geographical provenance in public repositories or associated literature were excluded from spatial mapping. Spatial visualization and map plotting were performed in R (v4.3.2) using the ggplot2 sf, and rnaturalearth packages. Country vector boundaries were retrieved from Natural Earth (https://www.naturalearthdata.com/, accessed 8 May 2026). Genome provenance and geographical metadata were extracted from NCBI GenBank assembly records.

In contrast, Clade 2A exhibited a markedly more restricted geographic distribution. Representatives of this lineage were identified only in Europe and Asia, with genomes originating from Spain, Iran, and South Korea. However, the apparent restricted distribution of Clade 2A should be interpreted cautiously because this lineage was represented by a substantially smaller number of genomes than the other major clades. Consequently, its limited geographic range may partially reflect undersampling rather than a true ecological or biogeographic restriction (Figure 4).

### 2.4. Distinct Genomic Profiles and Feature Correlations Across Trichoderma Clades

To investigate genomic diversification within the genus *Trichoderma* while controlling for assembly quality and species-level pseudoreplication (across 46 species), we evaluated genome assembly size and GC content across the four major phylogenomic clades. For each trait, we fitted a linear mixed-effects model with clade as the primary predictor, controlling for assembly N50 ($\log_{10}N50$) and BUSCO completeness as fixed covariates, with species identity as a random intercept (lme4::lmer). Overall clade significance was evaluated via Type III ANOVA (lmerTest), followed by post hoc Tukey pairwise comparisons of estimated marginal means (emmeans).

**Figure 5:**
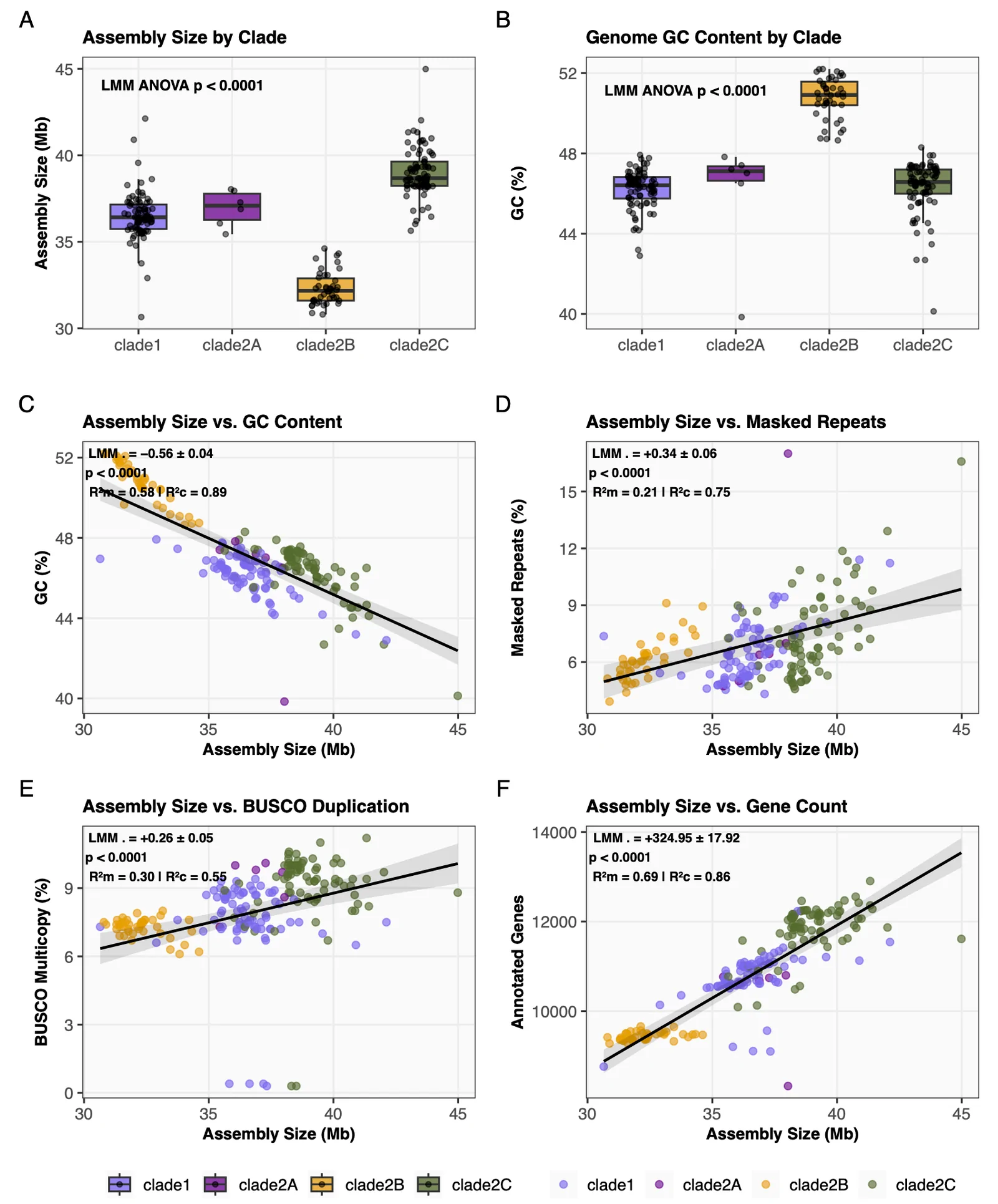
Comparative genomic trait distributions and multivariable linear mixed-effects modeling across *Trichoderma* genomes. (**A**,**B**) Distribution of genome assembly size (Mb) (**A**) and GC content (%) (**B**) across major phylogenomic clades (Clade 1, Clade 2A, Clade 2B, and Clade 2C). Boxplots indicate the median (center line) and interquartile range (IQR; box bounds), whiskers extend to 1.5×IQR, and jittered points represent individual genome assemblies. Annotations display Type III ANOVA p-values derived from linear mixed-effects models (LMMs; lme4::lmer), controlling for assembly contiguity ($\log_{10}N50$), BUSCO completeness percentage, and species-level random intercepts (1|Species). (**C**–**F**) Multivariable LMM relationships between total assembly size (Mb) and genomic features: GC content (%) (**C**), proportion of masked repetitive regions (%) (**D**), percentage of multicopy BUSCO genes (**E**), and annotated gene count (**F**). Solid black lines and shaded ribbons denote model-derived population-level marginal predictions and 95% confidence intervals, respectively (ggeffects), computed while holding technical covariates ($\log_{10}N50$ and BUSCO completeness) constant and accounting for species-level random effects. Inset text reports fixed-effect slope estimates (β±SE), two-tailed p-values, and Nakagawa’s marginal ($R_{m}^{2}$) and conditional ($R_{c}^{2}$) coefficients of determination (performance::r2_nakagawa). Points are color-coded by clade assignment. All statistical modeling, calculations, and figure panels were generated in R (v4.3.2) using lme4, lmerTest, ggeffects, performance, ggplot2, and patchwork packages.

Genome assembly size varied significantly across clades ($p=1.29\times 10^{-26}$, p<0.0001). Post hoc comparisons revealed that Clade 2B possessed a significantly reduced estimated mean genome size (32.5±0.39 Mb) relative to Clade 1 (37.4±0.31 Mb, adjusted p<0.0001), Clade 2A (37.7±0.87 Mb, adjusted p<0.0001), and Clade 2C (39.3±0.29 Mb, adjusted p<0.0001). Furthermore, genomes in Clade 2C were significantly larger than those in Clade 1 (adjusted p<0.05), whereas Clade 2A did not differ significantly from either Clade 1 or Clade 2C (both adjusted p>0.05) (Figure 5A).

Clade assignment also significantly predicted genome GC content ($p=2.44\times 10^{-22}$, p<0.0001). Clade 2B displayed a significantly higher estimated mean GC content (53.2%±0.26%) compared to Clade 1 (48.5%±0.21%, adjusted p<0.0001), Clade 2A (47.9%±0.57%, adjusted p<0.0001), and Clade 2C (48.7%±0.19%, adjusted p<0.0001). No statistically significant differences in GC content were detected among Clades 1, 2A, and 2C (all adjusted p>0.05) (Figure 5B).

To further evaluate genomic patterns across *Trichoderma*, we assessed the relationship between genome assembly size and several genomic features—including GC content, repetitive element content, BUSCO multicopy genes, and the number of predicted genes—using multivariable linear mixed-effects models that controlled for assembly contiguity ($\log_{10}N50$), BUSCO completeness, and species-level random effects. Multivariable LMMs revealed significant associations across all evaluated variables. Genome assembly size showed a strong negative association with GC content (β=−0.56%±0.04% per Mb, $\;p=1.53\times 10^{-36}$; $\;R_{m}^{2}=0.58$, $\;R_{c}^{2}=0.89$). In contrast, genome size was positively associated with the proportion of repetitive elements (β=+0.34%±0.06% per Mb, $\;p=1.22\times 10^{-8}$; $\;R_{m}^{2}=0.21$, $\;R_{c}^{2}=0.75$), the percentage of BUSCO multicopy genes (β=+0.26%±0.05% per Mb, $\;p=4.40\times 10^{-7}$; $\;R_{m}^{2}=0.30$, $\;R_{c}^{2}=0.55$), and the number of predicted genes (β=+324.95±17.92 genes/Mb, $p=6.01\times 10^{-41}$; $\;R_{m}^{2}=0.69$, $\;R_{c}^{2}=0.86$). Among these variables, gene count displayed the strongest response to assembly size expansion, confirming that genome expansion in *Trichoderma* is associated with both increased coding capacity and repetitive DNA accumulation (Figure 5C–F).

### 2.5. Genome Conservation Within and Between Major Trichoderma Clades

**Figure 6:**
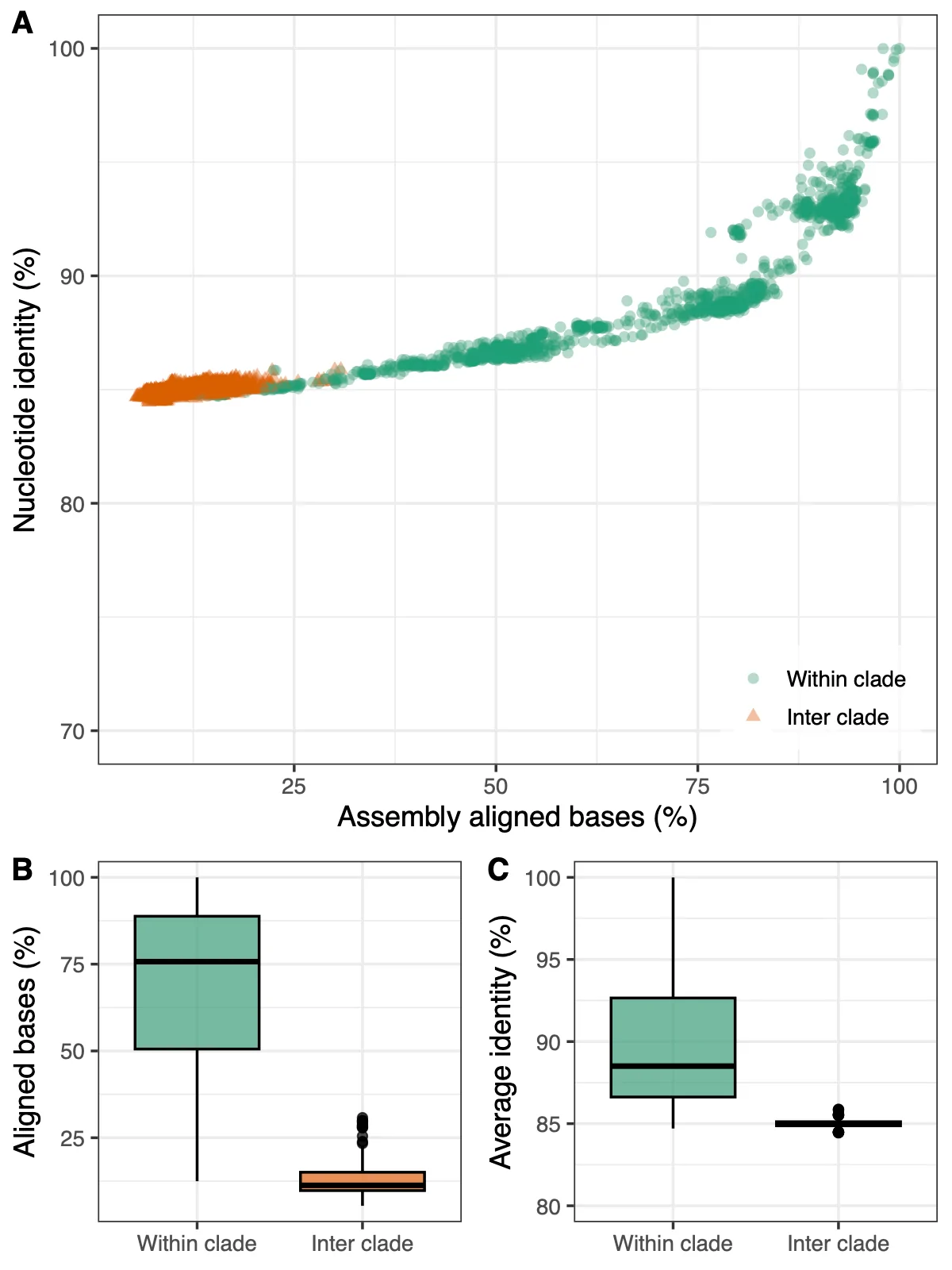
Genome conservation among *Trichoderma* genomes based on pairwise whole-genome alignments. (**A**) Scatterplot showing the relationship between assembly aligned bases (%) and average nucleotide identity (%) for all pairwise genome comparisons. Points are colored and shaped according to whether the comparison was performed between genomes belonging to the same clade (within clade) or different clades (inter clade). (**B**) Distribution of assembly aligned bases (%) for within-clade and inter-clade genome comparisons. (**C**) Distribution of average nucleotide identity (%) for within-clade and inter-clade genome comparisons. Boxplots display the median, interquartile range, and 1.5× interquartile range whiskers. Comparisons within clades generally exhibited higher nucleotide identity and a greater proportion of aligned bases than comparisons between clades, reflecting increased genomic conservation among members of the same phylogenetic group.

To evaluate the degree of genomic conservation across the genus, pairwise whole-genome alignments were performed among all analyzed assemblies. A strong contrast was observed between intra-clade and inter-clade genome conservation patterns. Comparisons between genomes belonging to the same major phylogenomic clade showed substantially higher proportions of aligned genomic regions, with aligned bases ranging from 12.5% to nearly complete genome (>98%), and a median aligned fraction of 75.7%. In contrast, comparisons between genomes from different major clades exhibited markedly reduced genomic conservation, with aligned fractions ranging from only 5.4% to 30.8% and a median of 11.3%. Similarly, nucleotide identity among aligned regions differed between intra- and inter-clade comparisons. Within-clade genome alignments displayed average nucleotide identities ranging from 84.7% to 99.9%, with a median identity of 88.5%. In contrast, inter-clade comparisons showed a narrower and slightly lower identity range, varying from 84.4% to 85.9%, with a median of 85.0%. However, these inter-clade identity values should be interpreted cautiously, as only a small fraction of the genomes could be aligned between distantly related clades. Consequently, the calculated nucleotide identity reflects only the most conserved genomic regions and likely overestimates the true overall genomic conservation among the major *Trichoderma* lineages (Figure 6).

**Figure 7:**
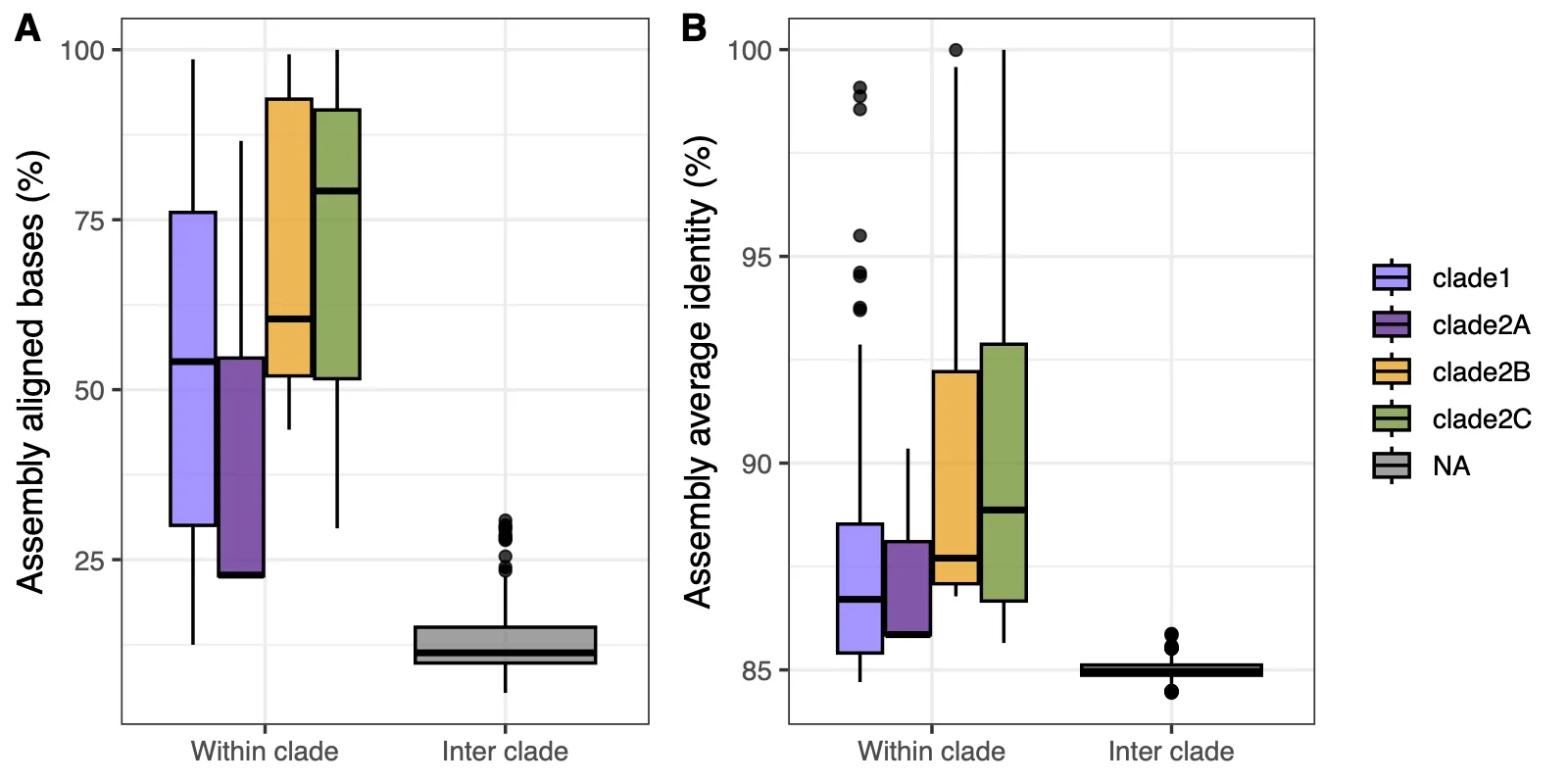
Pairwise genome conservation comparisons among the four main *Trichoderma* clades. (**A**) Distribution of assembly aligned bases (%) between genome pairs classified as within-clade or inter-clade comparisons. (**B**) Distribution of average nucleotide identity (%) across the same comparison groups. Boxplots are colored according to the clade assignment of each genome pair: Clade 1 (lavender), Clade 2A (purple), Clade 2B (orange), Clade 2C (olive green). Boxes represent the interquartile range (IQR), center lines indicate the median, and whiskers represent data dispersion beyond the quartiles.

When genome conservation was evaluated independently within each major phylogenomic clade, substantial differences in genomic cohesion were observed. Clade 2C exhibited the highest degree of genome conservation, with a median aligned genomic fraction of 79.2%. Concordantly, this clade also showed the highest median nucleotide identity among aligned regions (88.9%). Clade 2B displayed the second-highest internal conservation, with a median aligned genomic fraction of 60.4% and a median nucleotide identity of 87.7%. Clade 1 exhibited a lower genomic conservation, with a median aligned genomic fraction of 54.1% and a median nucleotide identity of 86.7%. Clade 2A, although represented by a limited number of pairwise comparisons, showed the lowest genomic conservation, with a median aligned fraction of 22.7% and a median nucleotide identity of 85.8% (Figure 7). All statistical calculations and figure panels were generated in R (v4.3.2) using ggplot2 packages.

### 2.6. Trichoderma Core Proteome

To explore the proteome divergence among the major *Trichoderma* clades, we performed an orthology-based analysis to identify core proteome and main clade-specific proteins, defined as orthologous groups present in at least 80% of genomes within a given clade and completely absent from genomes outside that lineage. The core proteome was composed of 3491 orthologous protein groups. Functional annotation using eggNOG-mapper yielded a total of 1761 unique KEGG Orthology (KO) identifiers. Subsequent metabolic pathway reconstruction using KEGGDecoder revealed a highly robust and streamlined metabolic architecture, with several key pathways displaying maximum (100%) completeness across the core genome dataset. Characterization of these fully conserved pathways highlighted three primary functional thematic groups. (1) Carbohydrate-active machinery and mycoparasitic determinants: The core proteome maintained complete (100%) enzymatic cascades for starch and glycogen degradation, including alpha-amylase, glucoamylase, and beta-glucosidase. Crucially, chitinase activity was resolved at 100% completeness across all core assemblies, reinforcing the preservation of chitinolytic cell-wall degrading machinery as a foundational ancestral trait within the *Trichoderma* lineage. (2) Amino acid anabolism and nutrient assimilation: The core proteome exhibited complete metabolic self-sufficiency (prototrophy) for key structural and signaling amino acids. Biosynthetic pathways for serine, threonine, glutamine, glycine, lysine, aspartate, glutamate, and the aromatic amino acids phenylalanine and tyrosine all achieved 100% validation scores. This anabolic capacity was supported by a fully conserved sulfur assimilation pathway, indicating a stable infrastructure for organic sulfur processing. (3) Energy conservation and homeostasis: In terms of central carbon catabolism and organic acid management, pathways governing mixed-acid fermentation—specifically acetate production and the conversion of ethanol/acetate to acetaldehyde—were entirely complete. Furthermore, cellular homeostasis and metal detoxification mechanisms were anchored by the absolute conservation of the copper transporter CopA.

Collectively, these KEGG profile findings indicate that while accessory genomes drive niche-specific adaptations and secondary metabolite specialization, the *Trichoderma* core proteome preserves a highly efficient, rigid metabolic foundation optimized for aggressive carbohydrate breakdown, basic amino acid biosynthesis, and fundamental environmental survival mechanisms.

### 2.7. Proteome Divergence in Secondary Metabolism Repertoires Among Trichoderma Main Clades

**Figure 8:**
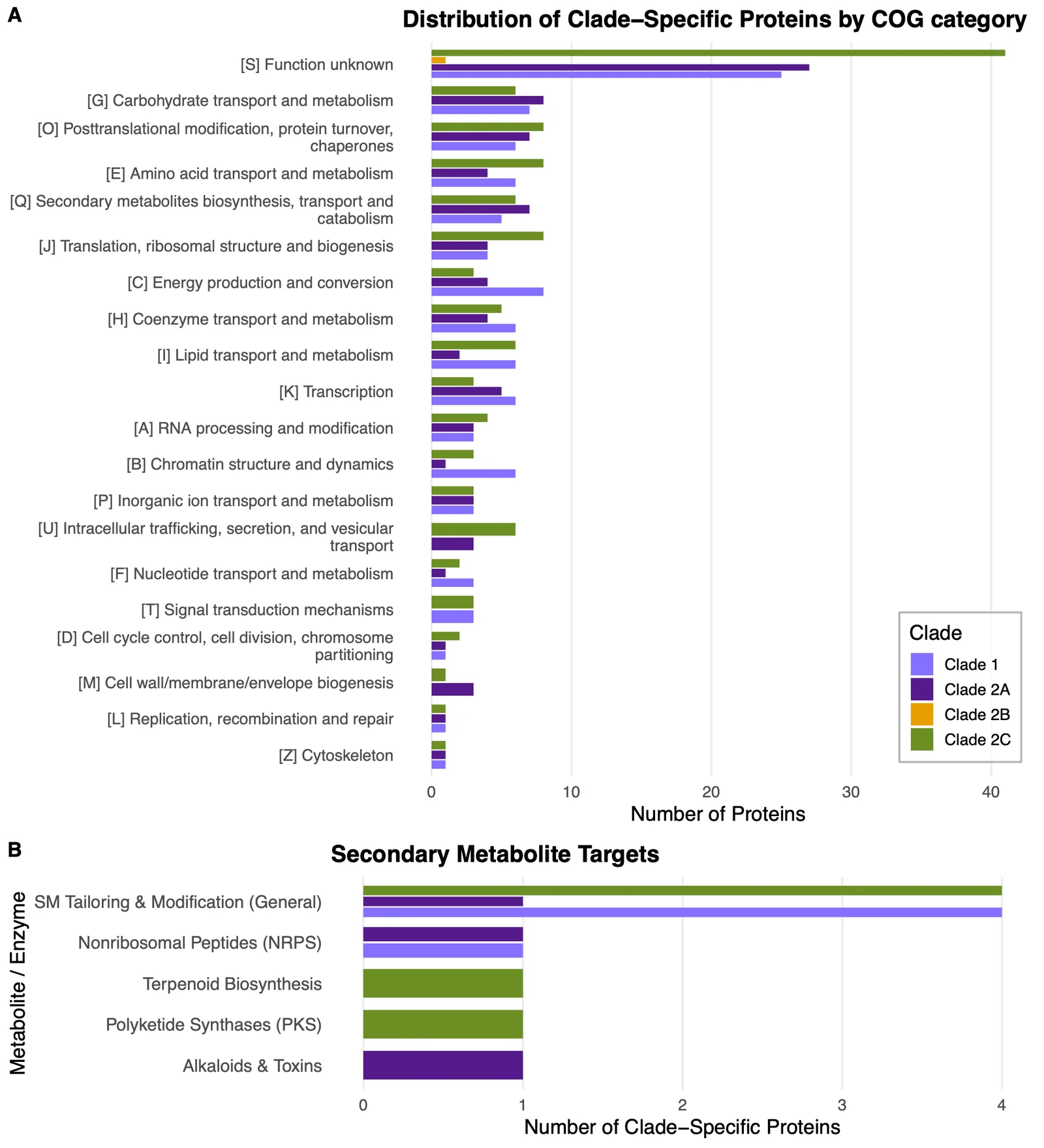
(**A**) Functional classification of clade-specific proteins identified in *Trichoderma* genomes based on Clusters of Orthologous Groups (COG) categories. Horizontal bar plots show the most abundant functional categories detected among clade-specific proteins from the Clade 1, Clade 2A, Clade 2B, and Clade 2C genomes. Bars represent the number of proteins assigned to each COG category, with colors indicating the corresponding clade. Functional assignments include categories associated with metabolism, transcription, posttranslational modification, signal transduction, intracellular trafficking, and secondary metabolite biosynthesis. COG category descriptions were derived from EggNOG-mapper annotations. Clade 2B is included in the legend but lacks visible bars because no proteins from this clade received functional assignments under the EggNOG-mapper annotation strategy. (**B**) Distribution of putative secondary metabolite-associated proteins identified among clade-specific proteins from *Trichoderma* genomes. Horizontal bar plots summarize proteins associated with biosynthetic and tailoring functions related to secondary metabolism, including polyketide synthases (PKSs), nonribosomal peptide synthetases (NRPSs), terpene biosynthesis, siderophore-associated proteins, cytochrome P450 enzymes, alkaloid and toxin-related proteins, and general secondary metabolite tailoring enzymes. Functional categories were inferred through text mining of EggNOG-mapper annotations and PFAM domain descriptions. Bars represent the number of proteins assigned to each functional category, with colors corresponding to the associated clade. All statistical calculations, and figure panels were generated in R (v4.3.2) using the ggplot2 packages.

To investigate whether the major phylogenomic clades of *Trichoderma* differ not only in genome architecture but also in lineage-specific functional innovation, we compared the repertoires of single-copy clade-specific orthologous proteins and their associated metabolic functions. Despite exhibiting the largest genome assemblies, Clade 2C did not show the highest number of clade-specific proteins, with only 23 unique proteins identified. In contrast, Clade 2A contained the largest repertoire of clade-specific proteins (n = 81), followed by Clade 1 (n = 26). Clade 2B, which exhibited the smallest genome assemblies, contained only 14 clade-specific proteins, none of which retrieved functional annotations from the KO, COG, or GO databases. COG functional classification of clade-specific proteins in Clades 1, 2A, and 2C revealed that successfully annotated sequences were predominantly associated with metabolic processes, spanning both primary and secondary metabolism. However, the global functional annotation rate across these lineage-exclusive proteins was low, leaving the majority uncharacterized or assigned to unknown functions. (Figure 8A).

To further investigate the evolutionary divergence of secondary metabolism (SM) capabilities across the analyzed lineages, clade-specific protein sets were screened for functional domains associated with the biosynthesis of antifungal compounds, including polyketides, non-ribosomal peptides, terpenoids, and alkaloids. Overall, the number of strictly clade-specific SM proteins was relatively low, suggesting that much of the secondary metabolism repertoire is conserved across the genus or shared among multiple clades. Among the identified targets, enzymes involved in SM tailoring and modification were the most frequently detected, indicating that lineage-specific diversification is likely driven more by modifications of existing metabolite scaffolds than by the acquisition of entirely novel biosynthetic backbone enzymes (Figure 8B).

Distinct patterns of SM-associated enrichment were observed among clades. Clade 2A displayed the highest diversity of unique SM-related proteins and was the only lineage containing clade-specific proteins associated with alkaloid and toxin biosynthesis (n = 2). This clade additionally encoded one unique non-ribosomal peptide synthetase (NRPS) and two SM tailoring enzymes. Clades 1 and 2C were both primarily characterized by unique SM tailoring enzymes (n = 3 each). However, Clade 1 additionally contained one lineage-specific NRPS, whereas Clade 2C uniquely encoded a protein associated with terpenoid biosynthesis (n = 1). In contrast, Clade 2B lacked clade-specific proteins across all evaluated secondary metabolism categories, indicating that its predicted functional repertoire may depend on alternative metabolic pathways or uncharacterized gene sets rather than annotated secondary metabolite gene clusters.

### 2.8. Phylogenomic Distinction and Cohesion of the Putative Trichoderma sp. 2 “CB2” Lineage

**Figure 9:**
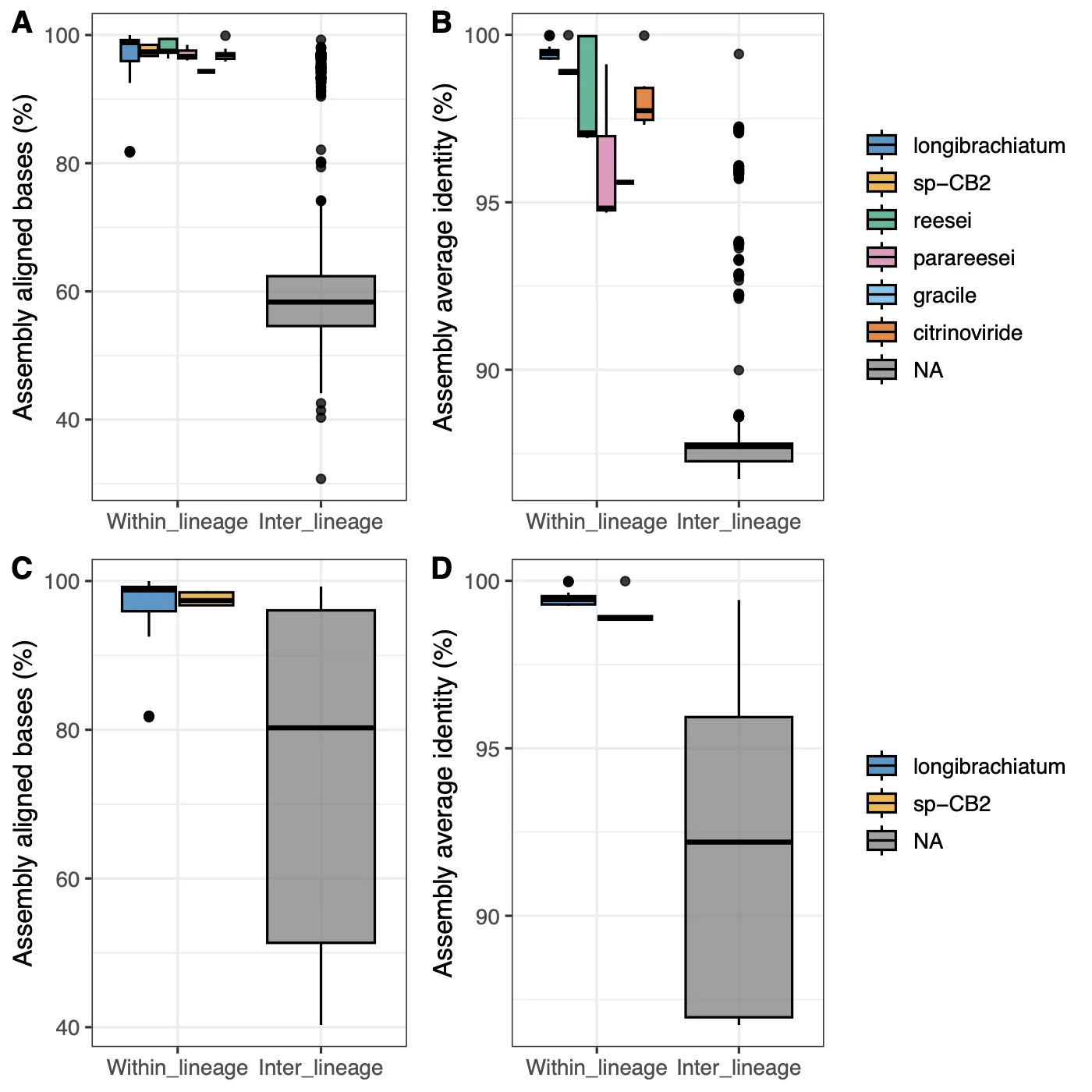
Pairwise genome conservation comparisons among *Trichoderma* Clade 2B assemblies. (**A**,**B**) Distributions of assembly aligned bases (%) (**A**) and average nucleotide identity (%) (**B**) among genome pairs within Clade 2B, categorized by comparison type (within-lineage vs. inter-lineage). Colors indicate the lineage assignment of the compared genomes (*Trichoderma longibrachiatum*, candidate species lineage *Trichoderma* sp. 2 “CB2”, *Trichoderma reesei*, *Trichoderma parareesei*, *Trichoderma gracile*, and *Trichoderma citrinoviride*). (**C**,**D**) Target pairwise genome conservation metrics comparing candidate species lineage *Trichoderma* sp. 2 “CB2” and *Trichoderma longibrachiatum* assemblies, showing assembly aligned bases (%) (**C**) and average nucleotide identity (%) (**D**) across within-lineage and inter-lineage combinations. Boxplots represent non-parametric summary statistics (median and interquartile range). All statistical calculations, and figure panels were generated in R (v4.3.2) using the ggplot2 packages.

We sought to quantitatively evaluate the genome-wide divergence of the highly distinct lineage identified in this study, which comprises the Colombian isolate CB2 clustered alongside three inconsistently annotated public accessions: *T. harzianum* ZL-811 (GCA_021186515) and *T. reesei* strains 5029 and 5001 (GCA_026225705 and GCA_026225735, respectively). Within our global phylogenomic reconstruction, this cohort formed a robustly supported monophyletic sister group to the Longibrachiatum species complex, positioning itself entirely outside the core *T. reesei*, *T. parareesei*, and *Citrinoviride* reference clades. To test whether this topological divergence reflects true species-level isolation, we performed comprehensive pairwise whole-genome comparisons across the principal lineages of Clade 2B. The resulting taxogenomic metrics revealed a stark, unambiguous discontinuity between intra-lineage cohesion and inter-lineage divergence. (i) Broad Clade 2B inter-lineage baseline: Pairwise comparisons among all major established lineages within Clade 2B yielded a global median aligned genomic fraction of 58.3% and a median nucleotide identity of 87.7%, illustrating the high baseline genomic diversity characterizing this clade. (ii) Intra-lineage cohesion: In sharp contrast, genomes compared within their respective assigned lineages displayed profound conservation, with aligned genomic fractions consistently spanning 94.3% to 98.8% and median nucleotide identities exceeding 94.0% across all configurations (Figure 9A,B).

A focused, high-resolution comparison between the *T. longibrachiatum sensu stricto* clade and the *Trichoderma* sp. 2 “CB2” cohort further substantiated this pattern of genetic segregation. Internal genomic consistency within both groups was exceptionally high; the *T. longibrachiatum* lineage exhibited a median aligned genomic fraction of 98.8% with a median nucleotide identity of 99.4%, while the *Trichoderma* sp. 2 “CB2” lineage maintained a median aligned fraction of 97.4% and a median nucleotide identity of 98.9%. Conversely, cross-comparison metrics between *T. longibrachiatum* and *Trichoderma* sp. 2 “CB2” dropped precipitously. The median aligned genomic fraction fell to 80.2%, and the cross-lineage median nucleotide identity decreased markedly to 92.2%. In line with comparative genomic benchmarks for fungal species boundaries (~95–96% average identity), these alignment metrics—when combined with high intra-lineage sequence cohesion and deep phylogenomic divergence—indicate that the ‘CB2’ cohort represents a distinct and evolutionarily independent candidate species warranting formal polyphasic description (Figure 9C,D).

### 2.9. Clade-Specific Functional Signatures of the Trichoderma sp. 2 “CB2” Lineage

To characterize the genomic features distinguishing the candidate *Trichoderma* sp. 2 “CB2” lineage from other members of Clade 2B and from the broader *Trichoderma* dataset, we searched for orthologous proteins conserved across the four genomes representing this lineage but absent from the remaining comparative dataset. A total of 49 lineage-associated proteins, ranging from 67 to 494 amino acids in length, were identified. Functional annotation yielded recognizable domains or database matches for only six proteins, whereas the remaining 43 proteins lacked detectable PFAM, COG, KO, GO, or EggNOG annotations, indicating that their functions remain unresolved. The annotated subset comprised proteins putatively associated with transcriptional regulation, plant cell-wall degradation, secondary metabolism, and metabolic detoxification.

The lineage-specific proteome resolved into four major functional categories. First, proteins associated with transcriptional regulation and genomic specialization were identified, including a lineage-specific Zn(II)2Cys6 fungal transcription factor containing a canonical GAL4-like zinc cluster DNA-binding domain (PFAM: Zn_clus). In filamentous fungi, these transcription factors are frequently associated with the regulation of specialized metabolic pathways and cryptic secondary metabolite biosynthetic gene clusters. This regulatory component was accompanied by a putative secretory pathway protein carrying a DUF1242 domain, suggesting potential adaptations in intracellular trafficking and protein sorting.

A second category comprised proteins potentially associated with plant biomass degradation and carbohydrate metabolism. These included a pectate lyase superfamily protein belonging to the PL3 family (PFAM: Pectate_lyase_3), which is associated with the degradation of pectic components of plant cell walls. A lineage-associated nucleoside-diphosphate-sugar epimerase involved in nucleotide-sugar interconversion was also identified. The presence of these proteins in the four genomes of the candidate lineage is consistent with potential differences in carbohydrate metabolism and plant biomass utilization, although their specific ecological roles cannot be inferred from sequence annotation alone.

The third functional category involved secondary metabolism and xenobiotic processing. A lineage-specific cytochrome P450 monooxygenase (PFAM: p450) was identified and assigned to COG categories associated with lipid metabolism and secondary metabolite biosynthesis (categories I and Q). Cytochrome P450 enzymes commonly function as tailoring enzymes within fungal biosynthetic gene clusters, contributing to the structural diversification of bioactive secondary metabolites. The exclusive presence of this enzyme in the CB2 lineage suggests lineage-specific metabolic capabilities potentially associated with ecological competition or chemical defense.

Finally, the lineage-associated proteome included an aldehyde dehydrogenase (ALDH) family protein (PFAM: Aldedh), assigned to COG category E (amino acid transport and metabolism). The predicted protein was associated with NAD+- and NAD(P)+-dependent aldehyde dehydrogenase activities (EC 1.2.1.3 and EC 1.2.1.5) and mapped to multiple KEGG pathways, including glycolysis/gluconeogenesis, pyruvate metabolism, fatty acid degradation, amino acid catabolism, and xenobiotic degradation. These annotations indicate a potentially broad metabolic role in aldehyde conversion and cellular metabolism; however, the precise contribution of this protein to the biology of the candidate CB2 lineage remains to be experimentally established.

## 3. Discussion

By deploying a curated dataset of 920 single-copy orthologous loci, this study resolved a robust and highly supported phylogenomic backbone for the genus *Trichoderma*, defining two principal evolutionary radiations further subdivided into four major clades (Clades 1, 2A, 2B, and 2C). This genome-scale topology largely corroborates the terminal clade structures established by legacy multilocus sequence typing (MLST) frameworks, while providing stronger statistical support for several previously ambiguous deep relationships. Consistent with single- and few-locus phylogenies, the major industrially relevant species complexes were recovered as monophyletic. Clade 1 primarily comprised representatives of the Viride species complex while also including taxa such as T. minutisporum. The Brevicompactum and Longibrachiatum species complexes corresponded to Clades 2A and 2B, respectively. Within Clade 2C, the *Trichoderma harzianum* species complex and the *T. virens* species complex were recovered as strongly supported sister lineages, closely mirroring terminal topologies inferred from tef1-α and rpb2 barcoding [19]. Although the genome-scale approach provides greater resolution and accommodates incomplete lineage sorting (ILS), the exact order of the deepest divergences may still be influenced by taxon sampling and the evolutionary models applied. Therefore, the four-clade genomic framework presented here should be regarded as a robust and highly resolved evolutionary hypothesis that complements, rather than replaces, the foundational systematic frameworks of *Trichoderma*.

Beyond recovering a broader evolutionary structure of the genus, the phylogenomic framework also exposed widespread taxonomic incongruences across several species complexes. Importantly, the genome-scale approach allowed these inconsistencies to be resolved with strong statistical support while simultaneously highlighting the extent of misannotations currently present in public repositories such as NCBI. This issue is particularly relevant because NCBI constitutes one of the primary global references for fungal genome data used by researchers, industry, and regulatory agencies, all of which frequently rely on deposited taxonomic assignments for downstream analyses, strain validation, and biotechnology applications [4].

One of the clearest examples involved the *T. asperellum–T. asperelloides* complex, where multiple genomes currently annotated as *T. asperellum* were robustly nested within the *T. asperelloides* lineage. This pattern highlights the recurrent limitations of legacy morphology-based classifications in resolving closely related sister taxa within *Trichoderma*. Similarly, within Clade 2C, strains broadly labeled as *T. harzianum* were distributed across multiple independent lineages, including *T. afroharzianum* and *T. pleuroticola*. Furthermore, the absence of reciprocal monophyly between *T. harzianum sensu stricto* and *T. simmonsii* at the whole-genome scale suggests the existence of an ongoing evolutionary radiation within this complex. Such phylogenomic patterns may reflect incomplete lineage sorting, historical hybridization events, or highly permeable species boundaries that are difficult to resolve using traditional multilocus taxonomic approaches alone [11].

This genome-scale framework not only recovered the macro-topology of historically recognized species complexes but also revealed clear large-scale patterns of genome evolution across the genus. Notably, the major clades exhibited contrasting genomic architectures. Clade 2B contained the most compact genomes within the genus, whereas Clade 2C displayed the largest assemblies. In Clade 2B, genome reduction was accompanied by significantly higher GC content, suggesting contraction through the loss of AT-rich genomic regions. In contrast, larger assembly sizes in Clade 2C were positively associated with predicted gene counts, higher proportions of multicopy BUSCO markers, and elevated repetitive element content—genomic features that correlate with assembly size variation across clades. Across the genus, genome size showed moderate positive correlations with predicted gene content, multicopy BUSCO percentages, and repetitive element abundance, whereas a strong negative correlation was observed between genome size and GC content. Collectively, these results indicate that the major *Trichoderma* clades have followed distinct trajectories of genome evolution involving differential patterns of genome expansion, contraction, duplication, and repetitive sequence dynamics.

Interestingly, genome expansion in Clade 2C was not accompanied by the acquisition of a proportionally larger repertoire of lineage-specific proteins, as this clade contained only 23 clade-specific single copy orthologous protein groups. In contrast, Clade 2A, despite exhibiting intermediate genome sizes, possessed the highest number of clade-specific single-copy orthologous groups in the genus, with 81 lineage-specific protein families identified. This disparity suggests that genome expansion in Clade 2C is driven primarily by internal genomic duplication processes and repetitive sequence accumulation rather than by extensive acquisition of novel functional gene repertoires. Conversely, the elevated number of lineage-specific proteins in Clade 2A represents predicted genomic capacity that may point to candidate pathways for functional diversification, despite maintaining comparatively moderate genome sizes.

Functional profiling of the *Trichoderma* core proteome, including both single-copy and multicopy orthologous groups, uncovered a remarkably rigid metabolic foundation. The absolute (100%) conservation of primary amino acid biosynthetic cascades, sulfur assimilation pathways, and central carbohydrate-active machinery indicates that the fundamental saprotrophic lifestyle of the genus is highly optimized and evolutionarily fixed. Most notably, the universal conservation of complete chitinolytic pathways is consistent with the hypothesis that cell wall degradation and mycoparasitism are ancestral, foundational traits of the genus rather than localized, lineage-derived innovations.

In contrast, predicted genomic capacity associated with potential niche-specific traits is concentrated primarily within the flexible accessory and lineage-specific proteomes. Our secondary metabolism (SM) analysis revealed that the primary antifungal and defensive arsenals of *Trichoderma* are anchored by stable, ancestral core biosynthetic backbones (such as polyketide synthases—PKSs and non-ribosomal peptide synthetases—NRPSs). Cladal diversification occurs primarily through variations in auxiliary tailoring enzymes (such as cytochromes P450 and monooxygenases) [20] and localized regulatory fine-tuning. For example, the unique enrichment of alkaloid and toxin tailoring components in Clade 2A represents predicted metabolic capacity for specialized secondary metabolism [21], whereas candidate noncanonical genes and potential regulatory variation in Clade 2B highlight priority targets for future functional characterization.

The identification of the lineage *Trichoderma* sp. 2 “CB2” provides additional evidence of hidden phylogenomic diversity within Clade 2B. This cohort forms a supported monophyletic sister lineage to the Longibrachiatum complex, yet exhibits substantial whole-genome divergence relative to neighboring species groups. Although genomic cohesion within the “CB2” lineage remained exceptionally high (>98% nucleotide identity), pairwise comparisons against *T. longibrachiatum* showed a marked reduction in nucleotide identity, reaching a median value of 92.2%. While whole-genome identity metrics serve as comparative heuristics rather than rigid species boundaries, the observed inter-lineage divergence—when combined with high internal sequence cohesion and deep phylogenomic isolation—supports the ‘CB2’ lineage as a distinct candidate species requiring future formal polyphasic evaluation [22,23].

Functional characterization of the lineage-specific proteome further supports the distinctiveness of the “CB2” cohort. Of the 49 proteins identified as exclusive to this lineage, 43 lacked detectable annotations in current databases, suggesting the presence of a substantial fraction of highly divergent or still uncharacterized proteins. The remaining annotated proteins revealed a coordinated set of functions potentially associated with regulatory specialization, plant biomass degradation, secondary metabolism, and metabolic detoxification. Among these, the presence of a lineage-specific Zn(II)2Cys6 transcription factor suggests potential specialization in the regulation of secondary metabolism or niche-associated pathways [24,25]. Likewise, the identification of a PL3-family pectate lyase and a nucleoside-diphosphate-sugar epimerase indicates possible adaptations related to cell wall degradation and carbohydrate processing [26,27,28]. In addition, the exclusive cytochrome P450 monooxygenase identified in this lineage may contribute to specialized secondary metabolite tailoring or xenobiotic processing [20], while the multifunctional aldehyde dehydrogenase points to broad metabolic flexibility linked to central carbon metabolism and detoxification pathways [29].

Taken together, these genomic and functional patterns suggest that the “CB2” lineage possesses a combination of evolutionary and metabolic characteristics distinct from neighboring lineages within Clade 2B. While additional ecological, morphological, and reproductive evidence will be necessary to formally resolve its taxonomic status, these results illustrate how integrated phylogenomics and comparative functional analyses can help uncover previously unrecognized diversity within *Trichoderma* and refine the evolutionary framework of this economically and ecologically important fungal genus.

Nevertheless, the inherent limitations of computational annotation should be acknowledged. Whole-genome predictions, pangenomic classifications, and secondary-metabolite pathway reconstructions indicate potential genomic capacity rather than direct evidence of functional activity. Therefore, the biological and ecological relevance of the predicted traits associated with the “CB2” lineage requires experimental validation. Future studies integrating transcriptomic analyses under contrasting environmental conditions, targeted mutagenesis or gene-knockout assays, biochemical characterization of enzymatic products, and broad phenotypic profiling will be essential to confirm gene expression, validate functional outputs, and determine how these genomic features influence ecological performance in natural environments.

In routine diagnostic workflows, legacy molecular markers such as ITS, tef1α, and rpb2 remain practical, low-cost tools for rapid preliminary screening and the initial assignment of isolates to broad species complexes. However, because single- or few-locus markers frequently lack sufficient resolving power to disentangle closely related lineages—and often perpetuate long-standing taxonomic ambiguities—definitive species-level assignment can only come from genome-scale analyses. Moving forward, genome-wide phylogenomics, backed by concordant concatenated and coalescent frameworks, must be adopted as the essential standard for resolving complex lineages, evaluating candidate species, and validating the identity of industrial or ecologically regulated *Trichoderma* strains. Crucially, this genome-first baseline is fully compatible with—and complements—the phenotypic, physiological, and morphological profiling required for formal species descriptions, seamlessly aligning genome-scale resolution with the polyphasic criteria mandated by the International Code of Nomenclature for algae, fungi, and plants (ICN) and the International Commission on the Taxonomy of Fungi (ICTF).

## 4. Materials and Methods

### 4.1. Genomic Data Collection and Sequencing of the Colombian Trichoderma Isolate

All *Trichoderma* genomes available in the NCBI Datasets repository were systematically collected beginning in July 2023, with the dataset updated approximately every six months to incorporate newly released assemblies. The final update was performed in January 2026, yielding 236 genome assemblies. Mutant derivatives were identified from accession metadata and excluded from downstream analyses. Potentially redundant genomes were further evaluated using whole-genome pairwise comparisons with the MUMmer dnadiff function. Two assemblies were considered redundant when >98% of their genome was aligned with >99.99% nucleotide identity. In cases where highly similar assemblies represented the same or equivalent strain, the assembly with the higher N50 value, provided that the overall assembly size was comparable, was retained. Assemblies failing the predefined quality criteria after CAT-based contamination filtering (BUSCO completeness <80%, N50 ≤ 40,000 bp, or ≥2000 scaffolds) were also excluded from downstream analyses. When both GCA and GCF accessions were available for the same strain, the RefSeq (GCF) assembly was preferentially selected. We kept 191 of them. Species identities and reference lineages were validated through consultation of the MycoBank database (https://www.mycobank.org, accessed on 8 May 2026), confirming that the corresponding NCBI accessions were associated with validly recognized taxa and reference strains whenever possible. Multiple validated reference genomes were included for several species to improve lineage consistency and phylogenetic robustness. NCBI genome accessions used in this study as well as general genomic statistics, are described in Supplementary Table S2.

Additionally, 25 genomes corresponding to *Trichoderma* species not yet available in the NCBI repository at the time of analysis were incorporated from the study of Steindorff et al. [14]. After filtering, 16 were retained for further analysis. These assemblies were downloaded from the JGI MycoCosm portal (https://mycocosm.jgi.doe.gov, accessed on 8 May 2026) and included the following strains: *Trichoderma minutisporum DAOM175931*, *T. strigosellum IQ191*, *T. taiwanense IQ11*, *T. effusum DAOM230007*, *T. flagellatum TUCIM3334*, *T. sinense DAOM230004*, *T. zeloharzianum TUCIM5505*, *T. inhamatum CBS273-78*, *T. amazonicum CBS121216*, *T. amazonicum IB52*, *T. aggressivum SzMC3109*, *T. alni TUCIM2657*, *T. cerinum TUCIM2977*, *T. strictipile CBS347-93*, *T. spirale MS79*, and *T. helicum DAOM230021*.

Overall, the sequential retrieval and curation described above resulted in a final dataset of 208 non-redundant *Trichoderma* genomes, comprising genomes retained from NCBI (n = 191), the newly sequenced Colombian isolate *Trichoderma* sp. 2 “CB2” (n = 1), and additional non-redundant genomes obtained from JGI (n = 16). This curated dataset was used for all downstream comparative genomic and phylogenomic analyses.

A novel Colombian *Trichoderma* isolate, designated strain CB2, was recovered from soil collected in the Urabá region of the department of Antioquia, northwestern Colombia, in April 2023. Isolation was performed using a serial dilution–plating method on acidified potato dextrose agar (PDA) supplemented with chloramphenicol to selectively inhibit bacterial growth. Genomic DNA was extracted using the QIAGEN DNeasy PowerSoil Kit and sequenced using Illumina NovaSeq technology (Illumina, San Diego, CA, USA), generating paired-end reads of 150 bp. Raw reads were quality-filtered and adapter-trimmed using Cutadapt v3.5 [30], retaining reads with a minimum Phred quality score of 30 and a minimum length of 70 bp. Then, de novo genome assembly was performed using SPAdes v3.13.1 [31] with the –careful and –cov-cutoff auto options enabled and testing of k-mer sizes 55, 77, and 99. To assess assembly support and sequencing coverage, quality-filtered paired-end reads were mapped to the final assembly using Bowtie2, and the resulting BAM file was evaluated using SAMtools, v1.14. The final filtered assembly was 31.3 Mb and comprised 233 scaffolds, with an N50 of 610,626 bp. Mapping resulted in 95.25% of reads aligned to the assembly, with a mean genome-wide depth of 85.61×. Coverage was highly uniform, with 100%, 99.78%, 99.70%, and 99.55% of the assembly covered at ≥1×, ≥10×, ≥20×, and ≥30×, respectively. Contaminant contigs were identified using the Contig Annotation Tool (CAT) [32] and then removed from the assembly. Isolate CB2 genomic raw reads were deposited at the SRA database under bioproject PRJNA1512456, and the complete genome assembly, before removal of contaminating contigs, was deposited at DDBJ/ENA/GenBank under the accession JCCJVH000000000. The version described in this paper is version JCCJVH010000000.

### 4.2. Phylogenomic Inference

For phylogenomic reconstruction, conserved single-copy orthologous genes were identified among the analyzed *Trichoderma* genomes using BUSCO v5.2.2 [33] with the sordariomycetes_odb10 lineage dataset and the parameter AUGUSTUS species = chaetomium_globosum. BUSCO-derived single-copy proteomes were subsequently processed with SonicParanoid v1.3.5 [34] to perform an additional orthology validation step and exclude potential paralogous loci. After curation, a final dataset comprising 920 non-redundant single-copy CDSs was retained for phylogenomic analysis.

Each orthologous CDS was individually aligned using MAFFT v7.490, without using a codon-aware alignment approach [35]. Individual alignments were subsequently concatenated into a partitioned supermatrix (comprising 435 gene partitions and a total of 3,895,880 nucleotide sites) using CatSequences (https://github.com/ChrisCreevey/catsequences, accessed on 12 December 2026). Maximum-likelihood phylogenetic inference was performed with IQ-TREE v3 [36] using a partitioned approach, with the best-fit substitution model for each partition selected independently using ModelFinder [37]. To reduce partition complexity while preserving model fit, the -rcluster 10 option was enabled. Branch support was evaluated using 2000 SH-aLRT replicates and 5000 ultrafast bootstrap pseudoreplicates [38].

The final phylogeny was rooted using *Trichoderma orchidacearum* (GCA_023653515). Tree visualization and annotation were performed using FigTree v1.4.4 (https://tree.bio.ed.ac.uk/software/figtree/, accessed on 12 December 2026), and final figure editing was completed in Adobe Illustrator, v28.

To independently validate the root placement and deep evolutionary topology of the genus-wide *Trichoderma* phylogeny, an outgroup-rooted phylogenomic analysis was conducted across 59 genomes. The dataset comprised 57 representative *Trichoderma* genomes and two outgroup species: Hypomyces perniciosus HP10 (GenBank accession: GCA_008477525) and Hypomyces rosellus CCMJ2808 (GenBank accession: GCA_011799845). Due to low nucleotide sequence conservation across these divergent genera, analysis was performed on amino acid sequences rather than coding sequences (CDS). BUSCO-predicted proteomes were processed with SonicParanoid to identify single-copy orthologous protein loci conserved in at least 80% of the sampled genomes. A concatenated alignment of 3577 single-copy protein loci was subjected to maximum likelihood (ML) phylogenetic inference in IQ-TREE under the LG4M amino acid substitution model with 5000 ultrafast bootstrap (UFB) replicates. The resulting outgroup-rooted tree confirmed that Hypomyces forms a fully supported external clade (UFB = 100) and recovered *Trichoderma orchidacearum* as the earliest-diverging lineage relative to all remaining *Trichoderma* species with maximal support (UFB = 100), maintaining the major deep evolutionary structure of the genus (Supplementary Figure S4).

To account for potential gene tree discordance and incomplete lineage sorting (ILS), a coalescence-based species tree was inferred alongside the concatenated phylogenomic analysis. First, individual unrooted maximum likelihood gene trees were estimated for each of the 920 single-copy CDS alignments using IQ-TREE3. For each locus, the best-fit substitution model was automatically selected using ModelFinder Plus (-m MFP). The resulting 920 gene trees were concatenated into a single file and analyzed using the multi-threaded ASTRAL implementation within the ASTER C++ package (astral-cpp) [39] to infer the final species tree topology. Branch support was evaluated using local posterior probabilities (LPP), which assess confidence based on quartet frequencies and are highly robust to gene tree conflict.

### 4.3. Strain Metadata and Geographic Distribution Analysis

To explore the geographic distribution of the major *Trichoderma* phylogenomic clades, strain provenance metadata were compiled for all genomes included in this study. Country of origin information was retrieved from the NCBI BioSample database and complemented, when necessary, through consultation of the original publications describing the strains or genome assemblies. Genomes lacking reliable geographic information were excluded from the spatial analyses. Country names were standardized according to ISO 3166-1 [40] conventions to ensure consistency during downstream mapping procedures.

Geographic visualization was performed in R using the tidyverse, sf, rnaturalearth, and ggplot2 packages. World map geometries were obtained from the rnaturalearth database and converted into simple feature (sf) objects for spatial plotting. Antarctica was excluded from the final map because no strains included in this study originated from this region.

To minimize biases associated with uneven sampling intensity among countries, the dataset was transformed into a presence/absence matrix, recording only whether a given clade had been detected in each country regardless of the number of genomes available. Spatial distributions were then visualized independently for each major phylogenomic clade using faceted maps generated with *ggplot2*, allowing for a direct comparison of their global geographic ranges.

### 4.4. Genome Repetitive Elements Masking and Annotation

Prior to genome annotation, repetitive elements were identified and masked using RepeatMasker v4 in combination with a custom repeat library constructed with RepeatModeler v2.0.7) [41,42]. The repeat library was assembled by downloading fungal repeat sequences from the Dfam database (dfam_fungi.fa, accessed January 2026) and generating a de novo repeat library from the reference genome *Trichoderma atroviride* P1 (accession GCA_020647795) using RepeatModeler. Both libraries were subsequently concatenated into a combined fungal repeat library (combined_fungal_library.fasta). Soft-masking of all genome assemblies was performed using RepeatMasker with the combined library, generating GFF-format annotation outputs (RepeatMasker flags: -lib combined_fungal_library.fasta -pa 32 -gff -xsmall). Following repeat masking, potential contaminating sequences were detected and removed using CAT_pack v6.0.1. Each genome assembly was classified at the contig level against the CAT NR database (version 20241212_CAT_nr_website, downloaded January 2026), and taxonomic names were assigned to all classified contigs using the CAT_pack add_names module. Contigs not classified as belonging to the genus *Trichoderma* were subsequently excluded from further analysis using an in-house Python v3.9.5 script, yielding a set of taxonomically filtered genome assemblies. Structural annotation of the filtered genome assemblies was performed using BRAKER (braker.pl version 3.0.8) [43] in fungal mode, leveraging a curated protein database as evidence. Reference protein sequences were obtained from UniProtKB for the genus *Trichoderma* (CDHIT_uniprotkb_Trichoderma_2026_02_03.faa, accessed February 2026), and gene models were predicted for each assembly using the parameters –fungus –species = *Trichoderma* alongside the soft-masked filtered contigs as genomic input. The completeness of each predicted proteome was assessed using BUSCO v6.0.0 against the Sordariomycetes lineage dataset (sordariomycetes_odb10) in protein mode (-m prot), providing a standardized benchmark of annotation quality across all genomes. Finally, orthologous gene families across all annotated proteomes were inferred using SonicParanoid v1.3.5, with all proteome files supplied as a directory input. The resulting orthogroup assignments formed the basis for subsequent comparative genomic analyses across the sampled *Trichoderma* genomes.

### 4.5. Comparative Genomic and Statistical Modeling

To evaluate large-scale variation in genomic traits while explicitly accounting for species-level pseudoreplication and assembly quality, linear mixed-effects models (LMMs) were implemented using the lme4 package in R v2025.05.0 + 496. All statistical tests were conducted on a curated dataset of 205 genome assemblies representing 46 phylogenomic species distributed across four major clades (Clade 1, 2A, 2B, and 2C). To control for evolutionary non-independence among conspecific genomes, species identity was assigned as a random intercept (1|Species) across all models.

Differences in assembly size (Mb) and GC content (%) across clades were modeled with clade assignment as the primary fixed factor. To isolate biological signals from assembly fragmentation and genome completeness, ${log}_{10}$-transformed N50 ($\log_{10}N50$) and single-copy BUSCO completeness percentages were included as fixed technical covariates (Trait ~ Clade + log_N50 + busco_Complete_perc + (1|Species)). Overall significance of fixed effects was evaluated via ANOVA using Satterthwaite’s approximation for degrees of freedom (lmerTest). Differences among clade estimated marginal means (EMMs) were calculated using post hoc Tukey pairwise adjustments in emmeans.

To examine how assembly size correlated with specific genomic architecture traits—namely GC content, percentage of repeat-masked sequences, BUSCO multicopy percentage, and annotated gene count—multivariable LMMs were constructed with total assembly size (Mb) as the primary fixed predictor while controlling for $\log_{10}N50$, BUSCO completeness, and species-level random intercepts. For each multivariable model, fixed-effect slope estimates (β±SE) and two-tailed p-values were extracted. Model goodness-of-fit was quantified using Nakagawa’s coefficient of determination, calculating marginal $R^{2}$ ($R_{m}^{2}$, variance explained solely by fixed effects) and conditional $R^{2}$ ($R_{c}^{2}$, variance explained by both fixed and random effects combined) using the performance package (r2_nakagawa). Model-based marginal predictions and 95% confidence intervals were calculated using ggeffects for visualization.

### 4.6. Genome-to-Genome Alignment Analysis

Whole-genome pairwise alignments were performed using the DNAdiff pipeline implemented in MUMmer v4 [44] to estimate genome-wide nucleotide identity and the proportion of conserved aligned regions between assemblies. For each pairwise comparison, the percentage of aligned bases and the corresponding average nucleotide identity were extracted and compiled into a comparative matrix. Genome pairs were subsequently classified according to the phylogenomic clade assignments inferred in this study, and redundant reciprocal comparisons were excluded from downstream analyses. To reduce redundancy and avoid overrepresentation of highly similar genomes, very closely related strains within the same species were filtered prior to comparative analyses. When multiple genomes representing the same species were available, priority was given to assemblies corresponding to validated reference strains or accessions recognized in MycoBank whenever possible. This strategy allowed for the retention of representative genomic diversity while minimizing bias caused by the inclusion of numerous nearly identical strains. For each pairwise genome comparison, the percentage of alignable bases (AlignedBases), average sequence identity across alignable regions (AvgIdentity), and total single-nucleotide polymorphisms (TotalSNPs) were extracted and compiled in Supplementary Table S3.

### 4.7. Core Proteome

Orthology inference across the 208 analyzed *Trichoderma* genomes was performed independently using OrthoFinder v2.5.5. Across the full dataset, 22,401 orthogroups were identified, of which 3491 constituted the core proteome present in all 208 genomes (15.6% of total orthogroup diversity). To eliminate redundant database queries across hundreds of highly conserved sequence copies while maintaining standardized functional assignments, a single representative protein sequence per core orthogroup was selected for downstream functional mapping. The proteome of *Trichoderma hamatum* (GCA_000331835) was designated as the primary reference source due to its high assembly completeness and annotation quality. When an orthogroup contained multiple *T. hamatum* sequence copies (paralogs), the longest protein sequence was selected to maximize domain coverage during functional querying. Functional mapping of these representative core sequences against the KEGG database identified a total of 1761 unique KEGG Orthology (KO) entries (Supplementary Table S4).

### 4.8. Clade-Specific Protein Analysis

To minimize interference from multicopy paralogous protein families, the clade-specific protein analysis was restricted to orthologous groups classified as single-copy by SonicParanoid v1.3.5. SonicParanoid was run using the proteomes from all 208 selected genomes, and the resulting single-copy orthologous groups were screened to identify proteins present in at least 80% of the genomes within a given clade and completely absent from genomes outside that lineage.

Clade-specific proteins were identified using a custom Python script designed to detect orthogroups fulfilling these presence/absence criteria across the phylogenomic clades. Functional annotation of predicted proteomes and clade-specific proteins was performed using eggNOG-mapper (EMAPPER) [45].

To evaluate the metabolic and functional capabilities of the *Trichoderma* core proteome, a targeted pathway completeness analysis was performed. Functional annotation of the representative core proteome sequences was initially conducted using eggNOG-mapper to assign KEGG Orthology (KO) identifiers. A custom Python script was utilized to parse the resulting annotations file, filtering out unassigned characters and extracting a non-redundant set of unique KO terms. To meet the precise syntax requirements of downstream profiling tools, these unique identifiers were formatted into a tab-separated vertical layout mapping distinct sequence placeholders to individual KO terms. High-level functional and metabolic module profiling was subsequently executed using KEGG-decoder [46]. Functional pathway completeness values (ranging from 0.0 to 1.0) were calculated across all standard database modules.

### 4.9. Statistical Analyses and Visualization

Statistical analyses of *Trichoderma* genomic features were conducted in R/RStudio v2025.05.0+496 for macOS v2025.05.0 + 496. Data manipulation and summary statistics were performed primarily using the tidyverse package collection [47], while graphical visualization and figure arrangement were generated using ggplot2 [48] and ggpubr. Differences among phylogenomic clades were evaluated using non-parametric Kruskal–Wallis tests due to the non-normal distribution of several genomic variables. Boxplots, scatterplots, and correlation analyses were generated to explore genome structural variation across the analyzed lineages.

## Data Availability

Genomic raw reads for the CB2 isolate were deposited at the SRA database under BioProject “PENDING”.

## Supplementary Materials

The following supporting information can be downloaded at: https://www.mdpi.com/article/10.3390/ijms27188204/s1.
