# Supplementary material for: Phylogenomic and Comparative Genomic Analyses Reveal Deep Evolutionary Structure and Cryptic Diversity in the Genus Trichoderma
Source: Int J Mol Sci. 2026 Sep 15;27(18):8204. doi: 10.3390/ijms27188204 (PMC13607837; doi:10.3390/ijms27188204)
Supplement: Supplementary file 1 [file ijms-27-08204-s001.zip › Supplementary Material Captions.pdf]

Supplemenatry Material.

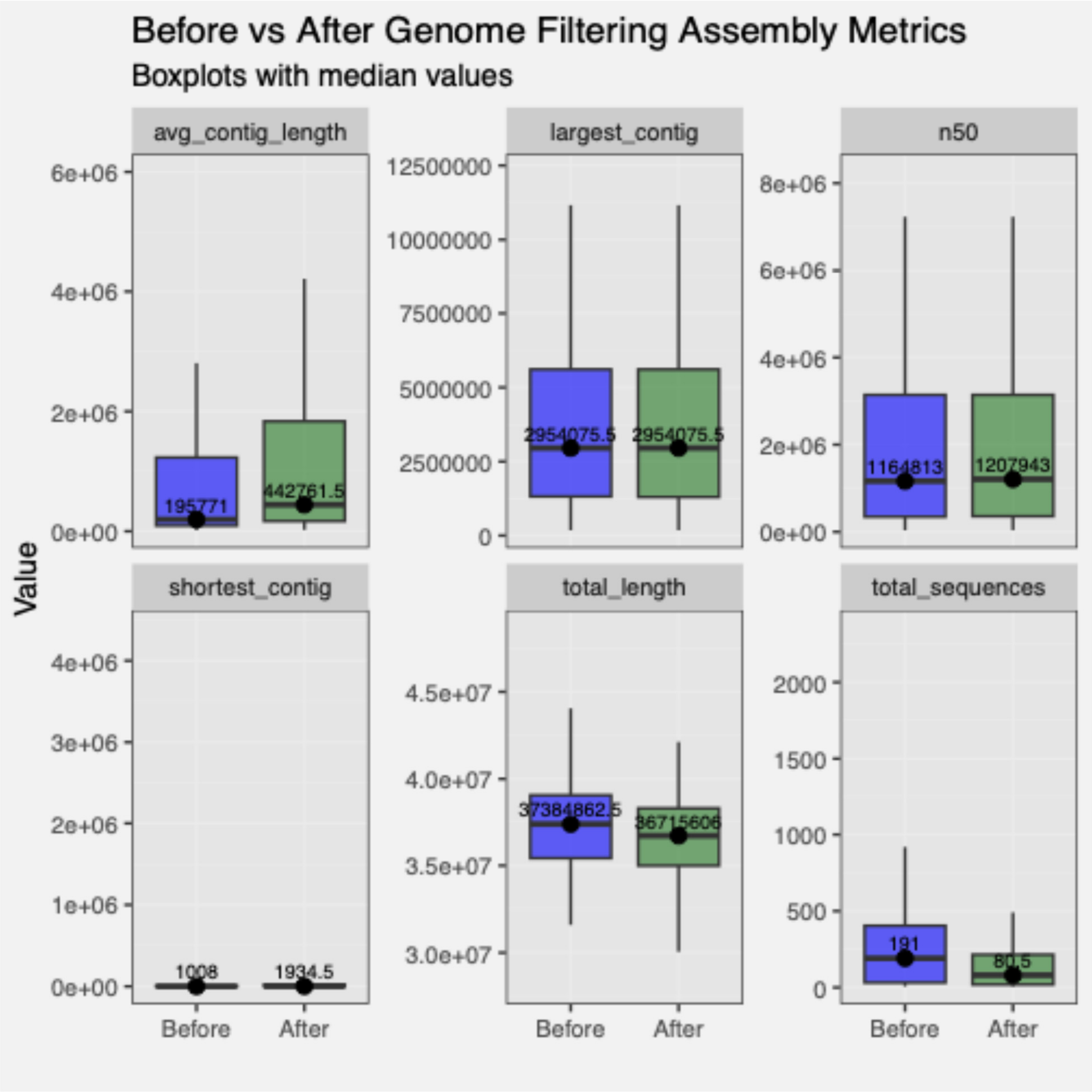

Figure S1. Comparison of genome assembly metrics before and after filtering. Distribution of total assembly size (Mb), contig N50 (kb), contig count, and BUSCO completeness (%) across 208 Trichoderma genome assemblies before and after sequence contamination removal and quality filtering. Boxplots depict the median (center line) and interquartile range (IQR; box bounds), with whiskers extending to 1.5 × IQR and individual assembly

values shown as jittered points. Plots were generated in R (v4.3.2) using the ggplot2 and patchwork packages.

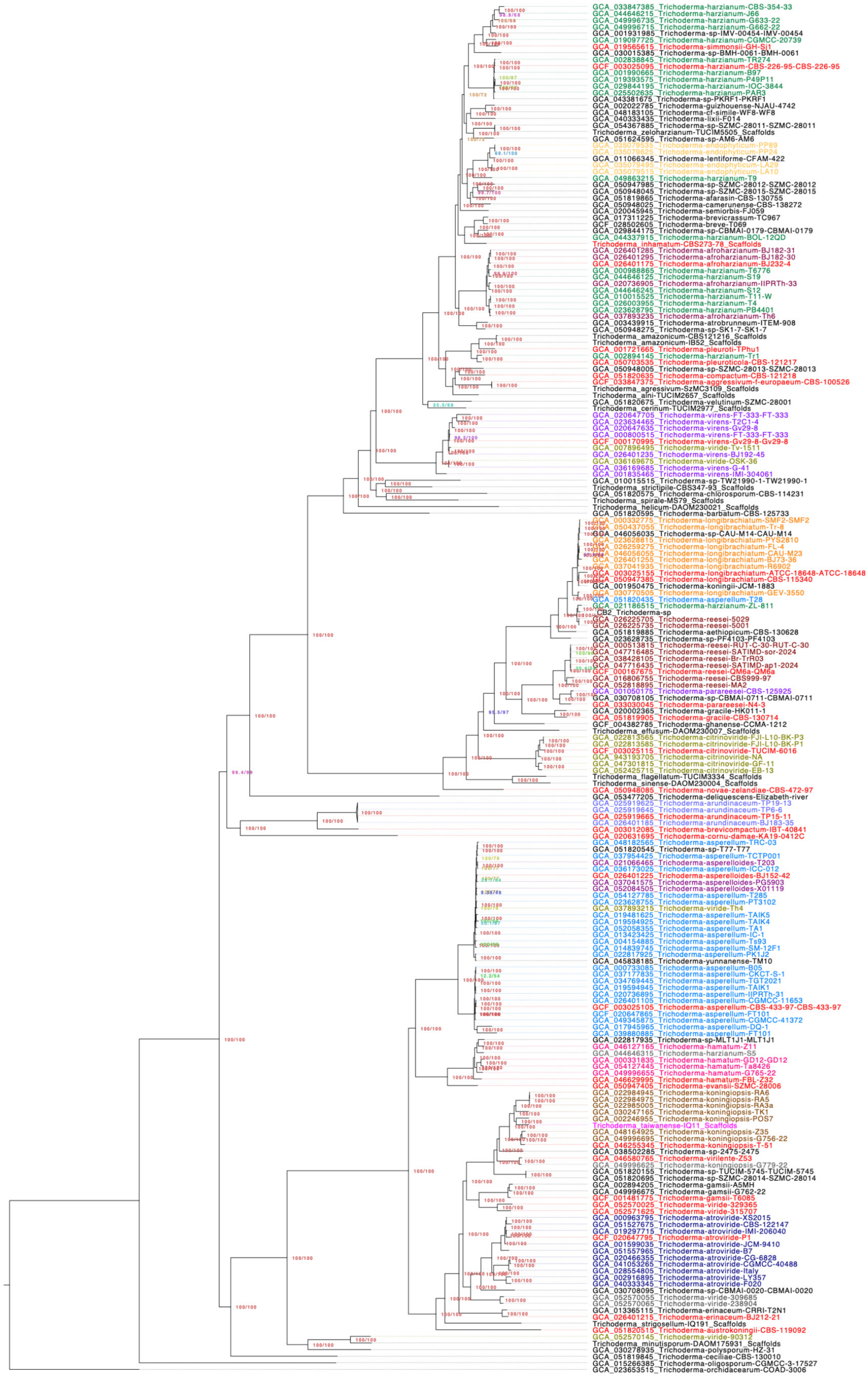
GCA_033847385_Trichoderma-harzianum-CBS-354-33
GCA_044646215_Trichoderma-harzianum-J66
GCA_049996735_Trichoderma-harzianum-G633-22
GCA_049996715_Trichoderma-harzianum-G662-22
GCA_001931985_Trichoderma-sp-IMV-00454-IMV-00454
GCA_019097725_Trichoderma-harzianum-CGMCC-20739
GCA_019565615_Trichoderma-simmonsii-GH-Sj1
GCA_030015385_Trichoderma-sp-BMH-0061-BMH-0061
GCA_002838845_Trichoderma-harzianum-TR274
GCF_003025095_Trichoderma-harzianum-CBS-226-95-CBS-226-95
GCA_001990665_Trichoderma-harzianum-B97
GCA_019393575_Trichoderma-harzianum-P49P11
GCA_029844195_Trichoderma-harzianum-IOC-3844
GCA_025502635_Trichoderma-harzianum-PAR3
GCA_043381675_Trichoderma-sp-PKRF1-PKRF1
GCA_002022785_Trichoderma-guizhouense-NJAU-4742
GCA_048183105_Trichoderma-cf-simile-WF8-WF8
GCA_040333435_Trichoderma-lixii-F014
GCA_054367885_Trichoderma-sp-SZMC-28011-SZMC-28011
Trichoderma_zeloharzianum-TUCIM5505_Scaffolds
GCA_051624595_Trichoderma-sp-AM6-AM6
GCA_035079535_Trichoderma-endophyticum-PP89
GCA_035079625_Trichoderma-endophyticum-PP24
GCA_011066345_Trichoderma-lentiforme-CFAM-422
GCA_035079495_Trichoderma-endophyticum-LA29
GCA_035079515_Trichoderma-endophyticum-LA10
GCA_049863215_Trichoderma-harzianum-T9
GCA_050947985_Trichoderma-sp-SZMC-28012-SZMC-28012
GCA_050948045_Trichoderma-sp-SZMC-28015-SZMC-28015
GCA_051819865_Trichoderma-afarasin-CBS-130755
GCA_050948025_Trichoderma-camerunense-CBS-138272
GCA_020045945_Trichoderma-semiorbis-FJ059
GCA_017311225_Trichoderma-brevicrassum-TC967
GCF_028502605_Trichoderma-breve-T069
GCA_029844175_Trichoderma-sp-CBMAI-0179-CBMAI-0179
GCA_044337915_Trichoderma-harzianum-BOL-12QD
Trichoderma_inhamatum-CBS273-78_Scaffolds
GCA_026401285_Trichoderma-afroharzianum-BJ182-31
GCA_026401295_Trichoderma-afroharzianum-BJ182-30
GCA_026401175_Trichoderma-afroharzianum-BJ232-4
GCA_000988865_Trichoderma-harzianum-T6776
GCA_044646125_Trichoderma-harzianum-S19
GCA_020736905_Trichoderma-afroharzianum-IIPRTh-33
GCA_044646245_Trichoderma-harzianum-S12
GCA_010015525_Trichoderma-harzianum-T11-W
GCA_026003955_Trichoderma-harzianum-T4
GCA_023628795_Trichoderma-harzianum-PB4401
GCA_037893235_Trichoderma-afroharzianum-Th6
GCA_003439915_Trichoderma-atrobrunneum-ITEM-908
GCA_050948275_Trichoderma-sp-SK1-7-SK1-7
Trichoderma_amazonicum-CBS121216_Scaffolds
Trichoderma_amazonicum-IB52_Scaffolds
GCA_001721665_Trichoderma-pleuroti-TPhu1
GCA_002894145_Trichoderma-harzianum-Tr1
GCA_050703535_Trichoderma-pleuroticola-CBS-121217
GCA_050948005_Trichoderma-sp-SZMC-28013-SZMC-28013
GCA_051820635_Trichoderma-compactum-CBS-121218
GCF_033847375_Trichoderma-aggressivum-f-europaeum-CBS-100526
Trichoderma_agressivum-SzMC3109_Scaffolds
Trichoderma_alni-TUCIM2657_Scaffolds
GCA_051820675_Trichoderma-velutinum-SZMC-28001
Trichoderma_cerinum-TUCIM2977_Scaffolds
GCA_020647705_Trichoderma-virens-FT-333-FT-333
GCA_023634465_Trichoderma-virens-T2C1-4
GCA_020647635_Trichoderma-virens-Gv29-8
GCA_000800515_Trichoderma-virens-FT-333-FT-333
GCF_000170995_Trichoderma-virens-Gv29-8-Gv29-8
GCA_007896495_Trichoderma-viride-Tv-1511
GCA_026401235_Trichoderma-virens-BJ192-45
GCA_036169675_Trichoderma-viride-OSK-36
GCA_036169685_Trichoderma-virens-G-41
GCA_001835465_Trichoderma-virens-IMI-304061
GCA_010015515_Trichoderma-sp-TW21990-1-TW21990-1
Trichoderma_strictipile-CBS347-93_Scaffolds
GCA_051820575_Trichoderma-chlorosporum-CBS-114231
Trichoderma_spirale-MS79_Scaffolds
Trichoderma_helicum-DAOM230021_Scaffolds
GCA_051820595_Trichoderma-barbatum-CBS-125733
GCA_000332775_Trichoderma-longibrachiatum-SMF2-SMF2
GCA_050437055_Trichoderma-longibrachiatum-Tr-8
GCA_046056035_Trichoderma-sp-CAU-M14-CAU-M14
GCA_023628815_Trichoderma-longibrachiatum-PYS2810
GCA_026259275_Trichoderma-longibrachiatum-FL-4
GCA_046056055_Trichoderma-longibrachiatum-CAU-M23
GCA_026401255_Trichoderma-longibrachiatum-BJ73-36
GCA_037041935_Trichoderma-longibrachiatum-R6902
GCA_003025155_Trichoderma-longibrachiatum-ATCC-18648-ATCC-18648
GCA_050947385_Trichoderma-longibrachiatum-CBS-115340
GCA_001950475_Trichoderma-koningii-JCM-1883
GCA_030770505_Trichoderma-longibrachiatum-GEV-3550
GCA_051820435_Trichoderma-asperellum-T28
GCA_021186515_Trichoderma-harzianum-ZL-811
CB2_Trichoderma-sp
GCA_026225705_Trichoderma-reesei-5029
GCA_026225735_Trichoderma-reesei-5001
GCA_051819885_Trichoderma-aethiopicum-CBS-130628
GCA_023628735_Trichoderma-sp-PF4103-PF4103
GCA_000513815_Trichoderma-reesei-RUT-C-30-RUT-C-30
GCA_047716485_Trichoderma-reesei-SATIMD-sor-2024
GCA_038428105_Trichoderma-reesei-Br-TrR03
GCA_047716435_Trichoderma-reesei-SATIMD-ap1-2024
GCF_000167675_Trichoderma-reesei-QM6a-QM6a
GCA_016806755_Trichoderma-reesei-CBS999-97
GCA_052818895_Trichoderma-reesei-MA2
GCA_001050175_Trichoderma-parareesei-CBS-125925
GCA_030708105_Trichoderma-sp-CBMAI-0711-CBMAI-0711
GCA_033030045_Trichoderma-parareesei-N4-3
GCA_020002365_Trichoderma-gracile-HK011-1
GCA_051819905_Trichoderma-gracile-CBS-130714
GCF_004382785_Trichoderma-ghanense-CCMA-1212
Trichoderma_effusum-DAOM230007_Scaffolds
GCA_022813565_Trichoderma-citrinoviride-FJI-L10-BK-P3
GCA_022813585_Trichoderma-citrinoviride-FJI-L10-BK-P1
GCF_003025115_Trichoderma-citrinoviride-TUCIM-6016
GCA_943193705_Trichoderma-citrinoviride-NA
GCA_047301815_Trichoderma-citrinoviride-GF-11
GCA_052425715_Trichoderma-citrinoviride-EB-13
Trichoderma_flagellatum-TUCIM3334_Scaffolds
Trichoderma_sinense-DAOM230004_Scaffolds
GCA_050948085_Trichoderma-novae-zelandiae-CBS-472-97
GCA_053477205_Trichoderma-deliquescens-Elizabeth-river
GCA_025919625_Trichoderma-arundinaceum-TP19-13
GCA_025919645_Trichoderma-arundinaceum-TP6-6
GCA_025919665_Trichoderma-arundinaceum-TP15-11
GCA_026401185_Trichoderma-arundinaceum-BJ183-35
GCA_003012085_Trichoderma-brevicompactum-IBT-40841
GCA_020631695_Trichoderma-cornu-damae-KA19-0412C
GCA_048182565_Trichoderma-asperellum-TRC-03
GCA_051820545_Trichoderma-sp-T77-T77
GCA_037954425_Trichoderma-asperellum-TCTP001
GCA_021066465_Trichoderma-asperelloides-T203
GCA_036173025_Trichoderma-asperellum-ICC-012
GCA_026401225_Trichoderma-asperelloides-BJ152-42
GCA_037041575_Trichoderma-asperelloides-PG5903
GCA_052084505_Trichoderma-asperelloides-X01119
GCA_054127785_Trichoderma-asperellum-T285
GCA_023628755_Trichoderma-asperellum-PT3102
GCA_037893215_Trichoderma-viride-Th4
GCA_019481625_Trichoderma-asperellum-TAIK5
GCA_019594925_Trichoderma-asperellum-TAIK4
GCA_052058355_Trichoderma-asperellum-TA1
GCA_013423425_Trichoderma-asperellum-IC-1
GCA_004154885_Trichoderma-asperellum-Ts93
GCA_014839745_Trichoderma-asperellum-SM-12F1
GCA_022817925_Trichoderma-asperellum-PK1J2
GCA_045838185_Trichoderma-yunnanense-TM10
GCA_000733085_Trichoderma-asperellum-B05
GCA_037177835_Trichoderma-asperellum-CKCT-S-1
GCA_034769445_Trichoderma-asperellum-TGT2021
GCA_019594945_Trichoderma-asperellum-TAIK1
GCA_020736895_Trichoderma-asperellum-IIPRTh-31
GCA_026401105_Trichoderma-asperellum-CGMCC-11653
GCF_003025105_Trichoderma-asperellum-CBS-433-97-CBS-433-97
GCF_020647865_Trichoderma-asperellum-FT101
GCA_049345875_Trichoderma-asperellum-CGMCC-41372
GCA_017945965_Trichoderma-asperellum-DQ-1
GCA_039880885_Trichoderma-asperellum-FT101
GCA_022817935_Trichoderma-sp-MLT1J1-MLT1J1
GCA_046127165_Trichoderma-hamatum-Z11
GCA_044646315_Trichoderma-harzianum-S5
GCA_000331835_Trichoderma-hamatum-GD12-GD12
GCA_054127445_Trichoderma-hamatum-Ta8426
GCA_049996655_Trichoderma-hamatum-G765-22
GCA_046629995_Trichoderma-hamatum-FBL-Z32
GCA_050947405_Trichoderma-evansii-SZMC-28006
GCA_022984945_Trichoderma-koningiopsis-RA6
GCA_022984975_Trichoderma-koningiopsis-RA5
GCA_022985005_Trichoderma-koningiopsis-RA3a
GCA_030247165_Trichoderma-koningiopsis-TK1
GCA_002246955_Trichoderma-koningiopsis-POS7
Trichoderma_taiwanense-IQ11_Scaffolds
GCA_048164925_Trichoderma-koningiopsis-Z35
GCA_049996695_Trichoderma-koningiopsis-G756-22
GCA_046255345_Trichoderma-koningiopsis-T-51
GCA_038502285_Trichoderma-sp-2475-2475
GCA_046580765_Trichoderma-virilente-Z53
GCA_049996625_Trichoderma-koningiopsis-G779-22
GCA_051820155_Trichoderma-sp-TUCIM-5745-TUCIM-5745
GCA_051820695_Trichoderma-sp-SZMC-28014-SZMC-28014
GCA_002894205_Trichoderma-gamsii-A5MH
GCA_049996675_Trichoderma-gamsii-G762-22
GCF_001481775_Trichoderma-gamsii-T6085
GCA_052570025_Trichoderma-viride-329365
GCA_052571625_Trichoderma-viride-315707
GCA_000963795_Trichoderma-atroviride-XS2015
GCA_051527675_Trichoderma-atroviride-CBS-122147
GCA_019297715_Trichoderma-atroviride-IMI-206040
GCF_020647795_Trichoderma-atroviride-P1
GCA_001599035_Trichoderma-atroviride-JCM-9410
GCA_051557965_Trichoderma-atroviride-B7
GCA_020466355_Trichoderma-atroviride-CG-6828
GCA_041053265_Trichoderma-atroviride-CGMCC-40488
GCA_028554805_Trichoderma-atroviride-Italy
GCA_002916895_Trichoderma-atroviride-LY357
GCA_040333345_Trichoderma-atroviride-F020
GCA_030708095_Trichoderma-sp-CBMAI-0020-CBMAI-0020
GCA_052570055_Trichoderma-viride-309685
GCA_052570065_Trichoderma-viride-238904
GCA_013365115_Trichoderma-erinaceum-CRRI-T2N1
GCA_026401215_Trichoderma-erinaceum-BJ212-21
Trichoderma_strigosellum-IQ191_Scaffolds
GCA_051820515_Trichoderma-austrokoningii-CBS-119092
GCA_052570145_Trichoderma-viride-90312
Trichoderma_minutisporum-DAOM175931_Scaffolds
GCA_030278935_Trichoderma-polysporum-HZ-31
GCA_051819845_Trichoderma-ceciliae-CBS-130010
GCA_015266385_Trichoderma-oligosporum-CGMCC-3-17527
GCA_023653515_Trichoderma-orchidacearum-COAD-3006

Figure S2. Expanded phylogenomic reconstruction of the genus Trichoderma. Detailed Maximum Likelihood phylogenomic tree of 208 Trichoderma genome assemblies inferred using IQ-TREE (v2.2.2) from a concatenated alignment of 920 conserved single-copy CDS partitions. All leaf nodes display full strain designations, public accession numbers, and taxid metadata. Branch support values at nodes represent 2,000 SH-aLRT replicates and 5,000 ultrafast bootstrap (UFBoot) replicates. Major phylogenomic clades (Clade 1, Clade 2A, Clade 2B, and Clade 2C) are color-coded. The tree is rooted using Trichoderma orchidacearum strain COAD-3006 (GCA_023653515) as an outgroup. Visualized and rendered using FigTree v1.4.4 (http://tree.bio.ed.ac.uk/software/figtree/).

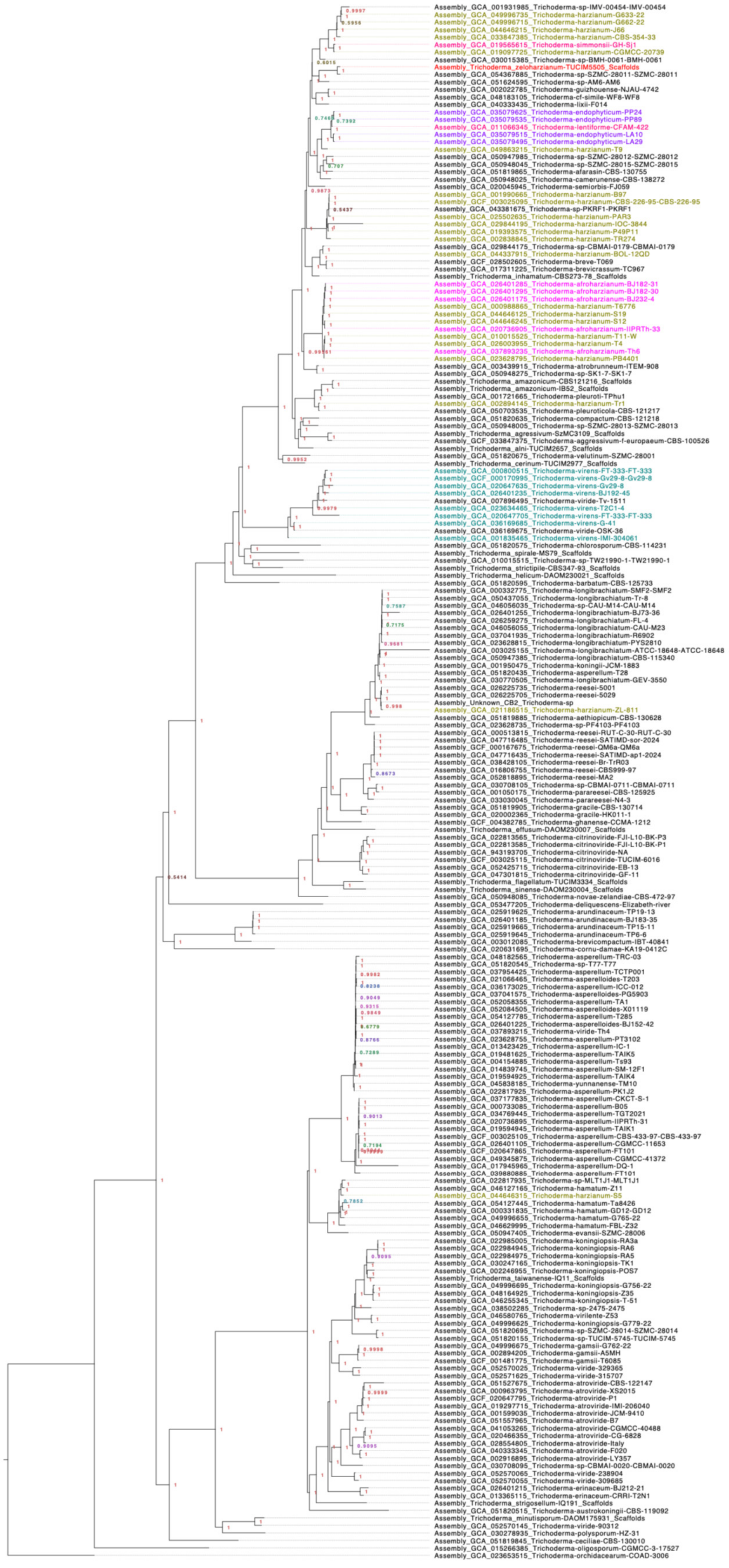

Assembly_GCA_001931985_Trichoderma-sp-IMV-00454-IMV-00454
Assembly_GCA_049996735_Trichoderma-harzianum-G633-22
Assembly_GCA_049996715_Trichoderma-harzianum-G662-22
Assembly_GCA_044646215_Trichoderma-harzianum-J66
Assembly_GCA_033847385_Trichoderma-harzianum-CBS-354-33
Assembly_GCA_019565615_Trichoderma-simmonsii-GH-Sj1
Assembly_GCA_019097725_Trichoderma-harzianum-CGMCC-20739
Assembly_GCA_030015385_Trichoderma-sp-BMH-0061-BMH-0061
Assembly_Trichoderma_zeloharzianum-TUCIM5505_Scaffolds
Assembly_GCA_054367885_Trichoderma-sp-SZMC-28011-SZMC-28011
Assembly_GCA_051624595_Trichoderma-sp-AM6-AM6
Assembly_GCA_002022785_Trichoderma-guizhouense-NJAU-4742
Assembly_GCA_048183105_Trichoderma-cf-simile-WF8-WF8
Assembly_GCA_040333435_Trichoderma-lixii-F014
Assembly_GCA_035079625_Trichoderma-endophyticum-PP24
Assembly_GCA_035079535_Trichoderma-endophyticum-PP89
Assembly_GCA_011066345_Trichoderma-lentiforme-CFAM-422
Assembly_GCA_035079515_Trichoderma-endophyticum-LA10
Assembly_GCA_035079495_Trichoderma-endophyticum-LA29
Assembly_GCA_049863215_Trichoderma-harzianum-T9
Assembly_GCA_050947985_Trichoderma-sp-SZMC-28012-SZMC-28012
Assembly_GCA_050948045_Trichoderma-sp-SZMC-28015-SZMC-28015
Assembly_GCA_051819865_Trichoderma-afarasin-CBS-130755
Assembly_GCA_050948025_Trichoderma-camerunense-CBS-138272
Assembly_GCA_020045945_Trichoderma-semiorbis-FJ059
Assembly_GCA_001990665_Trichoderma-harzianum-B97
Assembly_GCF_003025095_Trichoderma-harzianum-CBS-226-95-CBS-226-95
Assembly_GCA_043381675_Trichoderma-sp-PKRF1-PKRF1
Assembly_GCA_025502635_Trichoderma-harzianum-PAR3
Assembly_GCA_029844195_Trichoderma-harzianum-IOC-3844
Assembly_GCA_019393575_Trichoderma-harzianum-P49P11
Assembly_GCA_002838845_Trichoderma-harzianum-TR274
Assembly_GCA_029844175_Trichoderma-sp-CBMAI-0179-CBMAI-0179
Assembly_GCA_044337915_Trichoderma-harzianum-BOL-12QD
Assembly_GCF_028502605_Trichoderma-breve-T069
Assembly_GCA_017311225_Trichoderma-brevicrassum-TC967
Assembly_Trichoderma_inhamatum-CBS273-78_Scaffolds
Assembly_GCA_026401285_Trichoderma-afroharzianum-BJ182-31
Assembly_GCA_026401295_Trichoderma-afroharzianum-BJ182-30
Assembly_GCA_026401175_Trichoderma-afroharzianum-BJ232-4
Assembly_GCA_000988865_Trichoderma-harzianum-T6776
Assembly_GCA_044646125_Trichoderma-harzianum-S19
Assembly_GCA_044646245_Trichoderma-harzianum-S12
Assembly_GCA_020736905_Trichoderma-afroharzianum-IIPRTh-33
Assembly_GCA_010015525_Trichoderma-harzianum-T11-W
Assembly_GCA_026003955_Trichoderma-harzianum-T4
Assembly_GCA_037893235_Trichoderma-afroharzianum-Th6
Assembly_GCA_023628795_Trichoderma-harzianum-PB4401
Assembly_GCA_003439915_Trichoderma-atrobrunneum-ITEM-908
Assembly_GCA_050948275_Trichoderma-sp-SK1-7-SK1-7
Assembly_Trichoderma_amazonicum-CBS121216_Scaffolds
Assembly_Trichoderma_amazonicum-IB52_Scaffolds
Assembly_GCA_001721665_Trichoderma-pleuroti-TPhu1
Assembly_GCA_002894145_Trichoderma-harzianum-Tr1
Assembly_GCA_050703535_Trichoderma-pleuroticola-CBS-121217
Assembly_GCA_051820635_Trichoderma-compactum-CBS-121218
Assembly_GCA_050948005_Trichoderma-sp-SZMC-28013-SZMC-28013
Assembly_Trichoderma_agressivum-SzMC3109_Scaffolds
Assembly_GCF_033847375_Trichoderma-aggressivum-f-europaeum-CBS-100526
Assembly_Trichoderma_alni-TUCIM2657_Scaffolds
Assembly_GCA_051820675_Trichoderma-velutinum-SZMC-28001
Assembly_Trichoderma_cerinum-TUCIM2977_Scaffolds
Assembly_GCA_000800515_Trichoderma-virens-FT-333-FT-333
Assembly_GCF_000170995_Trichoderma-virens-Gv29-8-Gv29-8
Assembly_GCA_020647635_Trichoderma-virens-Gv29-8
Assembly_GCA_026401235_Trichoderma-virens-BJ192-45
Assembly_GCA_007896495_Trichoderma-viride-Tv-1511
Assembly_GCA_023634465_Trichoderma-virens-T2C1-4
Assembly_GCA_020647705_Trichoderma-virens-FT-333-FT-333
Assembly_GCA_036169685_Trichoderma-virens-G-41
Assembly_GCA_036169675_Trichoderma-viride-OSK-36
Assembly_GCA_001835465_Trichoderma-virens-IMI-304061
Assembly_GCA_051820575_Trichoderma-chlorosporum-CBS-114231
Assembly_Trichoderma_spirale-MS79_Scaffolds
Assembly_GCA_010015515_Trichoderma-sp-TW21990-1-TW21990-1
Assembly_Trichoderma_strictipile-CBS347-93_Scaffolds
Assembly_Trichoderma_helicum-DAOM230021_Scaffolds
Assembly_GCA_051820595_Trichoderma-barbatum-CBS-125733
Assembly_GCA_000332775_Trichoderma-longibrachiatum-SMF2-SMF2
Assembly_GCA_050437055_Trichoderma-longibrachiatum-Tr-8
Assembly_GCA_046056035_Trichoderma-sp-CAU-M14-CAU-M14
Assembly_GCA_026401255_Trichoderma-longibrachiatum-BJ73-36
Assembly_GCA_026259275_Trichoderma-longibrachiatum-FL-4
Assembly_GCA_046056055_Trichoderma-longibrachiatum-CAU-M23
Assembly_GCA_037041935_Trichoderma-longibrachiatum-R6902
Assembly_GCA_023628815_Trichoderma-longibrachiatum-PYS2810
Assembly_GCA_003025155_Trichoderma-longibrachiatum-ATCC-18648-ATCC-18648
Assembly_GCA_050947385_Trichoderma-longibrachiatum-CBS-115340
Assembly_GCA_001950475_Trichoderma-koningii-JCM-1883
Assembly_GCA_051820435_Trichoderma-asperellum-T28
Assembly_GCA_030770505_Trichoderma-longibrachiatum-GEV-3550
Assembly_GCA_026225735_Trichoderma-reesei-5001
Assembly_GCA_026225705_Trichoderma-reesei-5029
Assembly_Unknown_CB2_Trichoderma-sp
Assembly_GCA_021186515_Trichoderma-harzianum-ZL-811
Assembly_GCA_051819885_Trichoderma-aethiopicum-CBS-130628
Assembly_GCA_023628735_Trichoderma-sp-PF4103-PF4103
Assembly_GCA_000513815_Trichoderma-reesei-RUT-C-30-RUT-C-30
Assembly_GCA_047716485_Trichoderma-reesei-SATIMD-sor-2024
Assembly_GCF_000167675_Trichoderma-reesei-QM6a-QM6a
Assembly_GCA_047716435_Trichoderma-reesei-SATIMD-ap1-2024
Assembly_GCA_038428105_Trichoderma-reesei-Br-TrR03
Assembly_GCA_016806755_Trichoderma-reesei-CBS999-97
Assembly_GCA_052818895_Trichoderma-reesei-MA2
Assembly_GCA_030708105_Trichoderma-sp-CBMAI-0711-CBMAI-0711
Assembly_GCA_001050175_Trichoderma-parareesei-CBS-125925
Assembly_GCA_033030045_Trichoderma-parareesei-N4-3
Assembly_GCA_051819905_Trichoderma-gracile-CBS-130714
Assembly_GCA_020002365_Trichoderma-gracile-HK011-1
Assembly_GCF_004382785_Trichoderma-ghanense-CCMA-1212
Assembly_Trichoderma_effusum-DAOM230007_Scaffolds
Assembly_GCA_022813565_Trichoderma-citrinoviride-FJI-L10-BK-P3
Assembly_GCA_022813585_Trichoderma-citrinoviride-FJI-L10-BK-P1
Assembly_GCA_943193705_Trichoderma-citrinoviride-NA
Assembly_GCF_003025115_Trichoderma-citrinoviride-TUCIM-6016
Assembly_GCA_052425715_Trichoderma-citrinoviride-EB-13
Assembly_GCA_047301815_Trichoderma-citrinoviride-GF-11
Assembly_Trichoderma_flagellatum-TUCIM3334_Scaffolds
Assembly_Trichoderma_sinense-DAOM230004_Scaffolds
Assembly_GCA_050948085_Trichoderma-novae-zelandiae-CBS-472-97
Assembly_GCA_053477205_Trichoderma-deliquescens-Elizabeth-river
Assembly_GCA_025919625_Trichoderma-arundinaceum-TP19-13
Assembly_GCA_026401185_Trichoderma-arundinaceum-BJ183-35
Assembly_GCA_025919665_Trichoderma-arundinaceum-TP15-11
Assembly_GCA_025919645_Trichoderma-arundinaceum-TP6-6
Assembly_GCA_003012085_Trichoderma-brevicompactum-IBT-40841
Assembly_GCA_020631695_Trichoderma-cornu-damae-KA19-0412C
Assembly_GCA_048182565_Trichoderma-asperellum-TRC-03
Assembly_GCA_051820545_Trichoderma-sp-T77-T77
Assembly_GCA_037954425_Trichoderma-asperellum-TCTP001
Assembly_GCA_021066465_Trichoderma-asperelloides-T203
Assembly_GCA_036173025_Trichoderma-asperellum-ICC-012
Assembly_GCA_037041575_Trichoderma-asperelloides-PG5903
Assembly_GCA_052058355_Trichoderma-asperellum-TA1
Assembly_GCA_052084505_Trichoderma-asperelloides-X01119
Assembly_GCA_054127785_Trichoderma-asperellum-T285
Assembly_GCA_026401225_Trichoderma-asperelloides-BJ152-42
Assembly_GCA_037893215_Trichoderma-viride-Th4
Assembly_GCA_023628755_Trichoderma-asperellum-PT3102
Assembly_GCA_013423425_Trichoderma-asperellum-IC-1
Assembly_GCA_019481625_Trichoderma-asperellum-TAIK5
Assembly_GCA_004154885_Trichoderma-asperellum-Ts93
Assembly_GCA_014839745_Trichoderma-asperellum-SM-12F1
Assembly_GCA_019594925_Trichoderma-asperellum-TAIK4
Assembly_GCA_045838185_Trichoderma-yunnanense-TM10
Assembly_GCA_022817925_Trichoderma-asperellum-PK1J2
Assembly_GCA_037177835_Trichoderma-asperellum-CKCT-S-1
Assembly_GCA_000733085_Trichoderma-asperellum-B05
Assembly_GCA_034769445_Trichoderma-asperellum-TGT2021
Assembly_GCA_020736895_Trichoderma-asperellum-IIPRTh-31
Assembly_GCA_019594945_Trichoderma-asperellum-TAIK1
Assembly_GCF_003025105_Trichoderma-asperellum-CBS-433-97-CBS-433-97
Assembly_GCA_026401105_Trichoderma-asperellum-CGMCC-11653
Assembly_GCF_020647865_Trichoderma-asperellum-FT101
Assembly_GCA_049345875_Trichoderma-asperellum-CGMCC-41372
Assembly_GCA_017945965_Trichoderma-asperellum-DQ-1
Assembly_GCA_039880885_Trichoderma-asperellum-FT101
Assembly_GCA_022817935_Trichoderma-sp-MLT1J1-MLT1J1
Assembly_GCA_046127165_Trichoderma-hamatum-Z11
Assembly_GCA_044646315_Trichoderma-harzianum-S5
Assembly_GCA_054127445_Trichoderma-hamatum-Ta8426
Assembly_GCA_000331835_Trichoderma-hamatum-GD12-GD12
Assembly_GCA_049996655_Trichoderma-hamatum-G765-22
Assembly_GCA_046629995_Trichoderma-hamatum-FBL-Z32
Assembly_GCA_050947405_Trichoderma-evansii-SZMC-28006
Assembly_GCA_022985005_Trichoderma-koningiopsis-RA3a
Assembly_GCA_022984945_Trichoderma-koningiopsis-RA6
Assembly_GCA_022984975_Trichoderma-koningiopsis-RA5
Assembly_GCA_030247165_Trichoderma-koningiopsis-TK1
Assembly_GCA_002246955_Trichoderma-koningiopsis-POS7
Assembly_Trichoderma_taiwanense-IQ11_Scaffolds
Assembly_GCA_049996695_Trichoderma-koningiopsis-G756-22
Assembly_GCA_048164925_Trichoderma-koningiopsis-Z35
Assembly_GCA_046255345_Trichoderma-koningiopsis-T-51
Assembly_GCA_038502285_Trichoderma-sp-2475-2475
Assembly_GCA_046580765_Trichoderma-virilente-Z53
Assembly_GCA_049996625_Trichoderma-koningiopsis-G779-22
Assembly_GCA_051820695_Trichoderma-sp-SZMC-28014-SZMC-28014
Assembly_GCA_051820155_Trichoderma-sp-TUCIM-5745-TUCIM-5745
Assembly_GCA_049996675_Trichoderma-gamsii-G762-22
Assembly_GCA_002894205_Trichoderma-gamsii-A5MH
Assembly_GCF_001481775_Trichoderma-gamsii-T6085
Assembly_GCA_052570025_Trichoderma-viride-329365
Assembly_GCA_052571625_Trichoderma-viride-315707
Assembly_GCA_051527675_Trichoderma-atroviride-CBS-122147
Assembly_GCA_000963795_Trichoderma-atroviride-XS2015
Assembly_GCF_020647795_Trichoderma-atroviride-P1
Assembly_GCA_019297715_Trichoderma-atroviride-IMI-206040
Assembly_GCA_001599035_Trichoderma-atroviride-JCM-9410
Assembly_GCA_051557965_Trichoderma-atroviride-B7
Assembly_GCA_041053265_Trichoderma-atroviride-CGMCC-40488
Assembly_GCA_020466355_Trichoderma-atroviride-CG-6828
Assembly_GCA_028554805_Trichoderma-atroviride-Italy
Assembly_GCA_040333345_Trichoderma-atroviride-F020
Assembly_GCA_002916895_Trichoderma-atroviride-LY357
Assembly_GCA_030708095_Trichoderma-sp-CBMAI-0020-CBMAI-0020
Assembly_GCA_052570065_Trichoderma-viride-238904
Assembly_GCA_052570055_Trichoderma-viride-309685
Assembly_GCA_026401215_Trichoderma-erinaceum-BJ212-21
Assembly_GCA_013365115_Trichoderma-erinaceum-CRRI-T2N1
Assembly_Trichoderma_strigosellum-IQ191_Scaffolds
Assembly_GCA_051820515_Trichoderma-austrokoningii-CBS-119092
Assembly_Trichoderma_minutisporum-DAOM175931_Scaffolds
Assembly_GCA_052570145_Trichoderma-viride-90312
Assembly_GCA_030278935_Trichoderma-polysporum-HZ-31
Assembly_GCA_051819845_Trichoderma-ceciliae-CBS-130010
Assembly_GCA_015266385_Trichoderma-oligosporum-CGMCC-3-17527
Assembly_GCA_023653515_Trichoderma-orchidacearum-COAD-3006

Figure S3. Complete, uncollapsed ASTRAL summary-coalescent species tree of 208 Trichoderma genomes inferred from 920 single-copy orthologs. High-resolution species tree estimated under the Multi-Species Coalescent (MSC) framework using ASTER/ASTRAL (v5.7.8) based on 920 individual maximum-likelihood gene trees generated from single-copy CDS loci. Leaves display strain identifiers and NCBI accession numbers across all 208 analyzed genomes without node collapsing. Branch support values indicate Local Posterior Probabilities (LPP), with values ⩾ 0.95 reflecting strong topological confidence under coalescent theory. Tree topology was rendered and styled using FigTree v1.4.4 (http://tree.bio.ed.ac.uk/software/figtree/).

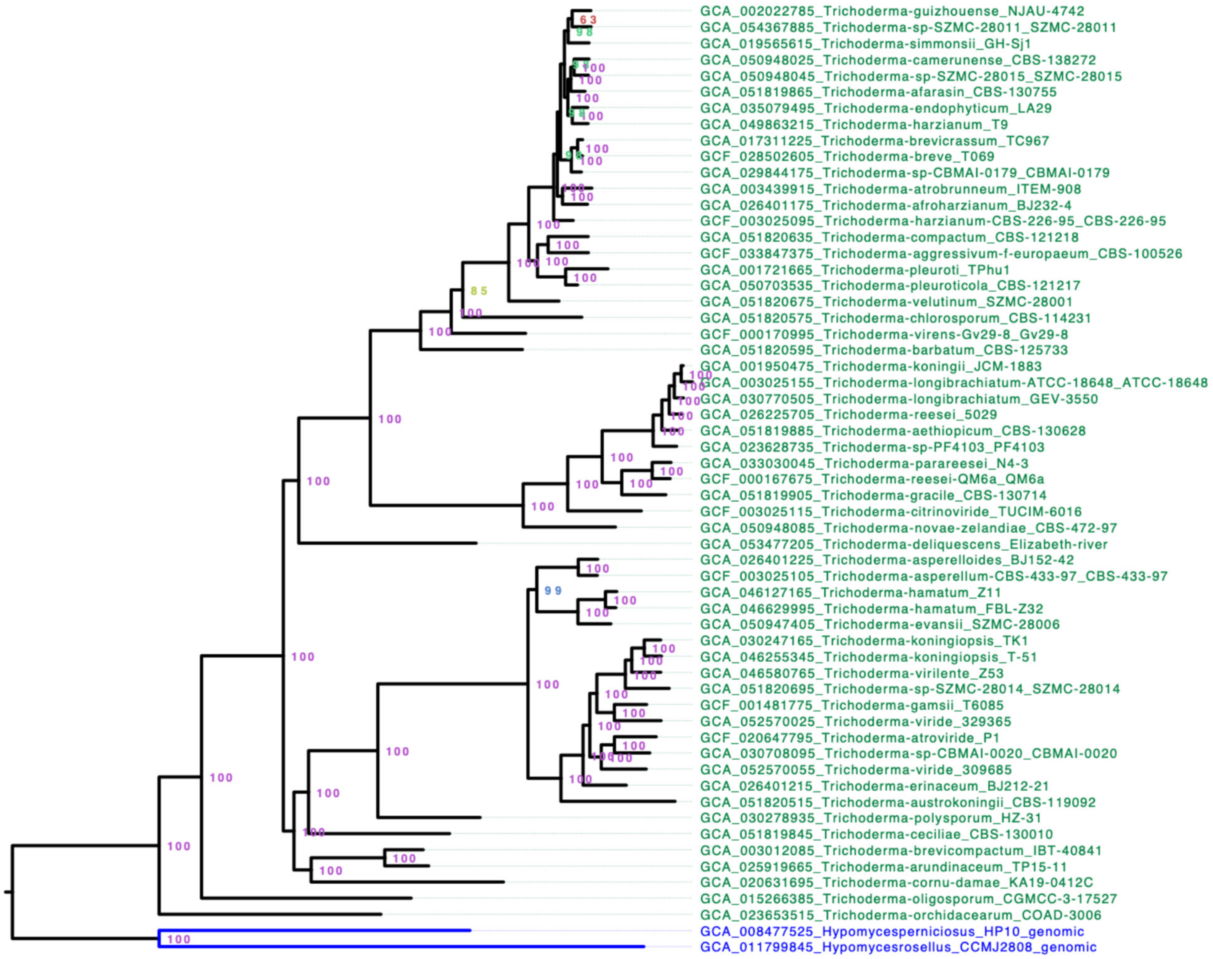

Figure S4. Outgroup-rooted Maximum Likelihood phylogenomic tree of *Trichoderma* and *Hypomyces* inferred from 3,577 single-copy orthologous proteins. Maximum Likelihood species tree inferred across 59 proteomes (57 representative *Trichoderma* genomes and two outgroup taxa, *Hypomyces perniciosus* HP10 (GCA_008477525) and *Hypomyces rosellus* CCMJ2808 (GCA_011799845)). Single-copy orthologous protein loci were identified using SonicParanoid (v1.3.8) (conserved in ≥ 80% of proteomes). The concatenated alignment of 3,577 protein loci was analyzed in IQ-TREE (v2.2.2) under the LG4M empirical mixture model with 5,000 ultrafast bootstrap replicates. Node values represent UFBoot support percentages. Tree visualized in FigTree v1.4.4 (http://tree.bio.ed.ac.uk/software/figtree/).

Table Legends

Table S1. Genome-specific information on the origin and extent of contaminating contigs. Detailed breakdown of identified contaminant sequence fragments across public *Trichoderma* assemblies, including total contigs removed, total sequence length purged (bp), and taxonomic assignment of contamination.

Table S2. Genome accessions and general genomic statistics. Relevant metadata and assembly metrics for the 208 analyzed *Trichoderma* genomes. Fields include NCBI GenBank assembly accession numbers (https://www.ncbi.nlm.nih.gov/genbank/), reported species annotations, geographic country of origin, assigned phylogenomic clade (Clade 1, 2A, 2B, or 2C), total assembly size (Mb), GC content (%), contig N50 (kb), BUSCO completeness (%), BUSCO multicopy (%), masked repeat content (%), and total annotated gene count.

Table S3. Pairwise whole-genome alignment metrics, sequence identity, and single-nucleotide variants (SNP) counts across analyzed *Trichoderma* clade 2B genomes. Summary of pairwise genome-wide synteny and divergence statistics among Clade 2B assemblies. Metrics include aligned genome fraction/bases (%), average nucleotide identity (%), total single-nucleotide polymorphism (SNP) counts on aligned fraction.

Table S4. Functional classification and domain profiling (KEGG, COG, Pfam) of conserved *Trichoderma* core orthogroups. Detailed functional annotation of core orthologous gene clusters identified across the genus *Trichoderma*. Columns list orthogroup IDs, Kyoto Encyclopedia of Genes and Genomes (KEGG) Orthology (KO) terms (https://www.kegg.jp/, accessed May 2026), Clusters of Orthologous Groups (COG) functional categories, and Pfam protein domain accessions.

Figure S1: Comparison of genome assembly metrics before and after filtering

Figure S2: Expanded phylogenomic reconstruction of the genus Trichoderma

Figure S3: Complete, uncollapsed ASTRAL summary-coalescent species tree of 208 Trichoderma genomes inferred from 920 single-copy orthologs

Figure S4. Outgroup-rooted Maximum Likelihood phylogenomic tree of Trichoderma and *Hypomyces* inferred from 3,577 single-copy orthologous proteins

Table S1: Genome-specific information on the origin and extent of contaminating contigs

Table S2: Genome accessions and general genomic statistics

Table S3: Pairwise whole-genome alignment metrics, sequence identity, and single-nucleotide polymorphism (SNP) counts across analyzed Trichoderma clade 2B genomes

Table S4: Functional classification and domain profiling (KEGG, COG, Pfam) of conserved Trichoderma core orthogroups
