# Supplementary material for: Phylogenomic and Comparative Genomic Analyses Reveal Deep Evolutionary Structure and Cryptic Diversity in the Genus Trichoderma
Source: Int J Mol Sci. 2026 Sep 15;27(18):8204. doi: 10.3390/ijms27188204 (PMC13607837; doi:10.3390/ijms27188204)
Supplement: Supplementary file 1 [file ijms-27-08204-s001.zip › Supp_Fig_S1.pdf]

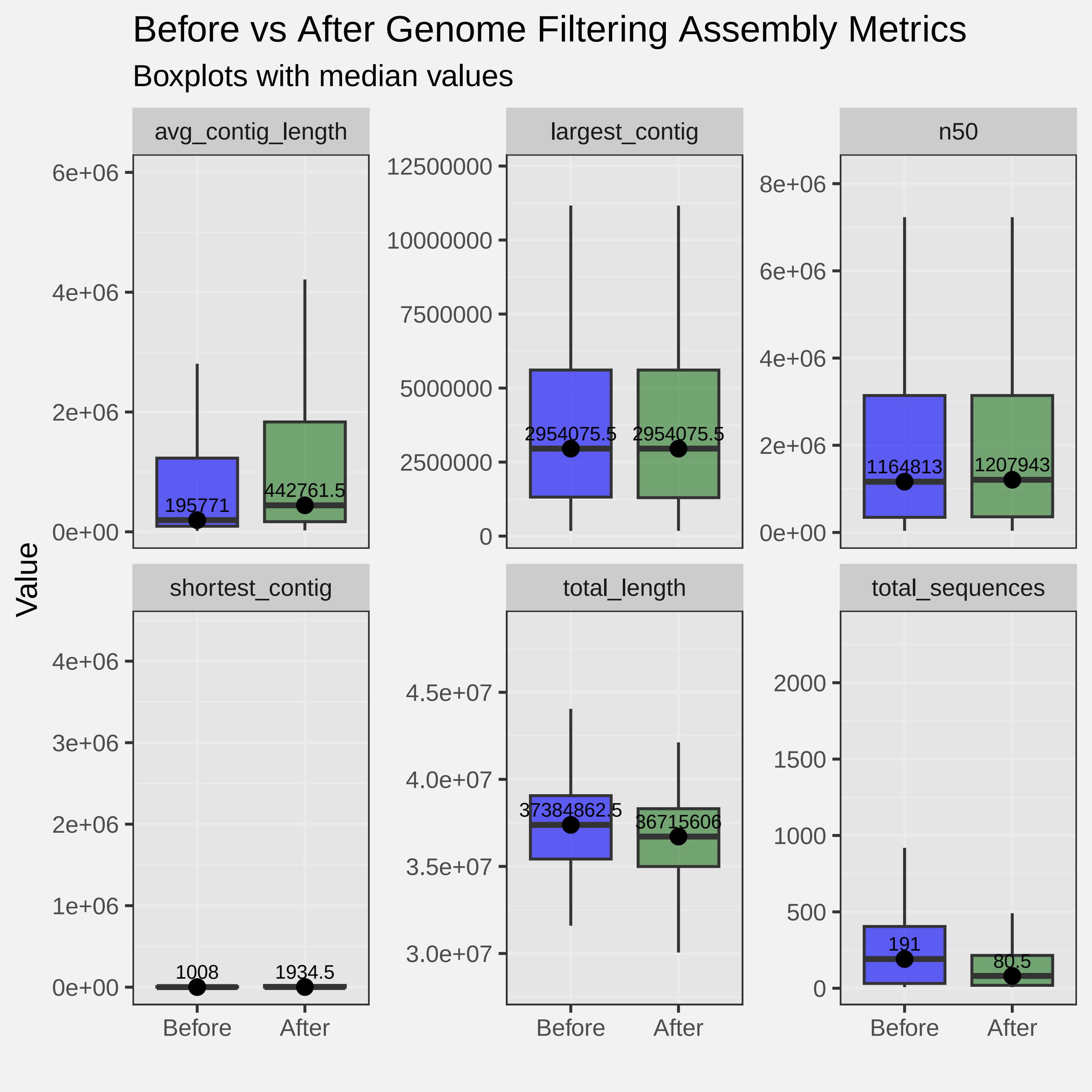

Before vs After Genome Filtering Assembly Metrics
Boxplots with median values
Value
avg_contig_length
6e+06
4e+06
2e+06
0e+00
195771
442761.5
largest_contig
12500000
10000000
7500000
5000000
2500000
0
2954075.5
2954075.5
n50
8e+06
6e+06
4e+06
2e+06
0e+00
1164813
1207943
shortest_contig
4e+06
3e+06
2e+06
1e+06
0e+00
1008
1934.5
total_length
4.5e+07
4.0e+07
3.5e+07
3.0e+07
37384862.5
36715606
total_sequences
2000
1500
1000
500
0
191
80.5
Before
After
Before
After
Before
After
