# Supplementary material for: Phylogenomic and Comparative Genomic Analyses Reveal Deep Evolutionary Structure and Cryptic Diversity in the Genus Trichoderma
Source: Int J Mol Sci. 2026 Sep 15;27(18):8204. doi: 10.3390/ijms27188204 (PMC13607837; doi:10.3390/ijms27188204)
Supplement: Supplementary file 1 [file ijms-27-08204-s001.zip › Supp_Fig_S2.pdf]

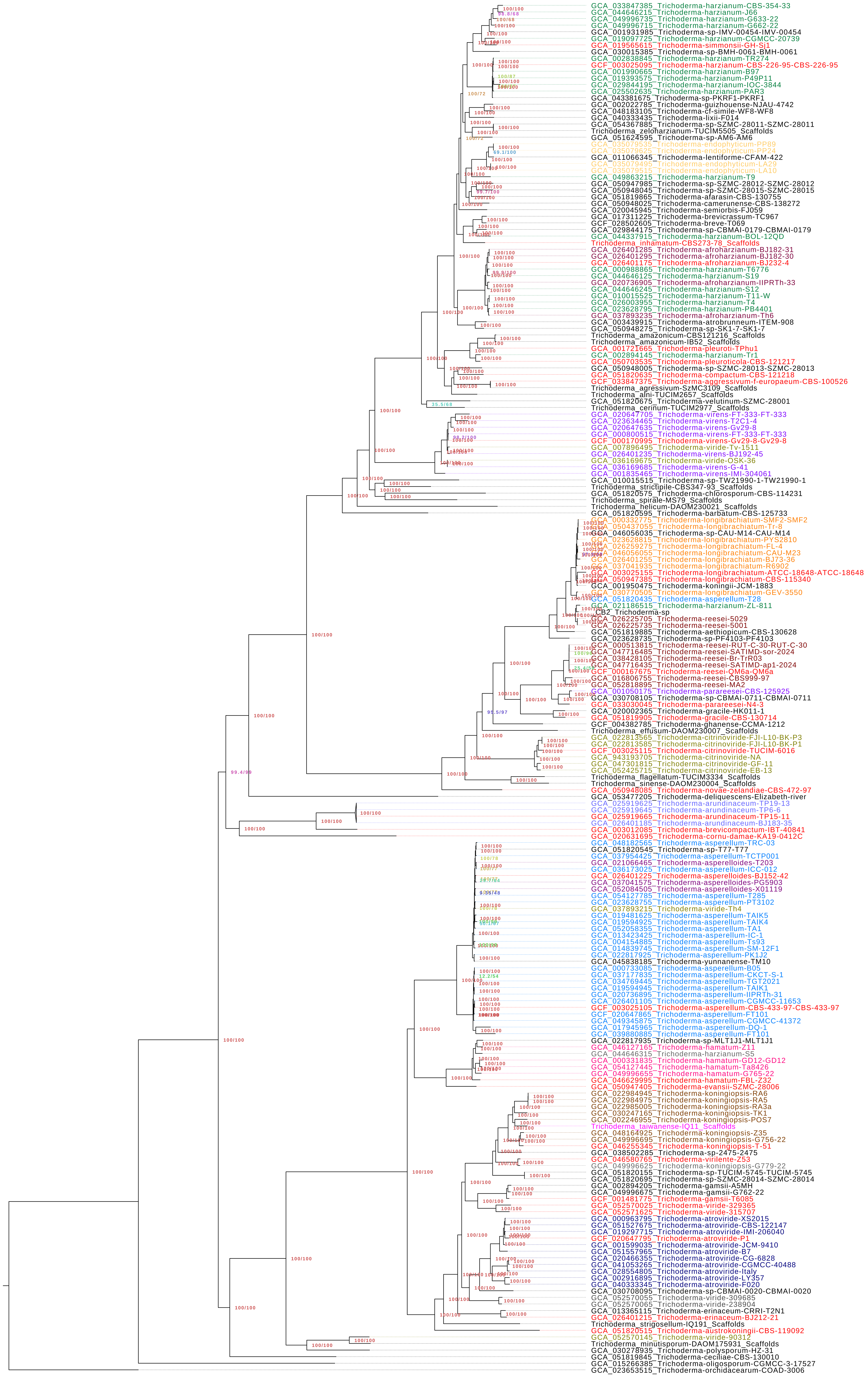
GCA_033847385_Trichoderma-harzianum-CBS-354-33
GCA_044646215_Trichoderma-harzianum-J66
GCA_049996735_Trichoderma-harzianum-G633-22
GCA_049996715_Trichoderma-harzianum-G662-22
GCA_001931985_Trichoderma-sp-IMV-00454-IMV-00454
GCA_019097725_Trichoderma-harzianum-CGMCC-20739
GCA_019565615_Trichoderma-simmonsii-GH-Sj1
GCA_030015385_Trichoderma-sp-BMH-0061-BMH-0061
GCA_002838845_Trichoderma-harzianum-TR274
GCF_003025095_Trichoderma-harzianum-CBS-226-95-CBS-226-95
GCA_001990665_Trichoderma-harzianum-B97
GCA_019393575_Trichoderma-harzianum-P49P11
GCA_029844195_Trichoderma-harzianum-IIOC-3844
GCA_025502635_Trichoderma-harzianum-PAR3
GCA_043381675_Trichoderma-sp-PKRF1-PKRF1
GCA_002022785_Trichoderma-guizhouense-NJAU-4742
GCA_048183105_Trichoderma-cf-simile-WF8-WF8
GCA_040333435_Trichoderma-lixii-F014
GCA_054367885_Trichoderma-sp-SZMC-28011-SZMC-28011
Trichoderma_zeloharzianum-TUCIM5505_Scaffolds
GCA_051624595_Trichoderma-sp-AM6-AM6
GCA_035079535_Trichoderma-endophyticum-PP89
GCA_035079525_Trichoderma-endophyticum-PP24
GCA_011066345_Trichoderma-lentiforme-CFAM-422
GCA_035079495_Trichoderma-endophyticum-LA29
GCA_035079515_Trichoderma-endophyticum-LA10
GCA_049863215_Trichoderma-harzianum-T9
GCA_050947985_Trichoderma-sp-SZMC-28012-SZMC-28012
GCA_050948045_Trichoderma-sp-SZMC-28015-SZMC-28015
GCA_051819865_Trichoderma-afarasin-CBS-130755
GCA_050948025_Trichoderma-camerunense-CBS-138272
GCA_020045945_Trichoderma-semiorbis-FJ059
GCA_017311225_Trichoderma-brevicrassum-TC967
GCF_028502605_Trichoderma-breve-T069
GCA_029844175_Trichoderma-sp-CBMAI-0179-CBMAI-0179
GCA_044337915_Trichoderma-harzianum-BOL-12QD
Trichoderma_inhamatum-CBS273-78_Scaffolds
GCA_026401285_Trichoderma-afroharzianum-BJ182-31
GCA_026401295_Trichoderma-afroharzianum-BJ182-30
GCA_026401175_Trichoderma-afroharzianum-BJ232-4
GCA_000988865_Trichoderma-harzianum-T6776
GCA_044646125_Trichoderma-harzianum-S19
GCA_020736905_Trichoderma-afroharzianum-IIPRTh-33
GCA_044646245_Trichoderma-harzianum-S12
GCA_010015525_Trichoderma-harzianum-T11-W
GCA_026003955_Trichoderma-harzianum-T4
GCA_023628795_Trichoderma-harzianum-PB4401
GCA_037893235_Trichoderma-afroharzianum-Th6
GCA_003439915_Trichoderma-atrobrunneum-ITEM-908
GCA_050948275_Trichoderma-sp-SK1-7-SK1-7
Trichoderma_amazonicum-CBS121216_Scaffolds
Trichoderma_amazonicum-IB52_Scaffolds
GCA_001721665_Trichoderma-pleuroti-TPhu1
GCA_002894145_Trichoderma-harzianum-Tr1
GCA_050703535_Trichoderma-pleuroticola-CBS-121217
GCA_050948005_Trichoderma-sp-SZMC-28013-SZMC-28013
GCA_051820635_Trichoderma-compactum-CBS-121218
GCF_033847375_Trichoderma-aggressivum-f-europaeum-CBS-100526
Trichoderma_agressivum-SzMC3109_Scaffolds
Trichoderma_alni-TUCIM2657_Scaffolds
GCA_051820675_Trichoderma-velutinum-SZMC-28001
Trichoderma_cerinum-TUCIM2977_Scaffolds
GCA_020647705_Trichoderma-virens-FT-333-FT-333
GCA_023634465_Trichoderma-virens-T2C1-4
GCA_020647635_Trichoderma-virens-Gv29-8
GCA_000800515_Trichoderma-virens-FT-333-FT-333
GCF_000170995_Trichoderma-virens-Gv29-8-Gv29-8
GCA_007896495_Trichoderma-viride-Tv-1511
GCA_026401235_Trichoderma-virens-BJ192-45
GCA_036169675_Trichoderma-viride-OSK-36
GCA_036169685_Trichoderma-virens-G-41
GCA_001835465_Trichoderma-virens-IMI-304061
GCA_010015515_Trichoderma-sp-TW21990-1-TW21990-1
Trichoderma_strictipile-CBS347-93_Scaffolds
GCA_051820575_Trichoderma-chlorosporum-CBS-114231
Trichoderma_spirale-MS79_Scaffolds
Trichoderma_helicum-DAOM230021_Scaffolds
GCA_051820595_Trichoderma-barbatum-CBS-125733
GCA_000332775_Trichoderma-longibrachiatum-SMF2-SMF2
GCA_050437055_Trichoderma-longibrachiatum-Tr-8
GCA_046056035_Trichoderma-sp-CAU-M14-CAU-M14
GCA_023628815_Trichoderma-longibrachiatum-PYS2810
GCA_026259275_Trichoderma-longibrachiatum-FL-4
GCA_046056055_Trichoderma-longibrachiatum-CAU-M23
GCA_026401255_Trichoderma-longibrachiatum-BJ73-36
GCA_037041935_Trichoderma-longibrachiatum-R6902
GCA_003025155_Trichoderma-longibrachiatum-ATCC-18648-ATCC-18648
GCA_050947385_Trichoderma-longibrachiatum-CBS-115340
GCA_001950475_Trichoderma-koningii-JCM-1883
GCA_030770505_Trichoderma-longibrachiatum-GEV-3550
GCA_051820435_Trichoderma-asperellum-T28
GCA_021186515_Trichoderma-harzianum-ZL-811
CB2_Trichoderma-sp
GCA_026225705_Trichoderma-reesei-5029
GCA_026225735_Trichoderma-reesei-5001
GCA_051819885_Trichoderma-aethiopicum-CBS-130628
GCA_023628735_Trichoderma-sp-PF4103-PF4103
GCA_000513815_Trichoderma-reesei-RUT-C-30-RUT-C-30
GCA_047716485_Trichoderma-reesei-SATIMD-sor-2024
GCA_038428105_Trichoderma-reesei-Br-TrR03
GCA_047716435_Trichoderma-reesei-SATIMD-ap1-2024
GCF_000167675_Trichoderma-reesei-QM6a-QM6a
GCA_016806755_Trichoderma-reesei-CBS999-97
GCA_052818895_Trichoderma-reesei-MA2
GCA_001050175_Trichoderma-parareesei-CBS-125925
GCA_030708105_Trichoderma-sp-CBMAI-0711-CBMAI-0711
GCA_033030045_Trichoderma-parareesei-N4-3
GCA_020002365_Trichoderma-gracile-HK011-1
GCA_051819905_Trichoderma-gracile-CBS-130714
GCF_004382785_Trichoderma-ghanense-CCMA-1212
Trichoderma_effusum-DAOM230007_Scaffolds
GCA_022813565_Trichoderma-citrinoviride-FJI-L10-BK-P3
GCA_022813585_Trichoderma-citrinoviride-FJI-L10-BK-P1
GCF_003025115_Trichoderma-citrinoviride-TUCIM-6016
GCA_943193705_Trichoderma-citrinoviride-NA
GCA_047301815_Trichoderma-citrinoviride-GF-11
GCA_052425715_Trichoderma-citrinoviride-EB-13
Trichoderma_flagellatum-TUCIM3334_Scaffolds
Trichoderma_sinense-DAOM230004_Scaffolds
GCA_050948085_Trichoderma-novae-zelandiae-CBS-472-97
GCA_053477205_Trichoderma-deliquescens-Elizabeth-river
GCA_025919625_Trichoderma-arundinaceum-TP19-13
GCA_025919645_Trichoderma-arundinaceum-TP6-6
GCA_025919665_Trichoderma-arundinaceum-TP15-11
GCA_026401185_Trichoderma-arundinaceum-BJ133-35
GCA_003012085_Trichoderma-brevicompactum-IBT-40841
GCA_020631695_Trichoderma-cornu-damae-KA19-0412C
GCA_048182565_Trichoderma-asperellum-TRC-03
GCA_051820545_Trichoderma-sp-T77-T77
GCA_037954425_Trichoderma-asperellum-TCTP001
GCA_021066465_Trichoderma-asperelloides-T203
GCA_036173025_Trichoderma-asperellum-ICC-012
GCA_026401225_Trichoderma-asperelloides-BJ152-42
GCA_037041575_Trichoderma-asperelloides-PG5903
GCA_052084505_Trichoderma-asperelloides-X01119
GCA_054127785_Trichoderma-asperellum-T285
GCA_023628755_Trichoderma-asperellum-PT3102
GCA_037893215_Trichoderma-viride-Th4
GCA_019481625_Trichoderma-asperellum-TAIK5
GCA_019594925_Trichoderma-asperellum-TAIK4
GCA_052058355_Trichoderma-asperellum-TA1
GCA_013423425_Trichoderma-asperellum-IC-1
GCA_004154885_Trichoderma-asperellum-Ts93
GCA_014839745_Trichoderma-asperellum-SM-12F1
GCA_022817925_Trichoderma-asperellum-PK1J2
GCA_045838185_Trichoderma-yunnanense-TM10
GCA_000733085_Trichoderma-asperellum-B05
GCA_037177835_Trichoderma-asperellum-CKCT-S-1
GCA_034769445_Trichoderma-asperellum-TGT2021
GCA_019594945_Trichoderma-asperellum-TAIK1
GCA_020736895_Trichoderma-asperellum-IIPRTh-31
GCA_026401105_Trichoderma-asperellum-CGMCC-11653
GCF_003025105_Trichoderma-asperellum-CBS-433-97-CBS-433-97
GCF_020647865_Trichoderma-asperellum-FT101
GCA_049345875_Trichoderma-asperellum-CGMCC-41372
GCA_017945965_Trichoderma-asperellum-DQ-1
GCA_039880885_Trichoderma-asperellum-FT101
GCA_022817935_Trichoderma-sp-MLT1J1-MLT1J1
GCA_046127165_Trichoderma-hamatum-Z11
GCA_044646315_Trichoderma-harzianum-S5
GCA_000331835_Trichoderma-hamatum-GD12-GD12
GCA_054127445_Trichoderma-hamatum-Ta8426
GCA_049996655_Trichoderma-hamatum-G765-22
GCA_046629995_Trichoderma-hamatum-FBL-Z32
GCA_050947405_Trichoderma-evansii-SZMC-28006
GCA_022984945_Trichoderma-koningiopsis-RA6
GCA_022984975_Trichoderma-koningiopsis-RA5
GCA_022985005_Trichoderma-koningiopsis-RA3a
GCA_030247165_Trichoderma-koningiopsis-TK1
GCA_002246955_Trichoderma-koningiopsis-POS7
Trichoderma_taiwanense-IQ11_Scaffolds
GCA_048164925_Trichoderma-koningiopsis-Z35
GCA_049996695_Trichoderma-koningiopsis-G756-22
GCA_046255345_Trichoderma-koningiopsis-T-51
GCA_038502285_Trichoderma-sp-2475-2475
GCA_046580765_Trichoderma-virilente-Z53
GCA_049996625_Trichoderma-koningiopsis-G779-22
GCA_051820155_Trichoderma-sp-TUCIM-5745-TUCIM-5745
GCA_051820695_Trichoderma-sp-SZMC-28014-SZMC-28014
GCA_002894205_Trichoderma-gamsii-A5MH
GCA_049996675_Trichoderma-gamsii-G762-22
GCF_001481775_Trichoderma-gamsii-T6085
GCA_052570025_Trichoderma-viride-329365
GCA_052571625_Trichoderma-viride-315707
GCA_000963795_Trichoderma-atroviride-XS2015
GCA_051527675_Trichoderma-atroviride-CBS-122147
GCA_019297715_Trichoderma-atroviride-IMI-206040
GCF_020647795_Trichoderma-atroviride-P1
GCA_001599035_Trichoderma-atroviride-JCM-9410
GCA_051557965_Trichoderma-atroviride-B7
GCA_020466355_Trichoderma-atroviride-CG-6828
GCA_041053265_Trichoderma-atroviride-CGMCC-40488
GCA_028554805_Trichoderma-atroviride-Italy
GCA_002916895_Trichoderma-atroviride-LY357
GCA_040333345_Trichoderma-atroviride-F020
GCA_030708095_Trichoderma-sp-CBMAI-0020-CBMAI-0020
GCA_052570055_Trichoderma-viride-309685
GCA_052570065_Trichoderma-viride-238904
GCA_013365115_Trichoderma-erinaceum-CRRI-T2N1
GCA_026401215_Trichoderma-erinaceum-BJ212-21
Trichoderma_strigosellum-IQ191_Scaffolds
GCA_051820515_Trichoderma-austrokoningii-CBS-119092
GCA_052570145_Trichoderma-viride-90312
Trichoderma_minutisporum-DAOM175931_Scaffolds
GCA_030278935_Trichoderma-polysporum-HZ-31
GCA_051819845_Trichoderma-ceciliae-CBS-130010
GCA_015266385_Trichoderma-oligosporum-CGMCC-3-17527
GCA_023653515_Trichoderma-orchidacearum-COAD-3006
