# Supplementary material for: Phylogenomic and Comparative Genomic Analyses Reveal Deep Evolutionary Structure and Cryptic Diversity in the Genus Trichoderma
Source: Int J Mol Sci. 2026 Sep 15;27(18):8204. doi: 10.3390/ijms27188204 (PMC13607837; doi:10.3390/ijms27188204)
Supplement: Supplementary file 1 [file ijms-27-08204-s001.zip › Supp_Fig_S3.pdf]

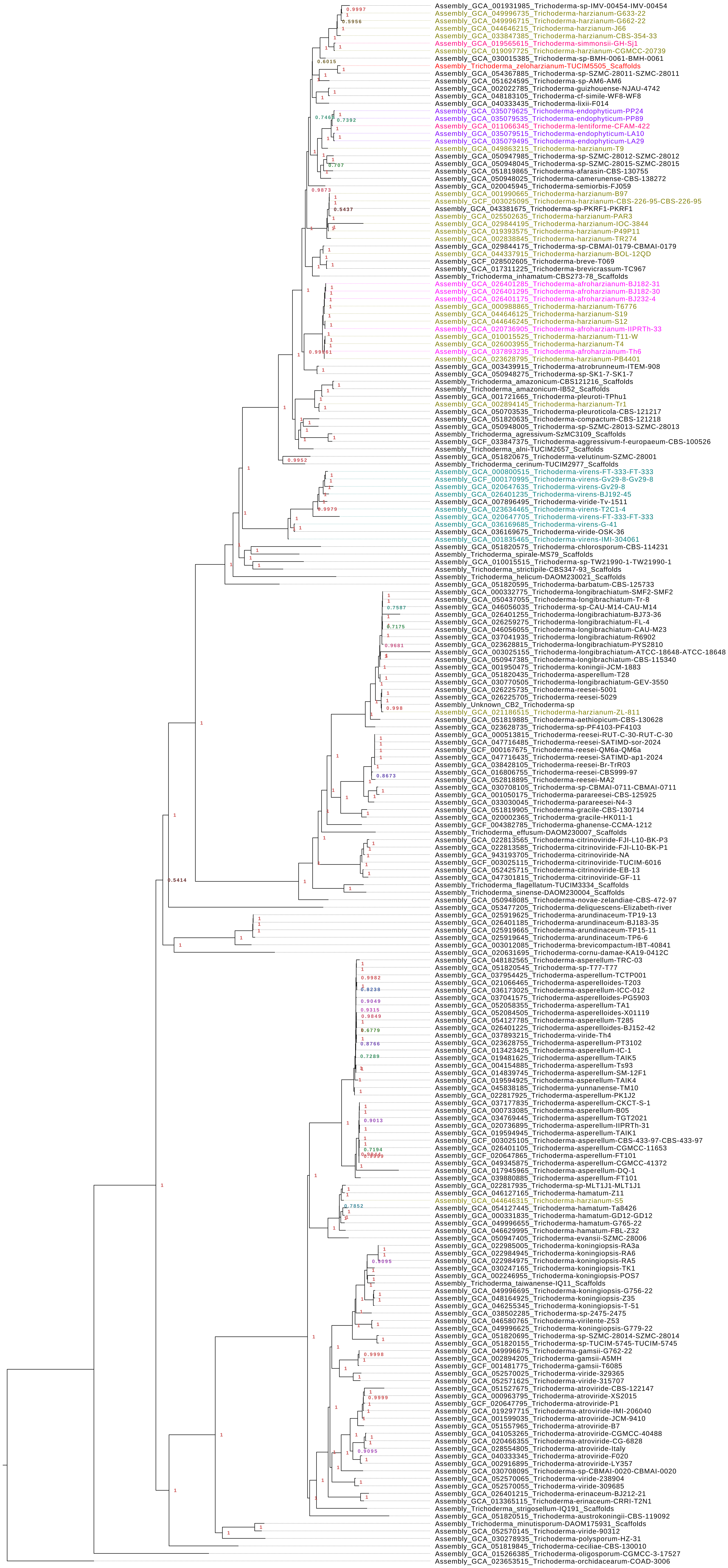
Assembly_GCA_001931985_Trichoderma-sp-IMV-00454-IMV-00454
Assembly_GCA_049996735_Trichoderma-harzianum-G633-22
Assembly_GCA_049996715_Trichoderma-harzianum-G662-22
Assembly_GCA_044646215_Trichoderma-harzianum-J66
Assembly_GCA_033847385_Trichoderma-harzianum-CBS-354-33
Assembly_GCA_019565615_Trichoderma-simmonsii-GH-Sj1
Assembly_GCA_019097725_Trichoderma-harzianum-CGMCC-20739
Assembly_GCA_030015385_Trichoderma-sp-BMH-0061-BMH-0061
Assembly_Trichoderma_zeloharzianum-TUCIM5505_Scaffolds
Assembly_GCA_054367885_Trichoderma-sp-SZMC-28011-SZMC-28011
Assembly_GCA_051624595_Trichoderma-sp-AM6-AM6
Assembly_GCA_002022785_Trichoderma-guizhouense-NJAU-4742
Assembly_GCA_048183105_Trichoderma-cf-simile-WF8-WF8
Assembly_GCA_040333435_Trichoderma-lixii-F014
Assembly_GCA_035079625_Trichoderma-endophyticum-PP24
Assembly_GCA_035079535_Trichoderma-endophyticum-PP89
Assembly_GCA_011066345_Trichoderma-lentiforme-CFAM-422
Assembly_GCA_035079515_Trichoderma-endophyticum-LA10
Assembly_GCA_035079495_Trichoderma-endophyticum-LA29
Assembly_GCA_049863215_Trichoderma-harzianum-T9
Assembly_GCA_050947985_Trichoderma-sp-SZMC-28012-SZMC-28012
Assembly_GCA_050948045_Trichoderma-sp-SZMC-28015-SZMC-28015
Assembly_GCA_051819865_Trichoderma-afarasin-CBS-130755
Assembly_GCA_050948025_Trichoderma-camerunense-CBS-138272
Assembly_GCA_020045945_Trichoderma-semiorbis-FJ059
Assembly_GCA_001990665_Trichoderma-harzianum-B97
Assembly_GCF_003025095_Trichoderma-harzianum-CBS-226-95-CBS-226-95
Assembly_GCA_043381675_Trichoderma-sp-PKRF1-PKRF1
Assembly_GCA_025502635_Trichoderma-harzianum-PAR3
Assembly_GCA_029844195_Trichoderma-harzianum-IOC-3844
Assembly_GCA_019393575_Trichoderma-harzianum-P49P11
Assembly_GCA_002838845_Trichoderma-harzianum-TR274
Assembly_GCA_029844175_Trichoderma-sp-CBMAI-0179-CBMAI-0179
Assembly_GCA_044337915_Trichoderma-harzianum-BOL-12QD
Assembly_GCF_028502605_Trichoderma-breve-T069
Assembly_GCA_017311225_Trichoderma-brevicrassum-TC967
Assembly_Trichoderma_inhamatum-CBS273-78_Scaffolds
Assembly_GCA_026401285_Trichoderma-afroharzianum-BJ182-31
Assembly_GCA_026401295_Trichoderma-afroharzianum-BJ182-30
Assembly_GCA_026401175_Trichoderma-afroharzianum-BJ232-4
Assembly_GCA_000988865_Trichoderma-harzianum-T6776
Assembly_GCA_044646125_Trichoderma-harzianum-S19
Assembly_GCA_044646245_Trichoderma-harzianum-S12
Assembly_GCA_020736905_Trichoderma-afroharzianum-IIPRTh-33
Assembly_GCA_010015525_Trichoderma-harzianum-T11-W
Assembly_GCA_026003955_Trichoderma-harzianum-T4
Assembly_GCA_037893235_Trichoderma-afroharzianum-Th6
Assembly_GCA_023628795_Trichoderma-harzianum-PB4401
Assembly_GCA_003439915_Trichoderma-atrobrunneum-ITEM-908
Assembly_GCA_050948275_Trichoderma-sp-SK1-7-SK1-7
Assembly_Trichoderma_amazonicum-CBS121216_Scaffolds
Assembly_Trichoderma_amazonicum-IB52_Scaffolds
Assembly_GCA_001721665_Trichoderma-pleuroti-TPhu1
Assembly_GCA_002894145_Trichoderma-harzianum-Tr1
Assembly_GCA_050703535_Trichoderma-pleuroticola-CBS-121217
Assembly_GCA_051820635_Trichoderma-compactum-CBS-121218
Assembly_GCA_050948005_Trichoderma-sp-SZMC-28013-SZMC-28013
Assembly_Trichoderma_agressivum-SzMC3109_Scaffolds
Assembly_GCF_033847375_Trichoderma-aggressivum-f-europaeum-CBS-100526
Assembly_Trichoderma_alni-TUCIM2657_Scaffolds
Assembly_GCA_051820675_Trichoderma-velutinum-SZMC-28001
Assembly_Trichoderma_cerinum-TUCIM2977_Scaffolds
Assembly_GCA_000800515_Trichoderma-virens-FT-333-FT-333
Assembly_GCF_000170995_Trichoderma-virens-Gv29-8-Gv29-8
Assembly_GCA_020647635_Trichoderma-virens-Gv29-8
Assembly_GCA_026401235_Trichoderma-virens-BJ192-45
Assembly_GCA_007896495_Trichoderma-viride-Tv-1511
Assembly_GCA_023634465_Trichoderma-virens-T2C1-4
Assembly_GCA_020647705_Trichoderma-virens-FT-333-FT-333
Assembly_GCA_036169685_Trichoderma-virens-G-41
Assembly_GCA_036169675_Trichoderma-viride-OSK-36
Assembly_GCA_001835465_Trichoderma-virens-IMI-304061
Assembly_GCA_051820575_Trichoderma-chlorosporum-CBS-114231
Assembly_Trichoderma_spirale-MS79_Scaffolds
Assembly_GCA_010015515_Trichoderma-sp-TW21990-1-TW21990-1
Assembly_Trichoderma_strictipile-CBS347-93_Scaffolds
Assembly_Trichoderma_helicum-DAOM230021_Scaffolds
Assembly_GCA_051820595_Trichoderma-barbatum-CBS-125733
Assembly_GCA_000332775_Trichoderma-longibrachiatum-SMF2-SMF2
Assembly_GCA_050437055_Trichoderma-longibrachiatum-Tr-8
Assembly_GCA_046056035_Trichoderma-sp-CAU-M14-CAU-M14
Assembly_GCA_026401255_Trichoderma-longibrachiatum-BJ73-36
Assembly_GCA_026259275_Trichoderma-longibrachiatum-FL-4
Assembly_GCA_046056055_Trichoderma-longibrachiatum-CAU-M23
Assembly_GCA_037041935_Trichoderma-longibrachiatum-R6902
Assembly_GCA_023628815_Trichoderma-longibrachiatum-PYS2810
Assembly_GCA_003025155_Trichoderma-longibrachiatum-ATCC-18648-ATCC-18648
Assembly_GCA_050947385_Trichoderma-longibrachiatum-CBS-115340
Assembly_GCA_001950475_Trichoderma-koningii-JCM-1883
Assembly_GCA_051820435_Trichoderma-asperellum-T28
Assembly_GCA_030770505_Trichoderma-longibrachiatum-GEV-3550
Assembly_GCA_026225735_Trichoderma-reesei-5001
Assembly_GCA_026225705_Trichoderma-reesei-5029
Assembly_Unknown_CB2_Trichoderma-sp
Assembly_GCA_021186515_Trichoderma-harzianum-ZL-811
Assembly_GCA_051819885_Trichoderma-aethiopicum-CBS-130628
Assembly_GCA_023628735_Trichoderma-sp-PF4103-PF4103
Assembly_GCA_000513815_Trichoderma-reesei-RUT-C-30-RUT-C-30
Assembly_GCA_047716485_Trichoderma-reesei-SATIMD-sor-2024
Assembly_GCF_000167675_Trichoderma-reesei-QM6a-QM6a
Assembly_GCA_047716435_Trichoderma-reesei-SATIMD-ap1-2024
Assembly_GCA_038428105_Trichoderma-reesei-Br-TrR03
Assembly_GCA_016806755_Trichoderma-reesei-CBS999-97
Assembly_GCA_052818895_Trichoderma-reesei-MA2
Assembly_GCA_030708105_Trichoderma-sp-CBMAI-0711-CBMAI-0711
Assembly_GCA_001050175_Trichoderma-parareesei-CBS-125925
Assembly_GCA_033030045_Trichoderma-parareesei-N4-3
Assembly_GCA_051819905_Trichoderma-gracile-CBS-130714
Assembly_GCA_002002365_Trichoderma-gracile-HK011-1
Assembly_GCF_004382785_Trichoderma-ghanense-CCMA-1212
Assembly_Trichoderma_effusum-DAOM230007_Scaffolds
Assembly_GCA_022813565_Trichoderma-citrinoviride-FJI-L10-BK-P3
Assembly_GCA_022813585_Trichoderma-citrinoviride-FJI-L10-BK-P1
Assembly_GCA_943193705_Trichoderma-citrinoviride-NA
Assembly_GCF_003025115_Trichoderma-citrinoviride-TUCIM-6016
Assembly_GCA_052425715_Trichoderma-citrinoviride-EB-13
Assembly_GCA_047301815_Trichoderma-citrinoviride-GF-11
Assembly_Trichoderma_flagellatum-TUCIM3334_Scaffolds
Assembly_Trichoderma_sinense-DAOM230004_Scaffolds
Assembly_GCA_050948085_Trichoderma-novae-zelandiae-CBS-472-97
Assembly_GCA_053477205_Trichoderma-deliquescens-Elizabeth-river
Assembly_GCA_025919625_Trichoderma-arundinaceum-TP19-13
Assembly_GCA_026401185_Trichoderma-arundinaceum-BJ183-35
Assembly_GCA_025919665_Trichoderma-arundinaceum-TP15-11
Assembly_GCA_025919645_Trichoderma-arundinaceum-TP6-6
Assembly_GCA_003012085_Trichoderma-brevicompactum-IBT-40841
Assembly_GCA_020631695_Trichoderma-cornu-damae-KA19-0412C
Assembly_GCA_048182565_Trichoderma-asperellum-TRC-03
Assembly_GCA_051820545_Trichoderma-sp-T77-T77
Assembly_GCA_037954425_Trichoderma-asperellum-TCTP001
Assembly_GCA_021066465_Trichoderma-asperelloides-T203
Assembly_GCA_036173025_Trichoderma-asperellum-ICC-012
Assembly_GCA_037041575_Trichoderma-asperelloides-PG5903
Assembly_GCA_052058355_Trichoderma-asperellum-TA1
Assembly_GCA_052084505_Trichoderma-asperelloides-X01119
Assembly_GCA_054127785_Trichoderma-asperellum-T285
Assembly_GCA_026401225_Trichoderma-asperelloides-BJ152-42
Assembly_GCA_037893215_Trichoderma-viride-Th4
Assembly_GCA_023628755_Trichoderma-asperellum-PT3102
Assembly_GCA_013423425_Trichoderma-asperellum-IC-1
Assembly_GCA_019481625_Trichoderma-asperellum-TAIK5
Assembly_GCA_004154885_Trichoderma-asperellum-Ts93
Assembly_GCA_014839745_Trichoderma-asperellum-SM-12F1
Assembly_GCA_019594925_Trichoderma-asperellum-TAIK4
Assembly_GCA_045838185_Trichoderma-yunnanense-TM10
Assembly_GCA_022817925_Trichoderma-asperellum-PK1J2
Assembly_GCA_037177835_Trichoderma-asperellum-CKCT-S-1
Assembly_GCA_000733085_Trichoderma-asperellum-B05
Assembly_GCA_034769445_Trichoderma-asperellum-TGT2021
Assembly_GCA_020736895_Trichoderma-asperellum-IIPRTh-31
Assembly_GCA_019594945_Trichoderma-asperellum-TAIK1
Assembly_GCF_003025105_Trichoderma-asperellum-CBS-433-97-CBS-433-97
Assembly_GCA_026401105_Trichoderma-asperellum-CGMCC-11653
Assembly_GCF_020647865_Trichoderma-asperellum-FT101
Assembly_GCA_049345875_Trichoderma-asperellum-CGMCC-41372
Assembly_GCA_017945965_Trichoderma-asperellum-DQ-1
Assembly_GCA_039880885_Trichoderma-asperellum-FT101
Assembly_GCA_022817935_Trichoderma-sp-MLT1J1-MLT1J1
Assembly_GCA_046127165_Trichoderma-hamatum-Z11
Assembly_GCA_044646315_Trichoderma-harzianum-S5
Assembly_GCA_054127445_Trichoderma-hamatum-Ta8426
Assembly_GCA_000331835_Trichoderma-hamatum-GD12-GD12
Assembly_GCA_049996655_Trichoderma-hamatum-G765-22
Assembly_GCA_046629995_Trichoderma-hamatum-FBL-Z32
Assembly_GCA_050947405_Trichoderma-evansii-SZMC-28006
Assembly_GCA_022985005_Trichoderma-koningiopsis-RA3a
Assembly_GCA_022984945_Trichoderma-koningiopsis-RA6
Assembly_GCA_022984975_Trichoderma-koningiopsis-RA5
Assembly_GCA_030247165_Trichoderma-koningiopsis-TK1
Assembly_GCA_002246955_Trichoderma-koningiopsis-POS7
Assembly_Trichoderma_taiwanense-IQ11_Scaffolds
Assembly_GCA_049996695_Trichoderma-koningiopsis-G756-22
Assembly_GCA_048164925_Trichoderma-koningiopsis-Z35
Assembly_GCA_046255345_Trichoderma-koningiopsis-T-51
Assembly_GCA_038502285_Trichoderma-sp-2475-2475
Assembly_GCA_046580765_Trichoderma-virilente-Z53
Assembly_GCA_049996625_Trichoderma-koningiopsis-G779-22
Assembly_GCA_051820695_Trichoderma-sp-SZMC-28014-SZMC-28014
Assembly_GCA_051820155_Trichoderma-sp-TUCIM-5745-TUCIM-5745
Assembly_GCA_049996675_Trichoderma-gamsii-G762-22
Assembly_GCA_002894205_Trichoderma-gamsii-A5MH
Assembly_GCF_001481775_Trichoderma-gamsii-T6085
Assembly_GCA_052570025_Trichoderma-viride-329365
Assembly_GCA_052571625_Trichoderma-viride-315707
Assembly_GCA_051527675_Trichoderma-atroviride-CBS-122147
Assembly_GCA_000963795_Trichoderma-atroviride-XS2015
Assembly_GCF_020647795_Trichoderma-atroviride-P1
Assembly_GCA_019297715_Trichoderma-atroviride-IMI-206040
Assembly_GCA_001599035_Trichoderma-atroviride-JCM-9410
Assembly_GCA_051557965_Trichoderma-atroviride-B7
Assembly_GCA_041053265_Trichoderma-atroviride-CGMCC-40488
Assembly_GCA_020466355_Trichoderma-atroviride-CG-6828
Assembly_GCA_028554805_Trichoderma-atroviride-Italy
Assembly_GCA_040333345_Trichoderma-atroviride-F020
Assembly_GCA_002916895_Trichoderma-atroviride-LY357
Assembly_GCA_030708095_Trichoderma-sp-CBMAI-0020-CBMAI-0020
Assembly_GCA_052570065_Trichoderma-viride-238904
Assembly_GCA_052570055_Trichoderma-viride-309685
Assembly_GCA_026401215_Trichoderma-erinaceum-BJ212-21
Assembly_GCA_013365115_Trichoderma-erinaceum-CRRI-T2N1
Assembly_Trichoderma_strigosellum-IQ191_Scaffolds
Assembly_GCA_051820515_Trichoderma-austrokoningii-CBS-119092
Assembly_Trichoderma_minutisporum-DAOM175931_Scaffolds
Assembly_GCA_052570145_Trichoderma-viride-90312
Assembly_GCA_030278935_Trichoderma-polysporum-HZ-31
Assembly_GCA_051819845_Trichoderma-ceciliae-CBS-130010
Assembly_GCA_015266385_Trichoderma-oligosporum-CGMCC-3-17527
Assembly_GCA_023653515_Trichoderma-orchidacearum-COAD-3006
