# Supplementary material for: Phylogenomic and Comparative Genomic Analyses Reveal Deep Evolutionary Structure and Cryptic Diversity in the Genus Trichoderma
Source: Int J Mol Sci. 2026 Sep 15;27(18):8204. doi: 10.3390/ijms27188204 (PMC13607837; doi:10.3390/ijms27188204)
Supplement: Supplementary file 1 [file ijms-27-08204-s001.zip › Supp_Fig_S4.pdf]

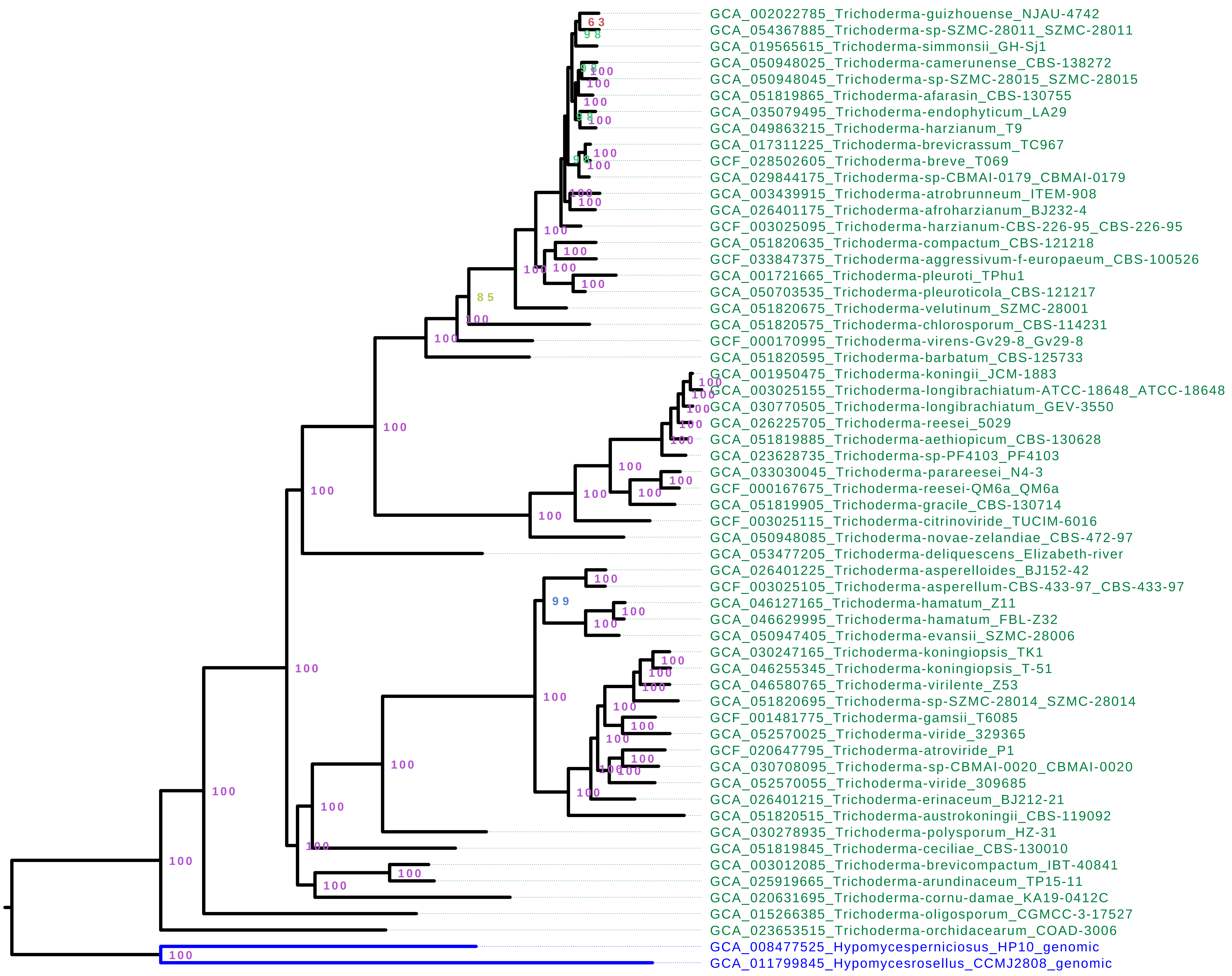
GCA_002022785_Trichoderma-guizhouense_NJAU-4742
GCA_054367885_Trichoderma-sp-SZMC-28011_SZMC-28011
GCA_019565615_Trichoderma-simmonsii_GH-Sj1
GCA_050948025_Trichoderma-camerunense_CBS-138272
GCA_050948045_Trichoderma-sp-SZMC-28015_SZMC-28015
GCA_051819865_Trichoderma-afarasin_CBS-130755
GCA_035079495_Trichoderma-endophyticum_LA29
GCA_049863215_Trichoderma-harzianum_T9
GCA_017311225_Trichoderma-brevicrassum_TC967
GCF_028502605_Trichoderma-breve_T069
GCA_029844175_Trichoderma-sp-CBMAI-0179_CBMAI-0179
GCA_003439915_Trichoderma-atrobrunneum_ITEM-908
GCA_026401175_Trichoderma-afroharzianum_BJ232-4
GCF_003025095_Trichoderma-harzianum-CBS-226-95_CBS-226-95
GCA_051820635_Trichoderma-compactum_CBS-121218
GCF_033847375_Trichoderma-aggressivum-f-europaeum_CBS-100526
GCA_001721665_Trichoderma-pleuroti_TPhu1
GCA_050703535_Trichoderma-pleuroticola_CBS-121217
GCA_051820675_Trichoderma-velutinum_SZMC-28001
GCA_051820575_Trichoderma-chlorosporum_CBS-114231
GCF_000170995_Trichoderma-virens-Gv29-8_Gv29-8
GCA_051820595_Trichoderma-barbatum_CBS-125733
GCA_001950475_Trichoderma-koningii_JCM-1883
GCA_003025155_Trichoderma-longibrachiatum-ATCC-18648_ATCC-18648
GCA_030770505_Trichoderma-longibrachiatum_GEV-3550
GCA_026225705_Trichoderma-reesei_5029
GCA_051819885_Trichoderma-aethiopicum_CBS-130628
GCA_023628735_Trichoderma-sp-PF4103_PF4103
GCA_033030045_Trichoderma-parareesei_N4-3
GCF_000167675_Trichoderma-reesei-QM6a_QM6a
GCA_051819905_Trichoderma-gracile_CBS-130714
GCF_003025115_Trichoderma-citrinoviride_TUCIM-6016
GCA_050948085_Trichoderma-novae-zelandiae_CBS-472-97
GCA_053477205_Trichoderma-deliquescens_Elizabeth-river
GCA_026401225_Trichoderma-asperelloides_BJ152-42
GCF_003025105_Trichoderma-asperellum-CBS-433-97_CBS-433-97
GCA_046127165_Trichoderma-hamatum_Z11
GCA_046629995_Trichoderma-hamatum_FBL-Z32
GCA_050947405_Trichoderma-evansii_SZMC-28006
GCA_030247165_Trichoderma-koningiopsis_TK1
GCA_046255345_Trichoderma-koningiopsis_T-51
GCA_046580765_Trichoderma-virilente_Z53
GCA_051820695_Trichoderma-sp-SZMC-28014_SZMC-28014
GCF_001481775_Trichoderma-gamsii_T6085
GCA_052570025_Trichoderma-viride_329365
GCF_020647795_Trichoderma-atroviride_P1
GCA_030708095_Trichoderma-sp-CBMAI-0020_CBMAI-0020
GCA_052570055_Trichoderma-viride_309685
GCA_026401215_Trichoderma-erinaceum_BJ212-21
GCA_051820515_Trichoderma-austrokoningii_CBS-119092
GCA_030278935_Trichoderma-polysporum_HZ-31
GCA_051819845_Trichoderma-ceciliae_CBS-130010
GCA_003012085_Trichoderma-brevicompactum_IBT-40841
GCA_025919665_Trichoderma-arundinaceum_TP15-11
GCA_020631695_Trichoderma-cornu-damae_KA19-0412C
GCA_015266385_Trichoderma-oligosporum_CGMCC-3-17527
GCA_023653515_Trichoderma-orchidacearum_COAD-3006
GCA_008477525_Hypomycespernicio sus_HP10_genomic
GCA_011799845_Hypomycesrosellus_CCMJ2808_genomic
